# Stochastic simulation and analysis of biomolecular reaction networks

**DOI:** 10.1186/1752-0509-3-64

**Published:** 2009-06-17

**Authors:** John M Frazier, Yaroslav Chushak, Brent Foy

**Affiliations:** 1 Human Effectiveness Directorate (AFRL/REPB), Air Force Research Laboratory, WPAFB, OH, USA, 45433-5707; 2 Biotechnology HPC Software Applications Institute, Telemedicine and Advanced Technology Research Center U.S. Army Medical Research and Materiel Command, Fort Detrick, MD, USA, 21702; 3 Department of Physics, Wright State University, Dayton, OH, USA, 45435

## Abstract

**Background:**

In recent years, several stochastic simulation algorithms have been developed to generate Monte Carlo trajectories that describe the time evolution of the behavior of biomolecular reaction networks. However, the effects of various stochastic simulation and data analysis conditions on the observed dynamics of complex biomolecular reaction networks have not recieved much attention. In order to investigate these issues, we employed a a software package developed in out group, called Biomolecular Network Simulator (BNS), to simulate and analyze the behavior of such systems. The behavior of a hypothetical two gene *in vitro *transcription-translation reaction network is investigated using the Gillespie exact stochastic algorithm to illustrate some of the factors that influence the analysis and interpretation of these data.

**Results:**

Specific issues affecting the analysis and interpretation of simulation data are investigated, including: (1) the effect of time interval on data presentation and time-weighted averaging of molecule numbers, (2) effect of time averaging interval on reaction rate analysis, (3) effect of number of simulations on precision of model predictions, and (4) implications of stochastic simulations on optimization procedures.

**Conclusion:**

The two main factors affecting the analysis of stochastic simulations are: (1) the selection of time intervals to compute or average state variables and (2) the number of simulations generated to evaluate the system behavior.

## Background

All biological processes at the cellular level are the consequence of a series of chemical-physical reactions at the molecular level that occur within the micro-volume of the cell. The collection of molecular species and the reactions among them is referred to here as a 'biomolecular reaction network'. The complete biomolecular reaction network for a cell includes thousands of molecular components and reactions involved in transcription, translation, molecular self-assembly, metabolic reactions, transport and physical movements. Since these reactions occur in an extremely small reaction volume, the number of molecules of any one molecular species that can participate in a given reaction ranges from single copies of genes to several hundred molecules of chemicals at the *μ*M concentration to several hundred thousand molecules of chemicals at the mM concentration. As a consequence of the fact that a subset of all the reactions in the system involve low copy numbers of substrate molecules, the behavior of individual instances of the system cannot be modeled accurately using continuous deterministic (C-D) approaches ([1-3]). Thus, these natural micro-systems should be modeled and simulated using basic theory of discrete stochastic (D-S) chemical kinetics [4].

With the evolution of systems biology in recent years, interest in modeling and simulating the behavior of engineered genetic circuits in bacterial cells has increased [5]. In addition to living cells, nano-biotechnology researchers are exploring the possibility of developing and using artificial cellular constructs employing natural and engineered biological processes ([6-11]). In order to predict the behavior of these constructs, modeling and simulation of their biomolecular reaction networks are needed to enable the design and fabrication of both the constructs themselves and physical devices based on these constructs.

Using stochastic chemical kinetics for exact simulations of biomolecular reaction networks presents several computational challenges. The ultimate goals of the simulation exercise are to be able to: (1) model and simulate the behavior of the system using a complete and accurate physical and mathematical description of the system and an exact simulation algorithm, (2) generate large numbers of simulations, and (3) analyze the data in a meaningful way. All of these goals should be accomplished in a reasonable amount of computational time. The main factors that determine the computational challenge of a particular simulation activity are the size of the model (how many molecular species and reactions are involved), the nature of the reactions (stiffness – mixture of fast and slow reactions), the duration of the simulation, the number of simulations required for statistical significance, the data logging requirements, and, data analysis requirements. Depending on the computational dimensions of the problem, the ultimate goals of the simulation exercise, as defined above, may be attainable. However, as the computational dimension increases, the ability to meet all of the requirements of the ultimate solution becomes more difficult.

There are two approaches that can be taken to address the more difficult computing problems associated with larger computational dimensions. The first approach is to employ approximations at various levels of the modeling and simulation process. At the conceptual model level, detailed reaction mechanisms consisting of multiple micro-reactions can be replaced with approximate lumped macro-reactions. This has the effect of reducing the number of both molecular species and reactions in the model. At the simulation level, there are approximate stochastic simulation algorithms, such as *τ*-leaping [12], that can speed-up the simulation time, but at the expense of accuracy. At the statistical level, the accuracy of the statistical properties of the ensemble of system simulations, computed from the simulation data, increases as the number of simulations increases, ultimately approaching the statistical properties of the exact solution in the limit as the number of simulations increases to infinity. Thus, truncating the number of simulations to decrease the computational time results in less accurate approximations of the statistical properties of the system. Finally, the collection and storage of the raw simulation data will affect the computational time. The maximum information obtainable from a simulation run requires the collection of the time and nature of every reaction event. Limiting the amount of data collected will reduce computation time at the expense of the types and accuracy of subsequent data analyses.

The second approach is to run simulations on bigger and more powerful computer hardware using software designed to take advantage of multiprocessors. Although the exact stochastic simulation algorithms do not lend themselves to efficient parallelization of the algorithm itself, running multiple simulations on separate processors can reduce the overall time required to generate a statistically adequate ensemble of independent simulations. This approach does not reduce the actual computational time, but, by running multiple simulations in parallel, it does allow one to reduce the clock time required to obtain a sufficient number of simulations for appropriate statistical analysis. In addition, numerical analysis of the large data sets generated by the simulation can be parallelized to speed up this time consuming step. Overall, there are a range of trade-offs between simulation strategy, data accuracy and computational time that must be taken into consideration when optimizing the modeling, simulation and analysis of biomolecular reaction networks for particular applications.

In recent years, single cell experiments have become a significant focus of the experimental approach to problems in systems biology. Analysis of data derived from such experiments should be interpreted in the context of stochastic systems and we feel that it is of practical value to the broader community of researchers to present concrete examples of these issues using a hypothetical model that is relevance to the fundamental transcription-translation-metabolism scheme. To model and simulate the behavior of these systems, various software packages have been developed and released to the general public (e.g., [13-19]). Each of these software products has its advantages and disadvantages for different modeling needs. We developed a software package – the Biomolecular Network Simulator (BNS) – that is specifically designed to operate on either single or multiple processor hardware [20]. The BNS software allows one to build a model of a synthetic biomolecular reaction network and to investigate its behavior using several different stochastic algorithms. Here we use the BNS software to investigate the effects of various external conditions, such as selecting: (1) the observation interval, (2) the time-averaging interval, or (3) the number of simulation, on the observed behavior of a hypothetical, yet relatively complex, biomolecular reaction network involving transcription, translation and metabolism to illustrate some of the unique data analysis issues that arise in stochastic simulations of biomolecular reaction networks. It is hoped that the reader will gain a better intuitive understand of how these factors can influence the interpretation of stochastic reaction systems.

## Methods

### Stochastic Simulation Algorithm

The mathematical description of the behavior of stochastic biomolecular reaction networks is based on Markov process theory [21]. The system behaves as a multi-variant, discrete state, Markov jump process and is governed by the chemical master equation (CME). The solution of the CME is in fact the mathematically exact description of the behavior of the system. For our purposes, we will consider a biomolecular reaction network consisting of *N*_*S *_identifiable molecular species, denoted *S*_*i *_(i = 1, 2, ..., *N*_*S*_). These molecular species can undergo *N*_*R *_fundamental chemical reactions *r*_*k *_(*k *= 1, 2, ..., *N*_*R*_) and are confined to a fixed reaction volume, *V*_*R*_. It is assumed that the system is well-mixed (homogenous) and at constant volume and temperature. Let ***s***(*t*) be an *N*_*S*_-dimensional state vector whose elements *s*_*i*_(*t*) (*i *= 1, 2, ..., *N*_*S*_) are the number of molecules in the system of each molecular species *S*_*i *_at time *t*.

The stochastic process that describes the behavior of the biomolecular reaction network is characterized by the state probability density function *P*(*s*, *t*). This function gives the probability that the system is in state ***s ***at time *t*, where ***s ***can take on any value in the allowable state space. *P*(*s*; *t*) is the solution of the CME [4]:

(1)

where *a*_*k *_(***s***, *t*) is the propensity of the *k*^*th *^fundamental reaction at time *t *and ***ν***^*k *^is the state change vector, a *N*_*S*_-dimensional vector that specifies the changes in the number of molecules of each state variable when the *k*^*th *^reaction occurs. Note, the sum is over all of the *N*_*R *_possible reactions that can occur. Further note, the propensity for a given reaction, *a*_*k*_(***s***, *t*), is computed as the product of the reaction probability constant, *c*_*k*_, and the total number of combinations of possible reacting molecules for that reaction. The reaction probability constant is, in a sense, a measure of the reactivity of the reaction substrates [4].

The specification of the initial condition for the biomolecular reaction network of interest, *P*_0_(***s***) = *P*(***s***_0_, *t *= 0), depends on the precision and accuracy of the measurement techniques used to experimentally characterize the system. In theory, the system is in a single well defined state ***s***_**0 **_at time *t*_0_, where the number of molecules of each molecular species is equal to the exact number of molecules of that species contained in the reaction volume *V*_*R *_at time *t*_0_. In this case, *P*_0_(***s***) is defined by the Kronecker delta function as

(2)

For our purposes, it will be assumed that the initial condition as defined by Equation (2) will hold and the state density function that is the solution of the CME can be written as the conditional probability density function *P*(***s***, *t*|***s***_0_, *t*_0 _= 0).

Usually, an analytical solution of the CME is not possible and direct numerical computation of the solution is computationally overwhelming due to the large state space. However, the direct simulation of exact (theoretically possible) trajectories in state space is feasible [21]. The time evolution of the state vector ***s***(*t*) for a theoretically possible instance of the system can be calculated using various algorithms proposed for Monte Carlo simulations of stochastic trajectories. The Gillespie direct stochastic algorithm [4] is used in this report to illustrate the stochastic behavior of a gene expression system. The Gillespie direct stochastic algorithm theoretically generates exact simulations of system trajectories in state space if and only if all reactions in the biomolecular reaction network are fundamental reactions [4]. In the limit of an infinite number of simulations, the statistical properties of the ensemble of exact simulations approaches those of the exact solution of the CME, i.e., for the first moment (mean) of ***s ***(⟨***s***(*t*)⟩) we have

(3)

where ***s***^***i ***^(*t*) is the value of the state vector at time *t *in the *ith *simulation run, ⟨*s*(*t*)⟩_*n *_is the estimate of the mean state vector at time *t *based on an ensemble of *n *simulations, the left hand sum is over all possible states in state space and the right-hand sum is over all values of the state vector at time *t *observed in the *n *simulation runs. In addition, the second moment of the probability density function for the state vector ***s ***is related to the variance by the relationship

(4)

where the variance of ***s ***is

(5)

and *σ*_*n *_(*t*) is the estimate of the standard deviation (SD) based on the ensemble of *n *simulations. Thus, computing the mean and SD of the ensemble of simulations at a given time provide an estimate of the first and second moments of the probability density function for the system at that time.

Although the basic biochemical reactions in a biomolecular reaction network are treated as discrete, jump Markov processes and thus stochastic in nature, if the number of molecules of every species in the system is large then the process can be approximated by a continuous Markov process [21]. Furthermore, if the number of molecules and the volume increase in proportion such that the concentration of each species is constant (the so-called thermodynamic limit), then the solution describing the behavior of the state variables can be written as the sum of a single-valued variable that is the solution of the classical rate equations and a variable factor that decreases in magnitude as . Thus, for sufficiently large reaction volume, keeping concentrations constant (consequently large number of molecules), the first moment of the probability density function of the state variables approaches the classical continuous deterministic solution of the reaction rate ODEs. However, if there are only a few molecules of any given species, as is often the case in gene expression, this approximation will not accurately describe the instantaneous state of the system. Furthermore, the C-D approach will provide no information concerning the temporal fluctuations of state variables of a given system nor the variability between multiple instantiations of the system with identical initial conditions.

### Biomolecular Network Simulator Software

The Biomolecular Network Simulator software was developed to allow for stochastic simulations on either desktop or multi-processor hardware (see **age: ** for complete documentation of the software and  to download the software). The front-end graphical users interface (GUI) and the backend data analysis tools are written in MATLAB. This allows the user to exploit the interactive features and visualization tools of MATLAB for setting up simulations and analyzing and interpreting the resulting data. The simulation engine itself and the analysis tools are written in the C language to maximize speed for the computationally intensive part of a simulation run and post-simulation data analysis.

The BNS software accepts two types of model definitions: (1) Systems Biology Markup Language (SBML) format [22] and (2) BNS format where models are defined by a set of MATLAB m-files. There are two types of output files: snapshot data and event log data. Snapshot data files contain the state of the system (number of molecules of each molecular species that are selected to be monitored) and the number of reaction occurrences in each reaction channel since the last snapshot at user specified time intervals. The second type of output files – the event log files – contain the record of every discrete event that occurs during the simulation (i.e., the reaction name and time of each and every event).

The BNS software has a comprehensive set of tools for post-simulation analyses. The most frequently used type of analysis is to plot the number of molecules of a particular molecular species versus time. The number of molecules of a particular molecular species versus time plots can be created with both types of output files, snapshot data or event log data, with the event log data giving an exact description of the behavior of the selected state variable. A time-weighted average analysis provides for the calculations of the average number of molecules of a particular molecular species during a user selected time-interval. The time averaged number of molecules of state variable *k *over the interval Δ*t *at time *t*_*i*_, ⟨*s*_*k*_(*t*_*i*_)⟩_Δ*t*_, is given by:

(6)

where

(7)

*t*_*i*, *j *_is the *j*^*th *^time subinterval in the interval *t*_*i *_to *t*_*i*_+ Δ*t *and the summation is over , the total number of reaction events in the interval *t*_*i *_to *t*_*i*_+ Δ*t *that affect *s*_*k*_. The computation is accomplished by summing up the products of the time sub-intervals multiplied by the number of molecules present in that sub-interval, thus the average is weighted according to the amount of time the compound exists in each state during the selected time-interval. The averaging analysis can be performed for a single simulation run or for an ensemble of runs. In the latter case, the between run average (the average of the individual time-weighted averages over the ensemble of simulation runs) and standard deviation are plotted.

When multiple simulations are run, the distribution of state variables for the ensemble of simulations at a given time can be investigated by plotting a discrete histogram. The data to generate these discrete histograms can be extracted from data files saved by the analysis tools in the BNS Toolbox. The distribution for a particular state variable *k *at time *t*_*i*_, , is computed by counting the number of times each possible state *j *for that state variable is occupied at time *t*_*i *_in the ensemble of simulations () and dividing by the total number of simulations (*n*_*s*_).

(8)

The data are plotted with the *y*-axis representing the fraction of the simulations that the state variable was in the discrete state indicated on the *x*-axis. This is an estimate of the probability that the system would be in that particular state at the defined time *t*_*i*_, i.e., the state probability density *P*(*s*, *t*|*s*_0_, *t*_0_) defined by Equation 1.

Complex biomolecular reaction networks that involve gene expression are usually stiff systems, i.e., contain reactions that occur on widely different time scales; some reactions have a low propensity and occur rarely while other reactions have a high propensity and occur frequently. A unique feature of the BNS software is that the data stored allows the user to perform various reaction event rate analyses on the simulation data to learn more about the basic nature of the reactions in the system. The time-averaged reaction event rate in reaction channel q (number of *q *reaction events per unit time) can be calculated for a user-selected time-averaging intervals Δ*t *by:

(9)

where  is the number of reaction events in the time interval *t*_*i *_to *t*_*i*_+ Δ*t*. These analyses provide important information about the behavior of the system, e.g., relative event rates for important reactions. Such event rate data can be used to calculate the rate of substrate utilization in selected reaction channels as a function of the state of the system.

The BNS software can be run on high performance computing (HPC) hardware. Parallelization of the BNS code for simulation runs on HPC hardware is accomplished using the Message Passing Interface (MPI). In our parallelization scheme, the 'master' processor divides the total number of simulation runs into a set of jobs depending on the number of available processors and sends a job to each of the 'worker' processors. The snapshot data from the workers are sent back to the master processor for the interactive graphics while the event log files are saved directly to the hard drive by the workers. In this approach to parallelization, the power of parallel processing is utilized to run a large number of simulations simultaneously and thus speedup the overall clock time for large batch jobs.

### Exemplar model

In order to investigate the analysis of a discrete stochastic system, a hypothetical model of a generic two gene, self-assembling catalytic ligation reaction in a cell-free transcription-translation (CFTT) system is explored. The hypothetical biomolecular reaction network consists of the transcription and translation reactions of two genes in a gene expression system and the subsequent metabolic reactions of the expressed enzymes. The system is assumed to be a closed system contained in a vesicle with a reaction volume of 5 × 10^-16 ^L corresponding to a spherical vesicle with a diameter of approximately 1.0 *μ*m. Note, in such a vesicle, a molecular species at a concentration of 1 *μ*M is equivalent to a total of 301 molecules present in the vesicle. The CFTT system contains all of the necessary components for transcription and translation of target genes into the expressed proteins.

**Figure 1:**
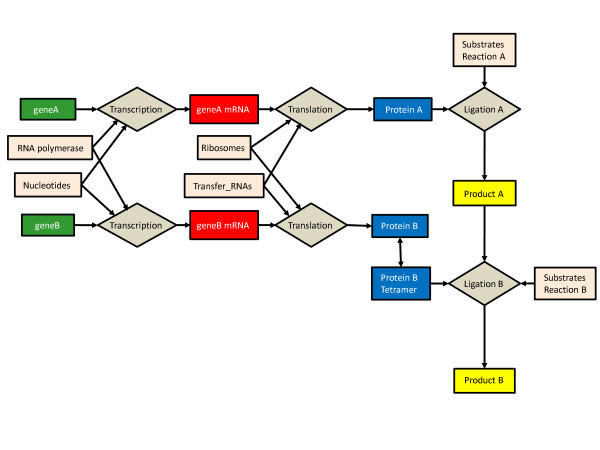
**Process diagram of the two gene biomolecular reaction network used as an exemplar model**. For a detailed description of the exemplar model see Additional File 1.

To formulate a conceptually simple, yet biochemically reasonable, model of the kinetics of the self-assembly of the exemplar biomolecular reaction network and the subsequent metabolic reactions, the high level conceptual system model illustrated in Figure 1 was proposed. The detailed model of the system consists of 249 state variables and 287 reactions (see Additional File 1 for a complete description of the model and Additional File 2 for the SBML model description). In the detailed conceptual diagram of the system, state variables are labeled with names that are descriptive of the molecules that they represent. However, internally in the SBML model code the state variables are labeled *s001 *to *s249*. Wherever there may be confusion in the discussion below, both labels are used for clarity.

Transcription of *geneA *and *geneB *consists of four reactions each. The four reactions are the association and dissociation of the RNA polymerase (*RNAp *– *s001*) and the promoter sites for *geneA *(*P_A *– *s002*) and geneB (*P_B *– *s022*) to form the promoter-polymerase complexes (*RNAp_P_A *– *s003 *and *RNAp_P_A *– *s023*). The polymerase then translocates from the promoter site to the transcription start site forming the transcription start complex (*RNAp_geneA *– *s004 *and *RNAp_geneB *– *s024*) and the subsequent formation of the mRNA (geneA_mRNA – *s009 *and geneB_mRNA – *s025*). The mRNAs can either be degraded by a generic RNase or used as a template for protein synthesis. Translation consists of three reactions that include association and dissociation of the small ribosomal unit (*Rib_s *– *s015*) with the ribosomal binding site on the mRNA to form the ribosomal-mRNA complex (*Rib_s_geneA_mRNA *– *s016 *and *Rib_s_geneB_mRNA *– *s026*) and the subsequent translation reaction resulting in the formation of the protein products (*Pro_A *– *s018 *and *Pro_B *– *s027*). Key substrates for the translation reaction are the 20 charged transfer RNAs (*AA_i_tRNA_AA_i *– *s091 *to *s111*) which are formed from the appropriate amino acids (*AA_i *– *s032 *to *s051*) and the corresponding transfer RNAs (*tRNA_AA_i *– *s072 *– *s091*) by the associated aminoacyl-transferases (*Trans_AA_i *– *s052 *to *s071*). The protein product *Pro_A *is capable of catalyzing the ligation of *Sub_1 *(*s233*) and *Sub_2 *(*s237*) to form the metabolic product *Prod_A *(*s240*) via a series of association, catalytic and dissociation reactions. The protein product *Pro_B *must form a tetramer (*Pro_B_4*) to be catalytically active. Once formed *Pro_B_4 *catalyzes the ligation of *Prod_A *and *Sub_3 *(*s245*) to form the final product *Prod_B *(*s248*) via another series of association, catalytic and dissociation reactions. All proteins can be degraded by a generic protease (*Prot *– *s031*).

In this biomolecular reaction network, the biomolecular reactions relating to the expression of *geneA *and *geneB *and subsequent metabolism are treated as stochastic in nature. Here we use the Gillespie direct stochastic algorithm to obtain sufficient numbers of probabilistically correct state space trajectories, consistent with the CME, for statistical analyses. The simulation data sets obtained through the use of these Monte Carlo simulations are used to illustrate some of the statistical properties of the stochastic behavior of the exemplar model. Note, these simulation data are for a generic model and do not necessarily represent the behavior of any actual system.

## Results and Discussion

### Simulation of exemplar model using the Gillespie Direct Algorithm

**Figure 2:**
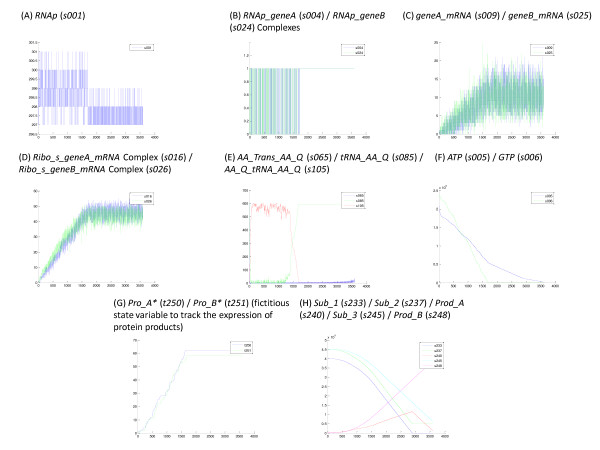
**Selected results for simulations of the exemplar model**. Plots are of a possible trajectory in state space (number of molecules of the selected state variable versus time) for a single simulation. These plots were obtained from the event log data and include every event that influenced the particular state variable. The *x*-axis is time (sec) and the *y*-axis is the number of molecules of the given state variable(s). All plots were created using the 'Create Plots from Data' tool in the BNS Analysis Toolbox.

In order to explore the general behavior of the exemplar model, a series of simulations were run using the following conditions: (1) the Gillespie direct stochastic simulation algorithm, (2) an SBML model definition (see Additional File 2 for model code), (3) the stochastic reaction parameters and initial conditions in the SBML model definition, and (4) the following simulation parameters: number of simulations = 10, duration of simulation = 3600 sec, snapshot interval (SSI) = 50 sec (giving a total of 72 snapshots), and event log = on. A limited number of simulations (n = 10) were used to obtain representative results quickly (total simulation time for 10 simulations was 365 sec). Due to the scale of the model (249 state variables), it is not possible to show the total set of data for all state variables, but a few selected and important state variables are shown in Figure 2 to illustrate the general behavior of the system. The data presented show the trajectories of the selected state variables for a single representative simulation using the event log data. These data show each and every event that affected the selected state variables and is an exact trajectory in state space. The biomolecular reaction system under investigation is a closed system with a limited supply of energy molecules and reaction substrates. The system self-assembles via transcription and translation of *geneA *and *geneB*, using the available energy and substrates, to form the metabolic reaction pathway consisting of two tandem ligation reactions that ultimately synthesize the final product, *Prod_B*. When critical substrates are depleted, reactions dependent on these substrates stop. In this particular system, three substrates, *GTP *(*s006*), *ATP *(*s005*) and *Sub_1 *(*s233*) prove to be critical (Figures 2(F) and 2(H)) although another substrate, charged tRNA for glutamine (*AA_Q_tRNA_AA_Q *– *s105*) could be critical under slightly different circumstances (Figure 2(E)). First, *GTP *is depleted at about 1700 sec. Since *GTP *is utilized by both mRNA synthesis as a substrate and protein synthesis as an energy source, both transcription of messenger RNA and translation of the protein products are simultaneously terminated at the time when *GTP *is depleted. Even if protein synthesis had not terminated at 1700 sec due to depletion of *GTP*, it would have terminated soon thereafter due to the depletion of *AA_Q_tRNA_AA_Q*. Secondly, *ATP *is not only a substrate for mRNA synthesis, but is also an energy source for the ligation reactions and charging of tRNA with amino acids. Therefore, when *ATP *is depleted at about 3600 sec, both the enzymatic ligation reactions and the tRNA charging reactions terminate. Third, the first metabolic ligation reaction terminated when *Sub_1 *was depleted at about 2900 sec and subsequently, the second metabolic ligation reaction would have terminated when all of *Prod_A *formed by the first ligation reaction was depleted. These reactions illustrate the general behavior of a closed biomolecular reaction network, reactions that depend on substrates and energetic molecules terminate when these components are depleted while simple binding reactions that operate on thermal energy continue to be executed.

**Figure 3:**
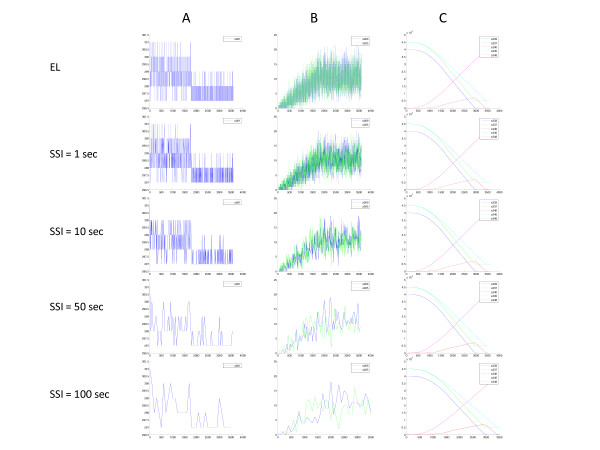
**Effect of the snapshot interval (SSI) on the appearance of the trajectories of state variables**. Individual simulations using the same random number seed were performed with different SSIs ranging from 1 sec to 100 sec. The first row is the exact trajectory using the event log data. The other plots are obtained based on the snapshot data from simulations using the indicated SSIs. The *x*-axis is the time (sec) and the *y*-axis is the number of molecules of the given state variable. All plots were created using the 'Create Plots from Data' tool in the BNS Analysis Toolbox. Column: (A) – RNA polymerase (*RNAp *– *s001*); (B) free *geneA_mRNA *(*s009*) and *geneB_mRNA *(*s024*); and (C) *Sub_1 *(*s233*), *Sub_2 *(*s237*), *Prod_A *(s240), *Sub_3 *(*s245*) and *Prod_B *(*s248*).

To generate the exact trajectories for state variables, the 'Create Plots from Data' tool in the BNS Analysis Toolbox must parse the event log to obtain the time sequence of each reaction and then create the trajectory of each state variable. For systems that involve many reactions and many state variables, this can be a computationally demanding and thus a time consuming process. To obtain a quicker but lower resolution picture of the general behavior of the system, the BNS software computes the state of each state variable at fixed time points determined by the snapshot interval (SSI) which is user defined. Figure 3 shows three of the same trajectories as in Figure 2 but using the snapshot data with SSI ranging from 1 to 100 sec. Note, these simulations were performed using the same random number seed, thus the event logs are identical for all of these simulation, only the snapshot data files change between simulations. For state variables that are experiencing rapid dynamic fluctuations (e.g., Figure 3(A), and 3(B)), it is obvious that the snapshot plots do not reveal all the details of the behavior of the state variable. For example, the instantaneous values of the free mRNAs for geneA (*geneA_mRNA*, *s009 *– Figure 3(B) blue line) fluctuate rapidly between 2 and 23 copies when transcription terminates (*t *> 1700 sec in top plot). However, inspection of the snapshot data for SSI = 50 sec during the same time interval suggests that the levels only fluctuate between 8 and 19 copies. As this series of data indicate, the snapshot data approach the exact behavior (event log data) as the SSI decreases. For this particular biomolecular reaction network, only a SSI = 1 sec gives an adequate approximation to the exact behavior of these rapidly fluctuating state variables. On the other hand, state variables that are only created (final products) or only destroyed (non-renewable substrates) do not fluctuate but merely increase or decrease monotonically (Figure 3(C)). For these state variables, a SSI = 100 sec gives an adequate approximation. For this model, a SSI = 50 sec will be adequate to give a general impression of the behavior of most state variables except for those that fluctuate rapidly. However, the ability to observe the actual range of a state variable that fluctuates rapidly will only be possible using the event log data.

**Figure 4:**
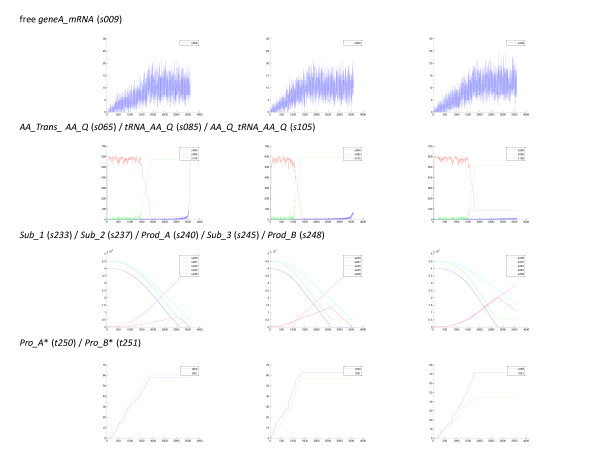
**Variability in state variable trajectories between individual simulation runs**. Individual trajectories for three separate simulations for selected state variables are plotted. The data used in creating these plots were taken from the event logs. The x-axis is time (sec) and the y-axis is the number of molecules of the given state variable. All plots were created using the 'Create Plots from Data' tool in the BNS Analysis Toolbox.

Each simulation run provides a probabilistically accurate trajectory of the system in state space. Figure 4 shows the state space trajectories of the number of molecules of several state variables for 3 individual simulations. Although each of the trajectories shown illustrates the possible behavior of the state variable that is consistent with the physical-chemical nature of the system, the likelihood that the behavior of any actual system would behave in an identical manner to a simulated trajectory is small. Thus, comparison of the data for a single simulation run with time-series experimental data from a single biomolecular reaction system would not be particularly useful, except in the sense of general trends. The value of individual simulation runs is to provide some intuitive insight into the possible range of behaviors of the system under investigation. For example, Figure 4(A) illustrates the behavior of free geneA mRNA (*geneA_mRNA *– *s009*). In all three simulations, the level of free mRNA increases while transcription is active, then levels off when *GTP *is depleted. In spite of the rapid fluctuations in the level of free mRNA, the general trend is similar in all three simulations. In Figure 4(B), the state space trajectories of the number of molecules of the free amino-acyl-transferase for the amino acid glutamine, *AA_Trans_AA_Q *(*s065*), the uncharged tRNA for glutamine, *tRNA_AA_Q *(*s085*), and the charged glutamine tRNA, *AA_Q_tRNA_AA_Q *(*s105*) is shown. In each simulation, the level of the charged tRNA fluctuates about a value of approximately 570 while it is feeding the translation reactions and then drops rapidly when glutamine is depleted. However, the timing of the rapid drop in charged tRNA and the final level of the charged tRNA varies from run-to-run depending on when *GTP *is depleted and how many protein molecules were completely synthesized before *GTP *was totally consumed. In Figure 4(C), the substrates and products of the ligation reactions are shown. In the left-hand panel, the rate of the second ligation reaction was fast relative to the first ligation reaction and all of the intermediate product was converted to final product at the end of the simulation. In the right-hand panel, the opposite was true, and much less intermediate product was converted to final product. The difference in rates can be traced to the amount of *Pro_A *and *Pro_B *formed (Figure 4(D)). Here, the total protein translated is plotted using the fictitious state variable, Pro_A* (*t250*) and Pro_B* (*t251*) that were included in the model code to count every translation event for *geneA *and *geneB*, respectively. In the left-hand simulation more *Pro_B *than *Pro_A *was formed and vice versa in the right-hand simulation. Thus, by inspecting several individual simulations some insight into the possible range of dynamical behavior of the system can be obtained.

Figure 4(D) also illustrates another feature of the stochastic nature of the system. The two genes, *geneA *and *geneB*, are treated identically in this exemplar model in the sense that their nucleotide compositions are the same and all reactions with the RNA polymerase, subsequent transcription reactions, ribosomal subunits and translation reactions have the same probabilistic reaction constants. Therefore, if the system was continuous and deterministic the two genes would be expressed at the same rate and attain the same magnitude. However, due to the stochastic nature of the system, neither the rates nor the magnitudes of synthesis of *Pro_A *and *Pro_B *are identical within a given simulation. Furthermore, the rates and magnitudes are not repeatable from simulation to simulation. Inspecting Figure 4(D) reveals that in two cases more *Pro_A *was translated than *Pro_B *and in one more *Pro_B*. In the simulations shown in Figure 4(D), the protein that started with the higher translation rate ended up making the most protein. However, if more simulations are investigated, it is found that this is not always true, i.e., the two trajectories can cross over. Note also that for these state variables, both of which monotonically increase with no fluctuations, there is significant variability between simulations in the total protein synthesized at the end of the simulation ranging from 40 – 75 protein molecules synthesized.

**Figure 5:**
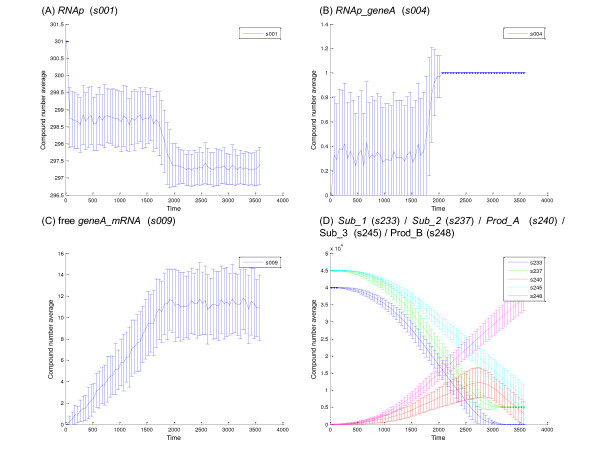
**Estimates of the mean ± standard deviation of the probability density function *P*(***s***, *t*/***s***_**0**_, *t*_0_) for selected state variables**. These plots were obtained using the snapshot data (SSI = 50 sec) from 100 simulation runs. The *x*-axis is time (sec) and *y*-axis is the number of molecules of each state variables. These plots were obtained from the 'Time-Weighted Analysis' Tool in the BNS Toolbox.

As a consequence of the inherent stochastic variability of the behavior of state variables, experimental time-series data obtained from individual biomolecular reaction systems (vesicles in this example) represent single physical instances of the system under investigation. In general, one would conduct multiple experiments on individual systems and compute the mean and standard deviation of the observed time series (see discussion below). However, if one wished to investigate the behavior of a single experimental time series in relation to the model predictions, the only meaningful comparison is between the experimental data for the selected state variable *s*_*i *_and the simulation ensemble mean ± standard deviation for *s*_*i*_. This is because the simulation ensemble mean and standard deviation are the best estimates of the mean (first moment) and standard deviation (square root of the second moment) of the state density function *P*(***s***, *t*/***s***_**0**_, *t*_0_) for each selected state variable *s*_*i *_(Equations 3 and 5). Figure 5 shows the mean ± the standard deviation of selected state variables using the snapshot data from 100 simulations. These plots were created using the 'Time-Weighted Average' Tool in the BNS Toolbox. This tool was set-up to calculate the mean and standard deviation of the number of molecules of the selected state variable for the ensemble of 100 simulations at 50 sec intervals. These plots are estimates of the temporal behavior of *P*(***s***, *t*/***s***_**0**_, *t*_0_) based on the model. If the model is a reasonable representation of the physical system, two thirds of the time the experimental data for a single vesicle should fall within the envelop of the mean ± the standard deviation. However, significant excursions from the envelop can occur even when the model is a correct representation of the experimental system and thus observing the behavior of a single experimental time series may be misleading. As suggested above, a better comparison between experimental data for single systems and model simulations is between the experimental mean ± standard deviation obtained from multiple (many) single vesicle observations versus the mean ± the standard deviation of an ensemble of a large number of simulations runs (see discussion below on the effect of the number of simulation runs on estimates of the mean and standard deviations of the probability density function for the system).

As the number of individual experiments on single systems increases, the experimental estimates of the mean and standard deviation of the system state variables improve. However, if experimental data for state variables are only obtained as the mean of a large composite sample of vesicles (i.e., the data are obtained by analyzing samples consisting of a large number of individual systems), then the only meaningful comparison is between this 'macro-sample' mean and the mean of a large number of simulations at corresponding time points. In this case, no data concerning the variability between individual vesicles can be obtained. Note, the standard deviation obtained from analyzing multiple macro-samples does not correspond to the fluctuations exhibited in individual model simulations of the system or fluctuations between model simulations, but rather is a measure of experimental uncertainties (e.g., experimental measurement errors and non-identical systems), which are not taken into consideration by the model simulations. In fact, if there were no experimental uncertainties, then the macro-means of multiple macro-samples taken from ensembles of identical systems would converge to a single value as the number of individual systems collected in the macro-sample increases.

**Figure 6:**
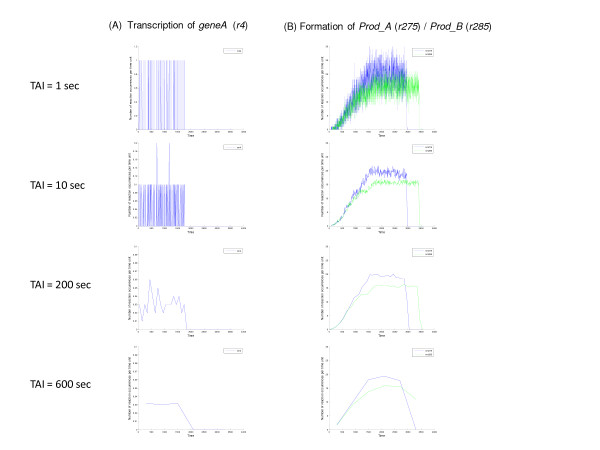
**Effect of time-averaging interval (TAI) on time averaged reaction event rates**. The time-averaged event rate data for a single simulation were calculated using various TAIs from 1 to 600 sec for selected reactions. The same random number seed was used for each set of simulations (rnseed = 100). The *x*-axis is time (sec) and the *y*-axis is the reaction event rate (number of reaction events per sec). Note the difference in scale between the TAI = 1 and 10 sec panels and the other panels in Figure 6(A). These plots were generated by the 'Reaction Frequency' Tool in the BNS Toolbox.

To further investigate the behavior of the system, the event rates of selected reactions were investigated. As the system is treated as a discrete jump Markov process, each event occurs instantaneously and the value of associated state variables change discontinuously at the time of the event. Consequently, there is no derivative of the state variables that would correspond to the C-D concept of rate of change. Hence, for these processes, the 'reaction rate' is defined as the number of events counted during a time-averaging interval (TAI) divided by that time interval, giving an estimate of the reaction event rate (number of events per unit time). These estimates will depend on the TAI as illustrated in Figure 6. When calculating the reaction event rate in a particular reaction channel, a very small TAI relative to the average time between events results in counting individual events, depending on whether an event occurs or not in a particular time averaging interval. Subsequently dividing by a small TAI results in what appears as an anomalously high reaction event rate and the computed reaction event rate will exhibit large fluctuations between successive sample times depending on whether an event occurred or not. This effect is most obvious in the TAI = 1 sec panel in Figure 6(A), where the between sample time variability is large fluctuating between 1 and 0 (in Figure 6(A) note the difference in scales in the TAI = 1 and 10 sec plots compared to the other plots). On the other hand, a large time-averaging interval will reduce the variability thus smoothing the data, but will affect the time resolution and the ability to precisely observe dynamical changes in rates due to the averaging over long intervals. For the results discussed below, a TAI of 50 sec was selected to maximize time resolution of system dynamics without significant artifacts due to too small a TAI.

**Figure 7:**
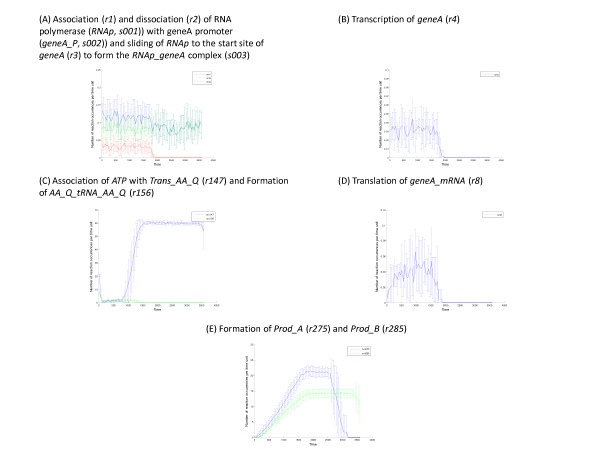
**Time-averaged reaction event rates of selected reactions**. Each panel shows the mean ± SD for the time-averaged reaction event rates (time averaged over 50 sec intervals) averaged over an ensemble of 10 simulation runs. The *x*-axis is the time (sec) and the *y*-axis is the reaction event rate (number of events per sec). These plots were constructed with the 'Reaction Frequency Analysis' Tool in the BNS Toolbox.

The total number of reaction events in each reaction channel during the entire simulation run is calculated by the BNS software. fIn this exemplar model, the total number of reaction events in a given reaction channel ranged from one to several hundred thousand over the 3600 sec simulation. The specific reaction event rate was computed for selected reactions using a user defined TAI of 50 sec as discussed above and the results are shown in Figure 7. In Figures 7(A) through 7(E), the mean ± one standard deviation for the ensemble of 10 simulations are shown. For the selected reactions, the reaction event rates vary during the simulation depending on the availability of substrates (and enzymes where required). For example, the reaction event rate ranged from 0 – 0.06 reaction events per sec for transcription (reaction *r4*, Figure 7(B)), 0 – 25 reaction events per sec for formation of metabolic products *Prod_A *and *Prod_B *(reactions *r275 *and *r285*, Figure 7(E)) and 0 – 60 reactions events per sec for the association of *ATP *with the amino acyl-transferase for glutamine (reaction *r147*, Figure 7(C)). By plotting the event rates for several related reactions on the same graph, it is possible to observe relationships between reactions. For example, Figure 7(A) plots the association and dissociation of the RNA polymerase (*RNAp*, *s001*) with the promoter for geneA (*geneA_P*, *s002*) along with the sliding reaction of the bound polymerase to the start site to form the start complex (*RNAp_geneA*, *s003*). As long as transcription is proceeding, the association rate (reaction r1) exceeds the dissociation rate (reaction r2) and newly bound polymerase continues to slide to the start site (reaction r3) and transcription of geneA_mRNA proceeds (reaction r4 shown in Figure 7(B)). However, when *GTP *is depleted (t > 1700 sec), transcription cannot proceed and the polymerase stalls at the start site blocking the sliding of new polymerase molecules to the start site (reaction *r3*). The polymerase sliding reaction ceases and now the association and dissociation reactions are equal resulting in a new quasi-steady state for the RNA polymerase (see Figure 5(A)).

A unique feature of stochastic systems is that the timing of specific events varies from one simulation to the next. An example of this effect is seen in Table 1, where the time of the last transcription event is displayed for the transcription of geneA (reaction *r4*) and translation of *geneA*_mRNA (reaction *r8*) into *Pro_A *for each of the 10 simulation runs. These data were obtained from the parsed event log data files viewed in the MATLAB workspace. The transcription reaction terminates when the available *GTP *is depleted and ranges from 1637 to 1907 sec with a mean and standard deviation of 1757 ± 77 sec for the transcription of *geneA*. The translation reaction stops when *GTP *and/or the limiting amino acid charged tRNA for glutamine (*AA_Q_tRNA_AA_Q*, *s105*) is depleted which occurs over a range of 1354 to 1870 sec with a mean and standard deviation of 1653 ± 147 sec for the transcription of geneA. Thus, the timing of any specific event in a stochastic process will always appear as a distribution rather than a fixed time as would be the case for a C-D process.

**Table 1:** Time of last transcription and translation events.

Simulation Run	Time of Last Transcription Event for geneA (*r4*)	Time of Last Translation Event for geneA (*r8*)
1	1757	1592
2	1700	1634
3	1907	1870
4	1693	1631
5	1758	1739
6	1822	1504
7	1703	1710
8	1637	1354
9	1781	1727
10	1805	1774
		
Average	1757	1653
Standard Deviation	77	147

For each of the 10 simulation runs, the time of the last transcription event for transcription of geneA (reaction *r4*) and translation event for geneA (reaction *r8*) is given. These data were obtained from the parsed event log.

### Improvement in estimating the mean and standard deviation of s with the number of simulation runs

**Figure 8:**
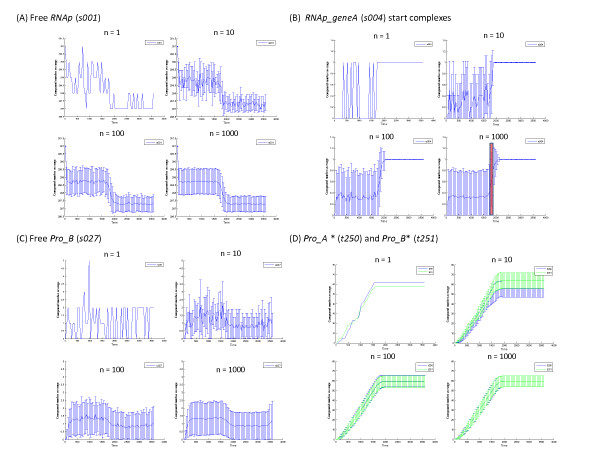
**Comparison of estimates of the mean and standard deviation of selected state variables with increasing numbers of simulation runs**. For each state variable, the mean ± SD for the number of molecules are plotted using a SSI = 50 sec for various numbers of simulations. The *x*-axis is time (sec) and the *y*-axis is the number of molecules of the given state variables. These plots were obtained from the 'Time-Weighted Analysis' Tool in the BNS Toolbox.

The mean and standard deviation of the number of molecules for the ensemble of simulation runs at a given time *t *allow one to estimate the first and second moments of the probability density function, *P*(*s*, *t*/*s*_0_, *t*_0_), for the random variable ***s ***as defined by the solution of the CME (see Equations (3) – (5)). As the number of simulations increases, the accuracy of these estimates improve. This can be seen by inspecting the estimated mean ± SD for batch jobs with increasing numbers of simulation runs (Figure 8). Figure 8(A) and 8(B) illustrate the behavior of two state variables that can only fluctuate between a limited number of states due to the stoichiometry of the system. Figure 8(A) shows the free RNA polymerase (*RNAp*, *s001*) where, although the number of total molecules is fairly large (301 in this exemplar model), the state variable can only fluctuate between 301 and 297 molecules corresponding to zero or four polymerase molecules bound to the promoter and start sites of the two genes. The mean and SD for an ensemble of simulation runs fluctuates significantly from one sample time to the next when averaged over a small number of simulation runs – i.e., the mean appears to be noisy when the number of simulations are small (Figure 8(A), n = 10 plot). However, this is merely a consequence of the approximate nature of the statistical estimate of the first and second moments of the solution of the CME using a small number of simulations. In fact, the exact mean, ⟨*s*(*t*)⟩, and the SD, ⟨*σ*(*t*)⟩, are smooth functions of time as the series of approximations with increasing *n *in Figure 8(A) suggests. Only for estimations of the mean with *n *≥ 100 runs does the mean become a relatively smooth function of time. Also, the shift in the mean between 1600 and 2000 sec becomes well defined with increasing number of simulations. This shift is due to the stalling of two RNA polymerase molecules at the start sites of *geneA *and *geneB *when *GTP *is depleted resulting in the cessation of mRNA synthesis. Figure 8(B) shows the behavior of the estimates of the mean and SD of the probability density function for the complex of the RNA polymerase and the start site for *geneA *(*RNAp_geneA*, *s004*). For this state variable, the only possible states in state space are either 0 or 1 corresponding to when the start site is unoccupied and when the polymerase is bound at the start site, respectively. Thus, the number of molecules of the complex fluctuate over time between 0 and 1 in any given simulation of the system (Figure 8(B), n = 1 plot). This rapid fluctuation continues until transcription terminates due to the depletion of *GTP*. The red band in the n = 1000 plot indicates the mean ± one SD for time when transcription terminated as calculated in Table 1. When mRNA transcription terminates, a polymerase is locked at the start site and the value of the state variable RNAp_geneA (s004) becomes a constant. Also, these data indicate that at any given time during active transcription, the start site for *geneA *would be occupied by a polymerase in approximately 30 percent of the individual systems in an ensemble of a large number of systems.

Figure 8(C) shows the behavior of free protein B (*Pro_B*, *s027*) as *n *increases. In this case, the values of the state variable is not inherently limited by the stoichiometry of the system. For *Pro_B*, there are approximately 60 molecules synthesized. However, because *Pro_B *forms a tetramer and that tetramer is involved in catalytic reactions, the number of free molecules of *Pro_B *remains low throughout the simulation and fluctuates rapidly due to the association and dissociation of the dimer – tetramer complexes. Again, the estimates of the ensemble mean and SD show significant fluctuations from one time point to the next when *n *is small due to the inaccuracies in each estimate of ⟨*s*(*t*)⟩_*n *_and ⟨*σ*(*t*)⟩_*n*_. As *n *increases, each individual estimate of the mean of ***s***(*t*) and SD improves and the plot approaches the exact smooth curve for *P*(*s*, *t*/*s*_0_, *t*_0_). For this state variable, the details of its behavior only become well defined with 100 or more simulations.

Finally, Figure 8(D) shows the time course of the number of proteins translated using the fictitious variables *t250 *and *t251 *to count the number of molecules of *Pro_A *and *Pro_B *synthesized, respectively. For any individual simulation, the translation of *geneA *and *geneB *can be quite independent within the constraint that the total number of protein molecules synthesized are limited by the availability of energy and substrates (Figure 4(D)). However, as the number of simulations increases, the ensemble means of Pro_A* and Pro_B* approach each other demonstrating that the expression of the two genes are controlled in an identical manner. All of these examples emphasize the need to conduct a large number of simulations to obtain accurate estimations of the behavior of the state variables.

The dependency of the accuracy of the estimates of the mean and SD of state variables on the number of simulations is an issue that must be taken into consideration when comparing model predictions with experimental data. If simulations are used to estimate what the model would predict for experimental observations, then the values for state variables predicted by the model will only be exact in the limit of n → ∞ simulations. Therefore, if a finite number of simulations are used, there will be some error in the model predictions. There are several situations where this issue becomes particularly important. One example is when one believes that a model is adequate, but there are still minor discrepancies between model predictions and the available experimental data. The usual cause for this situation is that the uncertainty in some model parameters, such as the reaction probability constants, leads to uncertainties in simulations that are greater than the uncertainties in the experimental data. To investigate whether minor adjustments to model parameters will improve the correlation between model predictions and experimental data, various optimization techniques can be employed. To successfully adjust model parameters based on experimental data, it is necessary to compute a large number of simulations to adequately estimate the behavior of the system each time model parameters are variedin the optimization algorithm. If too small a number of simulations are used, the fluctuations in model predictions as the model parameters are varied from one cycle to the next of the optimization process will be so great as to limit the usefulness of the optimization procedure. The larger the number of simulations the better the estimate of the model prediction, thus reducing this additional source of error that is not present when fitting solutions of C-D ODEs to experimental data.

**Figure 9:**
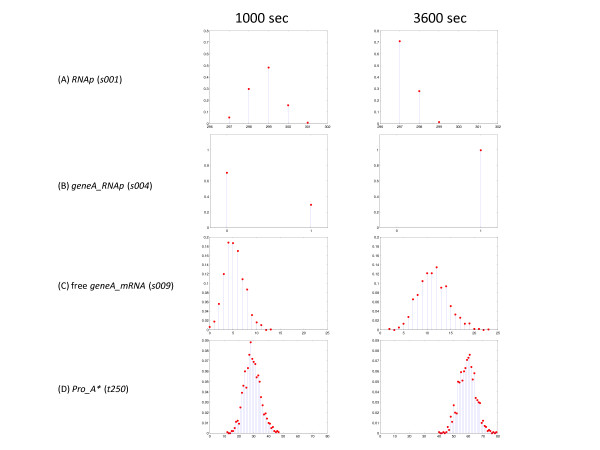
**Histograms of the distribution of state variables at two times**. Histograms of the selected state variables were computed at *t *= 1000 and 3600 sec using the data from 1000 simulations. The left hand panels are the data at *t *= 1000 sec while the right hand panel is at *t *= 3600 sec. The *x*-axis is the number of molecules of each state variable and the *y*-axis is the probability of occupancy. These histograms were generated from data files generated by the 'Time Weighted Average' Tool in the BNS Toolbox.

Similar issues arise when investigating reaction event rates. Averaging over multiple runs gives a more accurate and consistent estimate of the model predictions of the mean and SD of the reaction event rate as a function to time. It should be noted that the mean and SD for the time-averaged reaction event rate are not directly related to the first and second moments of the state density function *P*(*s*, *t*|*s*_0_, *t*_0_), but are related to the reaction probability constants. The reciprocal of the time-averaged reaction event rate is the mean time between reactions given the current state of the system, which is determined by the propensity. Thus, the time-averaged reaction event rate is related to the reaction probability constants through the state dependent propensity. Even for reactions that occurs at a significantly greater rate than transcription or translation, e.g., the metabolic formation of products *Prod_A *and *Prod_B *(Figure 9(B)) with a reaction event rate ranging from 0 to 23 events per sec, the effect of averaging over multiple simulations is still apparent, particularly for the details of the mean and standard deviation.

When sufficiently large numbers of simulations are performed, it becomes reasonable to generate discrete histograms of the distributions of state variables at defined times. Figure 9 shows the distribution of several state variables at two time points, one during active transcription and translation (*t *= 1000 sec) and the other at the end of the simulation (*t *= 3600 sec). These histograms were generated using 1000 individual simulation. This is an estimate of the probability that the system would be in that particular state at the defined time *t*, i.e., the state probability density *P*(*s*, *t*|*s*_0_, *t*_0_). During gene transcription and translation (*t *= 1000 sec), the free polymerase probability density (Figure 9(A)) is distributed among the five possible states from no polymerases bound (*s001 *= 301) to four polymerases bound (*s001 *= 297) with two polymerases bound (*s001 *= 299) being the most probable state. At the end of the simulation (*t *= 3600), the most probable state is with four polymerases bound (prob = 0.7) and furthermore, the states with zero or one polymerase bound (*s001 *= 300 or 301) are not populated (prob = 0). This is due to the fact that when transcription terminates, two polymerases are stalled at the start sites of the two genes. This is seen in Figure 8(B), where before transcription terminates (*t *= 1000 sec), the start site of gene A is only occupied with a probability of approximately 0.3 while after transcription terminates it is occupied with a probability of 1.0. Figure 9(C) and 9(D) are the estimates of the state probability density functions for the free gene A mRNA (*geneA_mRNA*, *s009*) and the total number of protein A molecules synthesized (Pro_A*, *t250*). Both of these state variables increase with time while transcription and translation are active. Furthermore, the number of possible states available to each state variable increases and is only limited by the total number of molecules of mRNA and protein A synthesized. The free *geneA *mRNA state variable is influenced by five reactions (its synthesis (*r4*), its degradation (*r5*), its association and dissociation with the small ribosomal unit (*r6 *and *r7*), and its release after translation (*r8*)), while the fictitious total protein synthesized variable is only a counter for the number of transcription reactions (*r8*). The variability in Pro_A* is a consequence of the stochasticity in the transcription reaction alone, while the variability in *geneA_mRNA *is a combination of the variability in the total number of mRNA molecules transcribed at a given time and the variability introduced by the other reactions that influence that state variable. Discrete histograms of the state variables give a better visualization of the true nature of the state probability density function than just the first and second moments.

### Comparison between single and multi-processor simulation runs

As is apparent from the discussion above, the ability to accurately estimate the first and second moments of the state density function of the system under investigation using the Monte Carlo simulation approach increases with increasing numbers of simulations. Various analysis techniques, such as optimization (data fitting) and sensitivity analysis, require repeated batch jobs of large numbers of simulation to obtain statistically valid results. This may not be a problem for a relatively simple biomolecular reaction system, but as the complexity of the system model increases this will increase the computational demand. The need for high performance computing becomes essential.

Running a simulation session as a batch job on multi-processor HPC hardware entails a certain amount of overhead, e.g., the time it takes to break up the job into smaller tasks and assign the problem to each processor on the front end and the data collection and storage on the back end. As a result, the speed-up gained by using multi-processor hardware is to a degree dependent on how computationally intensive the problem is. For a relatively simple problem that is not particularly computationally intensive, the majority of the clock time for the simulation session is taken up with overhead. Whereas, for a problem that is computationally intensive, the computations involved in the actual simulation are the time consuming component of the simulation process.

**Figure 10:**
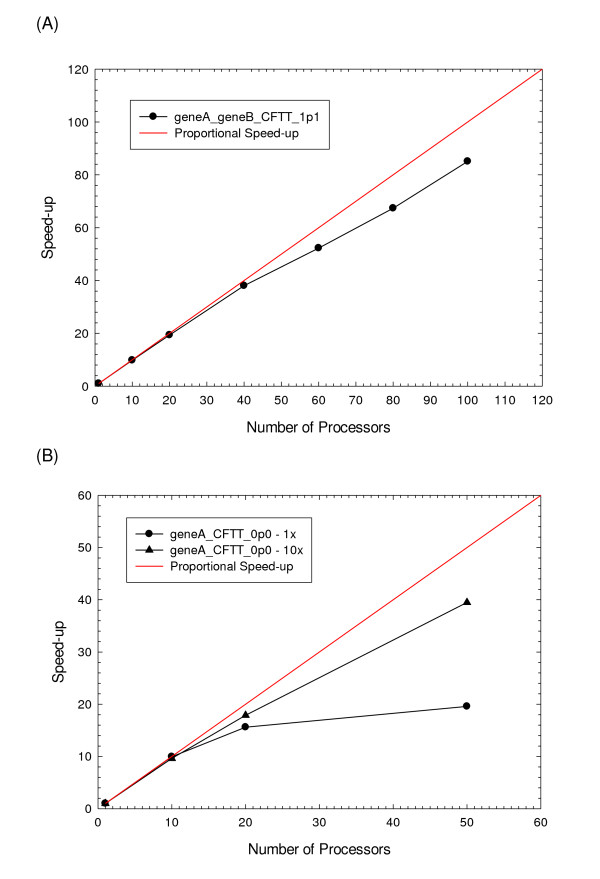
**Scaling of run time with the number of processors**. Speed-up was calculated as the run time for the batch job on one processor divided by he run time with the given number of processors. (A) Speed-up for the two gene (geneA_geneB_CFTT_1p1) exemplar model. (B) Speed-up for the one gene (geneA_CFTT_0p0) model with 1× and 10× parameter sets. All models were run 10,000 times as a batch job using the BNS software on a HP XC machine with distributed memory architecture using up to 100 processors.

To investigate this effect, we ran a batch job with the exemplar model using multi-processor HPC hardware to evaluate the speed-up in clock time with increasing numbers of processors. Specifically, we executed 10,000 simulation runs of the exemplar model as a batch job on an HP XC machine with distributed memory architecture using the Gillespie direct stochastic simulation algorithm and various numbers of processors. The speed-up, defined as the clock time for a run with one processor divided by the clock time for the same run on multiple processors, is shown in Figure 10(A). For a simulation of this computational complexity (clock time for the batch job on one processor was 41,351 sec), the speed-up was relatively linear with the number of processors. However, the speedup observed by running the model on 100 processors in the batch mode was only 85.1-fold. This drop-off in performance is due to the computational overhead discussed above, and thus, the performance when using more than 100 processors results in diminishing returns

To further explore this effect, we repeated the test with a batch job of significantly less computational complexity. Using a one gene model with lumped reactions (denoted geneA_CFTT_0p0 – 1× model with a total of 45 state variables and 12 reactions), the speed-up for a batch job consisting of 10,000 simulation runs was calculated using up to 50 processors. The clock time for a single processor to run the batch job was 1,920 sec and the speed-up is shown in Figure 10(B). In this case, the computational demand for the actual simulations is relatively small and the overhead becomes a dominant factor. Given these conditions, using more than 20 processors provides little benefit. The computational complexity of this relatively simple model can be increased by increasing the number of plasmids per reaction network from 1 to 10 and the number of substrate molecules by a factor of 10 (denoted the geneA_CFTT_0p0 – 10× model). This is equivalent to having 10 plasmids containing geneA present in the same reaction volume with ten times the number of substrate molecules available. The speed-up results using the 10× model are also given in Figure 10(B). Here the value of additional processors is clearly apparent even when 50 processors are accessed.

## Conclusion

In this manuscript, we have tried to point out some of the issues that arise in interpreting the results of modeling and simulating discrete stochastic systems. The issues of how the snapshot interval affects the visualization of state variables and how the time-averaging interval affects the estimation of the time-averaged reaction event rates illustrate how simulation and analysis parameters can influence the interpretation of system behavior. Probably even more important is how the number of simulations affect the estimation of the mean and SD of state variables. Unless sufficient numbers of simulations are conducted, these estimates will not be adequate to: (1) observe the details of the temporal dynamics of state variables, (2) accurately estimate the variability between simulations, or (3) when optimization procedures are being employed, to expect consistent convergence of solutions.

An important point to remember is that the stochastic nature of individual state variables is to some extent model dependent, i.e., will depend on: (1) the relationships between state variables, (2) the mathematical forms of the reaction propensities, (3) the values for reaction probability constants, and (4) initial conditions for state variables. For example, if the diameter of the vesicle was increased 10 times (from 1.0 to 10.0 *μ*m), then the initial numbers of molecules of each state variable would be increased 125 times (except for the number of genes as determined by the number of gene promoter sites which would still be 2, one for each gene). In this case, the effect of stochastic processes on some state variables, such as the transfer RNAs, would be significantly diminished while the effect on others, i.e., transcription, would remain.

In addition, we investigated the generic behavior of a biomolecular reaction network consisting of the expression of two genes in a cell-free transcription-translation system enclosed in a artificial reaction vessel. Such a closed system does not reach a steady state and generates a different class of kinetics than is found in systems where it is assumed there is an infinite supply of substrates and energy and all waste products are 'taken care of' by the system. The closed system investigated here simply 'dies out' when critical components are exhausted. The ability to simulate such a system allows one to identify the critical factors that limit the performance of the system. Although the model for the system is still relatively crude, it is clear that the availability of limiting amino acids controls the ultimate expression of proteins, the availability of GTP limits transcription of the plasmid genes to form mRNA and the availability of substrates (including ATP) for the catalytic ligation reactions limits the generation of the final products. This quantitative knowledge can be used, for example, to optimize the system to maximize production of products, either proteins or metabolites. As the models for the CFTT-vesicle system become more sophisticated, a more detailed understanding of the behavior of these biological constructs will evolve.

## Availability and requirements

The BNS software is available to all researchers through the website below.

**Project name: **Biomolecular Network Simulator

**Project home page: **

**Project downloads: **

**License: **GNU GPL

**Operating systems: **Platform independent

**Programming Language: **C, MATLAB

**Other Requirements: **MATLAB 6.5 or newer; MatlabMPI and MPI libraries for multiprocessor execution.

## Authors' contributions

JMF developed the two gene model, ran simulations, prepared graphics and drafted the manuscript. BF developed the original BNS code, developed and evaluated the mathematical analysis tools and helped draft the manuscript. YC modified the original BNS code to increase efficiency, ported the software to run on multiprocessor hardware and reviewed the manuscript. All authors approved the final manuscript.

## Supplementary Material

Additional File 1 **Model Description**. The information provided describes the conceptual structure of the exemplar model and the mathematical description of the model reactions. Click here for file

Additional File 2 **SBML Model Definition**. This file contains the SBML model definition code for the exemplar model. Click here for file
