# Supplementary material for: Stochastic simulation and analysis of biomolecular reaction networks
Source: BMC Syst Biol. 2009 Jun 17;3:64. doi: 10.1186/1752-0509-3-64 (PMC2708125; doi:10.1186/1752-0509-3-64)

stochastic simulation and Analysis of

BiomoleculAr Reaction Networks

Additional File 1 - Model Description

John Frazier

Air Force Research Laboratory

WPAFB, OH 45433-5707

Yaroslav Chushak

Biotechnology HPC Software Applications Institute

US Army Medical Research and Materiel Command

ARLF/RHPB WPAFB, OH 45433-5707

Brent Foy

Department of Physics

Wright State University

Dayton, OH 45435

**Schematic Diagram of Conceptual Model**

To formulate a relatively simple, yet biochemically reasonable, model of the kinetics of the self-assembly of the examplar biomolecular reaction network and the subsequent metabolic reactions, the conceptual system model illustrated in Figure S1 was proposed. The diagram in Figure S1 is a schematic of the various reactions and material flow connections between state variables of the two gene biomolecular reaction network model (referred to as the geneA_geneB_CFTT_1p1 model). This model consists of 249 state variables and 287 reactions. This is the simplest model for the biomolecular reaction network that retains the basic features of the system. As a consequence of the fact that the transcription and translation reactions are described at the conceptual level as lumped macro-reactions, this model represents an approximation to the exact fundamental representation of the biomolecular reaction network that would be rigorously compliant with the basic tenants of the Markov process theory of multi-variant, discrete state, temporally homogenous, Markov jump processes.

To transform the conceptual model into a schematic more representative of the mathematical description of the system, the model schematic in Figure S1 can be redrawn as in Figure S2 where a semi-descriptive nomenclature is used to identify state variables. In this representation, the nature of each reaction is defined and all of the substrates for each reaction are easily identified. The semi-descriptive nomenclature is converted to an mathematical nomenclature (*s001* - *s249*) to simplify the code for the model (Figure S3).

**Reactions for a Minimal Model of the Two gene Self-assembly Biomolecular Reaction Network**

The reactions in this model are separated into 4 categories: (1) transcription, (2) translation, (3) metabolic reactions, and (4) degradation reactions. The details of the reactions that fall into each of these categories are given below.

**Transcription (Reactions *r1* - *r4* and *r9* - *r12*)**

In this simple model, the transcription of *geneA* and *geneB* in the cell-free transcription-translation (CFTT) expression system is treated as a three step reaction: (1) reversible association and dissociation of the RNA polymerase (*RNAp* - *s001*) and the promoter sites of the two genes on the plasmid (*P_A* - *s002* and *P_B* - *s022*) to form the polymerase-promoter complexs (*RNAp_P_A* - *s003* and *RNAp_P_B* - *s024*), (2) sliding of the polymerase to the gene start sites (*RNAp_geneA* - *s004* and *RNAp_geneB* - *s024*) and (3) creation of the mRNAs (*geneA_mRNA* - *s009* and *geneB_mRNA* - *s025*) in an instantaneous event at a time determined by the stochastic probability. The formation of the mRNAs utilizes the appropriate number of nucleotide triphosphates (NTPs: *ATP - s005*, *GTP - s006*, *CTP - s007* and *UTP - s008*) and creates one pyrophosphate molecule (*PPi - s010*) as byproduct per NTP incorporated. The number of each NTP used is determined by the sequence of *geneA* and *geneB*, which are considered to be identical in this model, and the number of *PPi* generated for each molecule of mRNA formed will be equal to the total number of nucleotides in the translated portion of *geneA* and *geneB*.

The specific reactions involved in transcription are:

Reaction *r1*and *r9*: The initial step in transcription of the *geneA* and *geneB* is the association of RNA polymerase with its promoter sites on the plasmid containing the genes.

*RNAp* + *P_A* -> *RNAp_P_A* (*r1*)

*RNAp* + *P_B* -> *RNAp_P_B* (*r9*)

Reactions *r1* and *r9* are fundamental stochastic reactions that are described by standard Markov propensities for bi-substrate reactions:

*a_1_ = c_1_*s001*s002/V_R_*

*a_9_ = c_9_*s001*s022/V_R_*

where the semi-descriptive names for the state variables have been replaced with the names used in the model code.

Reaction *r2* and *r10*: Dissociation of the polymerase-promoter complex.

*RNAp_P_A -> RNAp + P_A*  (*r2*)

*RNAp_P_B -> RNAp + P_B* (*r10*)

Reactions *r2* and *r10* are fundamental stochastic reactions that are described by standard Markov propensities for uni- substrate reactions:

*a_2_ = c_2_*s003*

*a_10_ = c_10_*s023*

Note, there is only one plasmid in the reaction volume, therefore, the state variables *P_A* (*s002*) and *P_B* (*s022*) that represents the promoters on the plasmid for the two genes can fluctuate only between values of 1 and 0, where *P_A* or *P_B* = 1 implies the promoter site is unoccupied and *P_A* or *P_B* = 0 when *RNAp* is associated with the promoter site. Thus, the propensities for the association and dissociation reactions are:

$$a_{1}=\left\{\begin{matrix} c_{1}*s001/V_{R} when s002=1 \\ 0 when s002=0 \end{matrix}\right.$$

and

$$a_{2}=\left\{\begin{matrix} c_{2} when s003=1 \\ 0 when s003=0 \end{matrix}\right.$$

Reaction *r3* and *r11*: When the promoter site of *geneA* or *geneB* is occupied by the polymerase, it is possible for the polymerase to slide to the start site of the given gene to form the start complex, *RNAp_geneA* (*s003*) or *RNAp_geneB* (*s023*). This sliding reaction is treated as an irreversible process:

*RNAp_P_A -> RNAp_geneA + P_A* (*r3*)

*RNAp_P_B -> RNAp_geneB + P_B* (*r11*)

Reactions *r3* and *r11* are fundamental stochastic reactions that are described by standard Markov propensities for uni- substrate reactions. However, the sliding reaction can only occur when the start site is unoccupied. Thus:

*a_3_ = c_3_*s003*(1 - s004)*

and

*a_11_ = c_11_*s023*(1 - s024)*

where the additional term on the right-hand side assures that the reaction will not occur if the start site is occupied. Again note, there is only one plasmid in the reaction volume, therefore the propensities for the physical sliding reactions are:

$$a_{3}=\left\{\begin{matrix} c_{3} when s003=1 and s004=0 \\ 0 when s003=0 or s004=1 \end{matrix}\right.$$

and similarly

$$a_{11}=\left\{\begin{matrix} c_{11} when s023=1 and s024=0 \\ 0 when s023=0 or s024=1 \end{matrix}\right.$$

Reaction *r4* and *r12*: Elongation and ultimate formation of the *geneA_mRNA* and *geneB_mRNA* polymers proceeds by the incorporation of the nucleotide tri-phosphates (*UTP*, *CTP*, *ATP* and *GTP*) into the RNA polymers. Inorganic pyrophosphate (*PPi*) is formed as a byproduct.

*RNAp_geneA* + 381 *UTP* + 429 *CTP* + 377 *ATP* + 369 *GTP* ->

*geneA_mRNA* + *RNAp* + 1556 *PPi* (*r4*)

*RNAp_geneA* + 381 *UTP* + 429 *CTP* + 377 *ATP* + 369 *GTP* ->

*geneA_mRNA* + *RNAp* + 1556 *PPi* (*r12*)

where the nucleotide composition of a generic *geneA* and an identical *geneB* has been used. Reaction *r4* is a lumped, macro-reaction and must be treated as an approximation to the exact series of fundamental reactions that constitute the transcription reaction. Again, as the consequence of there being only one plasmid in the reaction volume, when *RNAp* is associated with a start site, then *RNAp_geneA* (*s004*) = 1 or *RNAp_geneB* (*s024*) = 1, and the transcription reactions (*r4* and *r12*) can occur. The propensities for these reactions are:

*a_4_ = c_4_*RNAp_geneA*f_4_(ATP, GTP, CTP, UTP)/V_R_*

*= c_4_*s004*f_4_(s005, s006, s007, s008)/V_R_*

and

*a_12_ = c_12_*RNAp_geneB*f_12_(ATP, GTP, CTP, UTP)/V_R_*

*= c_12_*s024*f_12_(s005, s006, s007, s008)/V_R_*

where *f_3_(ATP, GTP, CTP, UTP)* and *f_12_(ATP, GTP, CTP, UTP)* describe the dependency of the polymerization reactions on the NTP substrates. Note, reaction *r4* and *r12* can take on only two values

$$a_{4}=\left\{\begin{matrix} 0 when s004=0 \\ c_{4}*f_{4}(s005,s006,s007,s008)/V_{R} when s004=1 \end{matrix}\right.$$

Obviously, a detailed model of the sequence of micro-reactions that constitute the overall transcription reaction would be more a more exact representation of the process and the natural dependency of the reaction on the availability of substrates would appear as a consequence of the fundamental reactions involved. However, for the purposes of this simple model, we will treat *r4* and *r12* as lumped macro-reactions and use an approximate phenomenological formulation for the propensity based on the Michaelis-Menten approximation for isomerization reactions. Thus,

$$\begin{matrix} f_{4}(ATP,GTP,CTP,UTP)=\left(\frac{ATP}{ATP+km3\_ATP}\right)*\left(\frac{GTP}{GTP+km3\_GTP}\right)* \\ \left(\frac{CTP}{CTP+km3\_CTP}\right)*\left(\frac{UTP}{UTP+km3\_UTP}\right) \end{matrix}$$

$$\begin{matrix} \\ =\left(\frac{s005}{s005+km4\_s005}\right)*\left(\frac{s006}{s006+km4\_s006}\right)* \\ \left(\frac{s007}{s007+km4\_s007}\right)*\left(\frac{s008}{s008+km4\_s008}\right) \end{matrix}$$

Note, 0 ≤ *f_4_(s004,s005,s006,s007)* ≤ 1, thus this formulation guarantees that the propensity will be zero if any one of the substrates is depleted, and will be limited to a maximum value of *c_4_* even when there is excess of the NTP substrates. A similar relationship holds for reaction *r12*.

The probability that transcription will occur is non-zero as long as the polymerase is bound to the start site and there are sufficient NTP substrates available to form a complete mRNA polymer. The BNS simulation algorithm checks each lumped reaction to be sure there are sufficient substrates to complete the reaction. If not, the propensity is set to zero. As transcription progresses, the *geneA_mRNA* (*s009*) and *geneB_mRNA* (*s025*) formed serve as the substrates for either the translation process or for the mRNA degradative process.

**Translation (Reactions *r6*-*r8* and *r14*-*r16*)**

As with the transcription process, in this simplest conceptual model (geneA_geneB_CFTT_1p1), the translation process is treated as a two step process: (1) reversible association-dissociation of the ribosomal small unit (*Rib_s*, *s015*) to the ribosomal binding site (RBS) on *geneA_mRNA* (*s009*) or *geneB_mRNA* (*s025*), and (2) translation of the mRNA into protein (*Pro_A* or *Pro_B*) by an instantaneous event at a time determined by the stochastic probability.

The three translation reactions are:

Reaction *r6* and *r14*: Association of the ribosomal small-unit (*Rib_s* - 30S subunit, *s015*) with the ribosomal binding site (RBS) on *geneA_mRNA* or *geneB_mRNA* to form the translational start complex *Rib_s_geneA_mRNA* (*s016*) and *Rib_s_geneB_mRNA* (*s026*).

*geneA_mRNA* + *Rib_s* -> *Rib_s_geneA_mRNA* (*r6*)

*geneB_mRNA* + *Rib_s* -> *Rib_s_geneB_mRNA* (*r14*)

The propensities for these reactions are:

*a_6_ = c_6_*s009*s015/V_R_*

and

*a_14_ = c_14_*s025*s015/V_R_*

Reaction *r7* and *r15*: Dissociation of the ribosomal small subunit from the RBS on the *Rib_s_geneA-mRNA* (*s016*) and *Rib_s_geneB_mRNA* (*s026*).

*Rib_s_geneA_mRNA -> geneA_mRNA + Rib_s* (*r7*)

and

*Rib_s_geneB_mRNA -> geneB_mRNA + Rib_s* (*r15*)

The propensities for these uni- substrate reactions are, respectively:

*a_7_ = c_7_*s016*

and

*a_15_ = c_15_*s026*

Reaction *r8* and *r16*: Elongation of the peptide and ultimate formation of protein A and protein B gene product by the incorporation of the appropriate number of amino acids into the protein polymer. Upon completion of the reaction, the *Pro_A* and *Pro_B* proteins are released and the *geneA_mRNA*, *geneB_mRNA*, ribosomal large subunit (*Rib_l*, *s017*) and the ribosomal small subunit (*Rib_s*) are returned to the available pools for reuse. Guanidine diphosphate (*GDP*, *s019*), adenine diphosphate (*ADP*, *s021*)and inorganic phosphate (*Pi*, *s020*) are formed as by-products.

*Rib_s_geneA_mRNA + Rib_l + 44 A + 9 C + 27 D + 43 E + 22 F + 40 G + 7 H + 31 I +*

*23 K + 53 L + 13 M + 18 N + 25 P + 20 Q + 34 R + 29 S + 29 T + 21 V + 8 W +*

*21 Y + 1552 GTP -> geneA_mRNA + Rib_s + Rib_l + Pro_A +*

*1552 GDP + 2068 Pi* (*r8*)

and

*Rib_s_geneB_mRNA + Rib_l + 44 A + 9 C + 27 D + 43 E + 22 F + 40 G + 7 H + 31 I +*

*23 K + 53 L + 13 M + 18 N + 25 P + 20 Q + 34 R + 29 S + 29 T + 21 V + 8 W +*

*21 Y + 1552 GTP -> geneB_mRNA + Rib_s + Rib_l + Pro_A +*

*1552 GDP + 2068 Pi* (*r16*)

Note, the amino acid composition of *Pro_A* is based on the nucleotide sequence of *geneA*. Once *Rib_s* is bound to the *geneA_mRNA* or *geneB_mRNA* forming the *Rib_s_geneA_mRNA* or *Rib_s_geneB_mRNA* complex, the protein products, *Pro_A* and *Pro_B*, can be assembled via the translation reactions (*r8* and *r16*) with a phenomenological propensity of

*a_8_ = c_8_*Rib_l*f_8_(Rib_s_geneA_mRNA, GTP, AA_A_tRNA_AA_A,*

*AA_C_tRNA_AA_C, ..., AA_V_tRNA_AA_V)/V_R_*

*= c_8_*s017*f_8_(s016, s006, s092, s093, ..., s111)/V_R_*

and

*a_16_ = c_16_*Rib_l*f_16_(Rib_s_geneA_mRNA, GTP, AA_A_tRNA_AA_A,*

*AA_C_tRNA_AA_C, ..., AA_V_tRNA_AA_V)/V_R_*

*= c_16_*s017*f_16_(s016, s006, s092, s093, ..., s111)/V_R_*

where the substrate dependency is given by

$$\begin{matrix} f_{8}(Rib\_s\_geneA\_mRNA,GTP,AA\_A\_Trans\_AA-A,\ldots,AA\_Y\_Trans\_AA\_Y)= \\ \left(\frac{Rib\_s-geneA\_mRNA}{Rib\_s\_geneA\_mRNA+km8\_Rib\_s\_geneA\_mRNA}\right)*\left(\frac{GTP}{GTP+km8\_GTP}\right)* \\ \left(\frac{AA\_A\_Trans\_AA\_A}{AA\_A\_Trans\_AA\_A+km8\_AA\_A\_Trans\_AA\_A}\right)* \\ ... \left(\frac{AA\_Y\_Trans\_AA\_Y}{AA\_Y\_Trans\_AA)\_Y+km8\_AA\_Y\_Trans\_AA\_Y}\right) \end{matrix}$$

or

$$\begin{matrix} f_{8}(s016,s006,s092,\ldots,s111)=\left(\frac{s016}{s016+km8\_s016}\right)*\left(\frac{s006}{s006+km8\_s006}\right)* \\ \left(\frac{s092}{s092+km8\_s092}\right)* ... *\left(\frac{s111}{s111+km8\_s111}\right) \end{matrix}$$

Similar equations hold for *f_16_*. Here, the propensity of the translation reaction is modulated by the availability of the substrates, including the *Rib_s_geneA_mRNA* complex, energy substrates *GTP*, and all of the charged tRNAs (*AA_A_Trans_A_AA* to *AA_Y_Trans_AA_Y*). The modulation factors *f_8_* and *f_16_, 0 ≤ f_5_*, *f_16_ ≤ 1*, are of the form of the product of hyperbolic factors and accounts for certain realistic features of the reaction, namely, a zero propensity when any of the substrates is not available to complete the polymerization reaction and a maximum propensity, *c_8_*Rib_l/V_R_*, when all substrates and energy molecules are available in saturating concentrations.

**Metabolic Reaction**

*Charging of tRNAs*

The translation reactions (*r8* and *r16*) use charged tRNAs as substrates to provide amino acids for protein polymerization. In this model, it is assumed that *geneA* and *geneB* are engineered genes that use single codons for each amino acid in the protein, i.e., there are only 20 codons used to designate the amino acid sequence in the protein products. In this case, there are only 20 tRNAs required to match the 20 codons. Thus, the model contains 20 reaction pathways to charge the tRNAs (see Figure S3). Each of these reaction pathways uses a specific amino-acyl transferase and ATP to transfer a free amino acid to the correct tRNA.

Each tRNA charging reaction pathway consists of 10 association, dissociation and catalytic reactions, beginning with ATP binding to the specific transferase (*AA_i_Trans*, *s052* - *s071*). The sequence of reactions includes binding of the appropriate amino acid, binding of the appropriate tRNA and finally ligation of the two components. In this generic model, all 20 reaction pathways are identical (initial conditions and probabilistic reaction rate constants) except for the number of molecules of each free amino acids. The sequence of reactions are: (1) association and dissociation of ATP, (2) association and dissociation of the specific amino acid (*s032* - *s051*), (3) activation of the bound amino acid and subsequent release of ADP, (4) binding of the specific tRNA (*s072* - *s091*), (5) charging of the tRNA with the amino acid and release of inorganic phosphate (*Pi* - *s020*), and finally, (6) release of the charged tRNA (*s092* - *s111*) and recycling of the transferase. The pool of charged tRNAs serve as the substrates for the translation reaction. All of these reactions are treated as fundamental uni- or bi-substrate reactions with appropriate propensities. These 20 reaction pathways account for 200 reactions in the model (*r17* to *r216*).

*Ligation Reactions*

The enzyme mediated metabolic reactions catalyzed by *Pro_A* and *Pro_B* result in the ligation of *Sub_1* and *Sub_2* to form *Prod_A* and the subsequent ligation of *Prod_A* with *Sub_ 3* to form *Prod_B*, using *ATP* as a source of free energy. Each of these ligation reactions is a series of fundamental association, dissociation and catalytic reactions.

Reaction *r266* - *r275*: Catalytic ligation of *Sub_A* and *Sub_B* to form *Prod_A* using *ATP* as a cofactor. The net reactions is:

*Pro_A* + *Sub_1* + *Sub_2* + *ATP* -> *Pro_A* + *Prod_A* + *ADP* + *Pi*.

The synthetic reaction forming *Prod_A* is mediated by the expressed product of *geneA*. The enzyme *Pro_A* utilizes *ATP* to carry out the ligation of *Sub_1* and *Sub_2*. The sequence of 10 micro-reactions that constitutes the ligation reaction - association and dissociation of substrates *ATP*, *Sub_1* and *Sub_2* and the enzyme, splitting *ATP* to *ADP* and *Pi* to provide free energy for the ligation reaction, and dissociation of *Prod_A* and by-products from the enzyme. The reaction propensities of the micro-reactions are standard Markovian propensities for uni- and bi-substrate reactions. The product of the reaction is *Prod_A* along with the by-products *ADP* and inorganic phosphate.

Reaction *r276* - *r285*: Catalytic ligation of *Prod_A* and *Sub_3* to form *Prod_B* using *ATP* as a cofactor. The net reactions is:

*Pro_B_4* + *Prod_A* + *Sub_3* + *ATP* -> *Pro_B_4* + *Prod_B* + *ADP* + *Pi*.

The synthetic reaction forming *Prod_B* is mediated by the tetramer of the expressed product of *geneB*. The enzyme *Pro_B_4* utilizes *ATP* to carry out the ligation of *Prod_B* and *Sub_3*. The sequence of 10 micro-reactions that constitutes the ligation reaction - association and dissociation of substrates *ATP*, *Prod_A* and *Sub_3* with the enzyme, splitting *ATP* to *ADP* and *Pi* to provide free energy for the ligation reaction, and dissociation of *Prod_B* and by-products from the enzyme. The reaction propensities of the micro-reactions are standard Markovian propensities for uni- and bi-substrate reactions. The product of the reaction is *Prod_B* along with the by-products *ADP* and inorganic phosphate.

**Degradation Reactions**

There are two types of degradation reactions in this model: degradation of mRNA and degradation of the proteins present in the biomolecular reaction network.

*Degradation of RNA*

There are several types of RNA present in this biomolecular reaction network - mRNA, rRNA and tRNA. Degradation of all RNAs are treated in a similar manner.

Reaction *r5*, *r13*, *r217* - *r236*: Reaction *r5* - degradation of *geneA_mRNA* by the generic RNase resulting in the formation of the constituent nucleotide mono-phosphates - is used to illustrate the RNA degradation reactions.

*geneA_mRNA* + *RNase* -> *RNase* + 381 *UMP* + 429 *CMP* + 377 *AMP* + 369 *GMP* (*r5*)

The mRNA degradation reaction (*r5*) accounts for the possibility that *geneA_mRNA* will be degraded by the generic RNase. The products of the degradation process are the nucleotide monophophates (NMPs: *AMP* - *s011*, *GMP* - *s012*, *CMP* - *s013*, *UMP* - *s014*). Here, the mRNA degradation reaction is treated as a lumped macro-reaction using an approximate phenomenological formulation for the propensity.

$$a_{5}=c_{5}*\alpha_{s030}*s030*f_{5}(s009)$$

where

$$f_{5}(s009)=\frac{s009}{s009+k5\_s009}$$

is the hyperbolic functional form of the mRNA substrate and *α_s030_* is the fraction of the RNAase molecules that are free on average (the rest being occupied in other degradation reactions). The stochastic reaction parameter *c_5_* for this reaction is set very low in the current model so that there is little degradation of RNAs.

Similar reactions are used for reaction *r13* (degradation of *geneB_mRNA*) and reactions *r217* - *r236* (degradation of tRNAs). It is assumed that ribosomal RNAs are embedded in the ribosomal subunits are not available for degradation.

*Degradation of Proteins*

There are several proteins present in the system - RNA polymerase, amino acyl-transferases, *Pro_A*, *Pro_B*, the generic RNase, the generic protease - that are subject to degradation by the generic protease (*Prot* - *s031*). Since this model assumes that the same protease degrades all four proteins, then it is necessary to use a slightly modified formulation for the propensity that takes into consideration competition between the substrates for degradation.

Reaction *r237* - *r261*: Reaction *r260* - degradation of *Pro_A* by the generic protease (*Prot*) resulting in the formation of the constituent amino acids is used as an example of protein degradation. The net reaction is:

*Pro_A* + *Prot* -> *Prot* + 44 *A* + 9 *C* + 27 *D* + 43 *E* + 22 *F* + 40 *G* + 7 *H* + 31 *I* + 23 *K* + 53 *L*

+ 13 *M* + 18 *N* + 25 *P* + 20 *Q* + 34 *R* + 29 *S* + 29 *T* + 21 *V* + 8 *W* + 21 *Y* (*r260*)

Since this reaction is treated as a lumped, macro-reaction and the fact that the protease is assumed to degrade all proteins present in the biomolecular reaction network, the phenomenological propensity is given by

$$a_{260}=c_{260}*\alpha_{s031}*s031*f_{260}(s018)$$

where

$$f_{260}=\frac{s018}{s018+km9\_s018}$$

is the hyperbolic form for the substrate and *α_s031_* is the average free fraction of protease molecules. This expression accounts for the saturation of the protease by substrate and the dependence of the propensity on utilization of the enzyme by the other substrates.

Similar expressions are used for the other protein degradation reactions.

**Initial Conditions and Model Parameters**

The initial conditions and model parameters are given in the SBML model description. The initial conditions for state variables are listed in the <listOfCompartments> section and the reaction parameters are given in the individual reactions. Although this is a hypothetical biomolecular reaction network, some of the initial conditions are based on experimental results. The concentrations of all nucleotides and amino acids were experimentally measured in a commercial cell-free transcription-translation system and converted into the number of molecules that would exist in a vesicle of volume 5.0x10^-16^ L. For state variables where concentrations were not measured, we used a nominal concentration of either 1.0 μM (301 molecules) or 2 μM (602 molecules). The substrates for the metabolic reactions were set at 150 μM (45,002 molecules). The amino acid composition hypothetical proteins are given in Table S1.

**Table S1**: Composition of *geneA*, *geneB*, *Pro_A*, *Pro_B*, *RNAp*, *RNase*, *Prot*, *AA_i_Trans* and *AA_i_tRNA* molecules in the geneA_geneB_CFTT_1p1 model.

| **Stoichiometry of Molecules** | | |
| --- | --- | --- |
| Nucleotide composition of the *geneA* and *geneB* | ATP | 381 |
|  | GTP | 429 |
|  | CTP | 369 |
|  | UTP | 377 |
| Energy requirements for *Pro_A* and *Pro_B* formation | ATP | 517 |
|  | GTP | 1552 |
| Amino acid composition of *Pro_A* and *Pro_B* protein | isoleucine | 31 |
|  | methionine | 13 |
|  | threonine | 29 |
|  | aspartic acid | 27 |
|  | proline | 25 |
|  | phenylalanine | 22 |
|  | arginine | 34 |
|  | glycine | 40 |
|  | leucine | 53 |
|  | tryptophan | 8 |
|  | asparagine | 18 |
|  | lysine | 23 |
|  | cysteine | 9 |
|  | histidine | 7 |
|  | glutamine | 20 |
|  | alanine | 44 |
|  | glutamic acid | 43 |
|  | serine | 29 |
|  | tyrosine | 21 |
|  | valine | 21 |
| Amino acid composition of *RNAp* | isoleucine | 20 |
|  | methionine | 20 |
|  | threonine | 20 |
|  | aspartic acid | 20 |
|  | proline | 20 |
|  | phenylalanine | 20 |
|  | arginine | 20 |
|  | glycine | 20 |
|  | leucine | 20 |
|  | tryptophan | 20 |
|  | asparagine | 20 |
|  | lysine | 20 |
|  | cysteine | 20 |
|  | histidine | 20 |
|  | glutamine | 20 |
|  | alanine | 20 |
|  | glutamic acid | 20 |
|  | serine | 20 |
|  | tyrosine | 20 |
|  | valine | 20 |
| Amino acid composition of the generic *RNase* | isoleucine | 20 |
|  | methionine | 20 |
|  | threonine | 20 |
|  | aspartic acid | 20 |
|  | proline | 20 |
|  | phenylalanine | 20 |
|  | arginine | 20 |
|  | glycine | 20 |
|  | leucine | 20 |
|  | tryptophan | 20 |
|  | asparagine | 20 |
|  | lysine | 20 |
|  | cysteine | 20 |
|  | histidine | 20 |
|  | glutamine | 20 |
|  | alanine | 20 |
|  | glutamic acid | 20 |
|  | serine | 20 |
|  | tyrosine | 20 |
|  | valine | 20 |
| Amino acid composition of the generic *Prot* | isoleucine | 20 |
|  | methionine | 20 |
|  | threonine | 20 |
|  | aspartic acid | 20 |
|  | proline | 20 |
|  | phenylalanine | 20 |
|  | arginine | 20 |
|  | glycine | 20 |
|  | leucine | 20 |
|  | tryptophan | 20 |
|  | asparagine | 20 |
|  | lysine | 20 |
|  | cysteine | 20 |
|  | histidine | 20 |
|  | glutamine | 20 |
|  | alanine | 20 |
|  | glutamic acid | 20 |
|  | serine | 20 |
|  | tyrosine | 20 |
|  | valine | 20 |

| Amino acid composition of the 20 *AA_i_Trans* | isoleucine | 20 |
| --- | --- | --- |
|  | methionine | 20 |
|  | threonine | 20 |
|  | aspartic acid | 20 |
|  | proline | 20 |
|  | phenylalanine | 20 |
|  | arginine | 20 |
|  | glycine | 20 |
|  | leucine | 20 |
|  | tryptophan | 20 |
|  | asparagine | 20 |
|  | lysine | 20 |
|  | cysteine | 20 |
|  | histidine | 20 |
|  | glutamine | 20 |
|  | alanine | 20 |
|  | glutamic acid | 20 |
|  | serine | 20 |
|  | tyrosine | 20 |
|  | valine | 20 |

| Nucleotide composition of the 20 *AA_i_tRNAs* | ATP | 25 |
| --- | --- | --- |
|  | GTP | 25 |
|  | CTP | 25 |
|  | UTP | 25 |

Figure S1: The schematic diagram of the conceptual model of the two gene self-assembling biomolecular reaction network (geneA_geneB_CFTT_1p1).


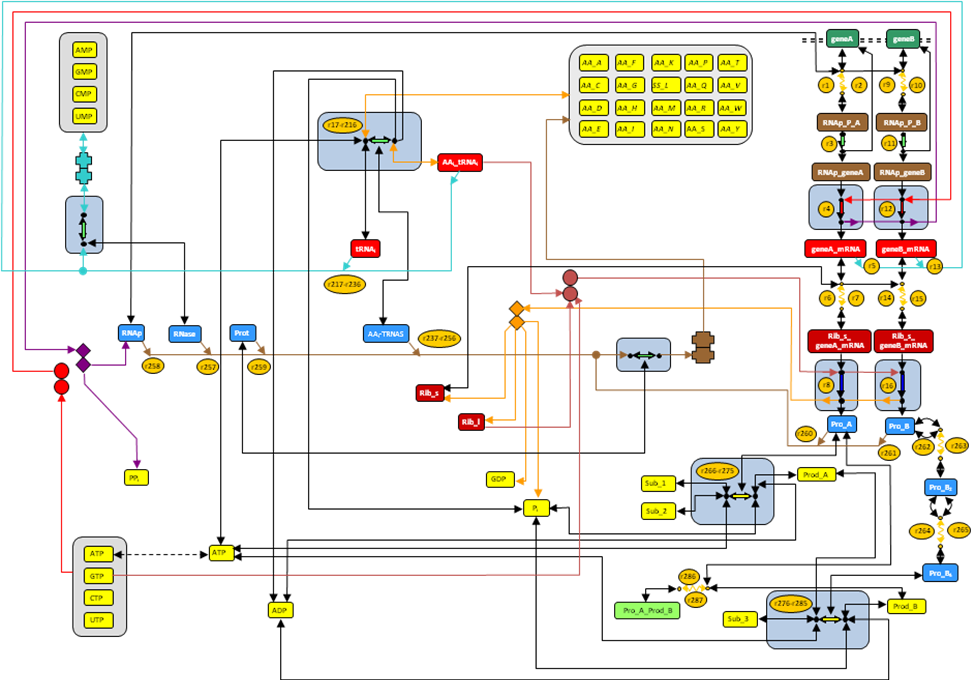


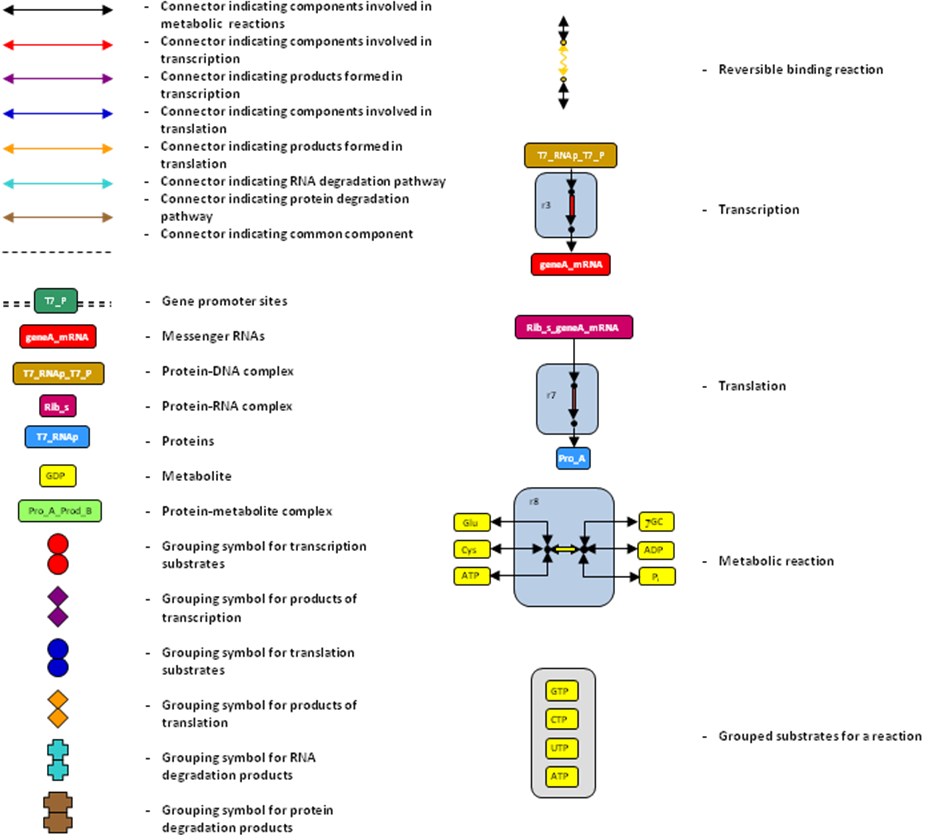


Figure S2(A): Schematic diagram of the mathematical model of the geneA_geneB_CFTT_1p1 model using semi-descriptive nomenclature.


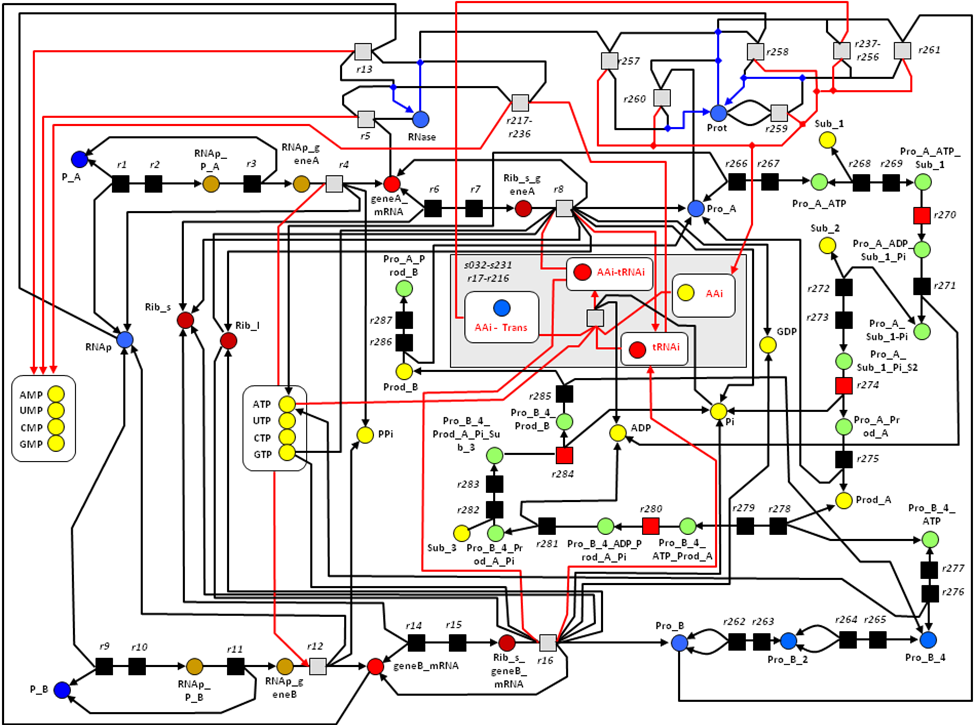


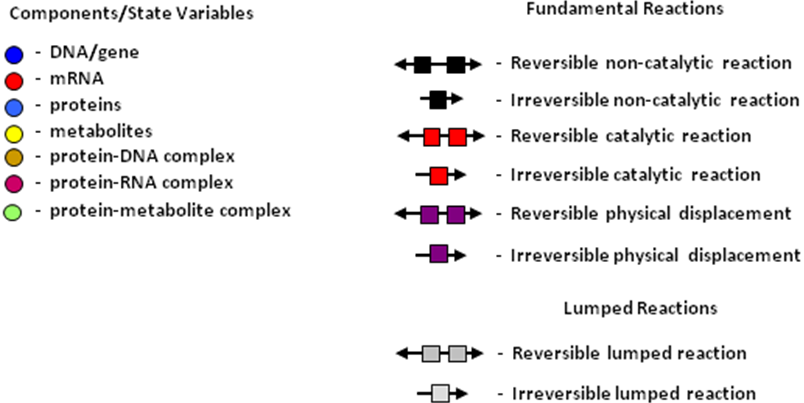


Figure S2(B): Schematic diagram of the mathematical model of the tRNA charging reactions in the geneA_geneB_CFTT_1p1 model using semi-descriptive nomenclature.


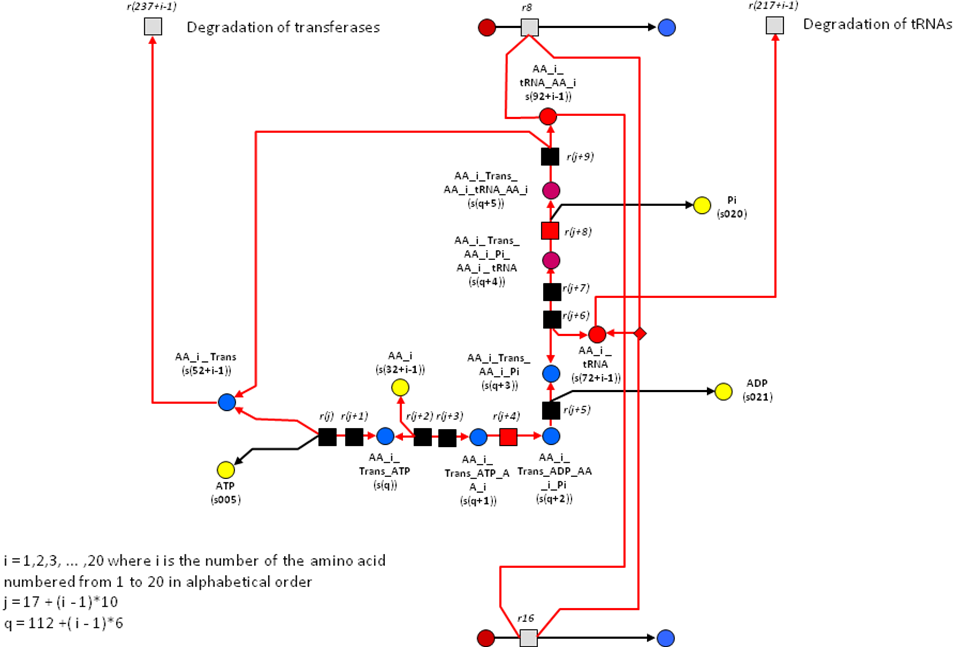


Figure S3: Schematic diagram of the mathematical model of the geneA_geneB_CFTT_1p1 model using internal nomenclature.


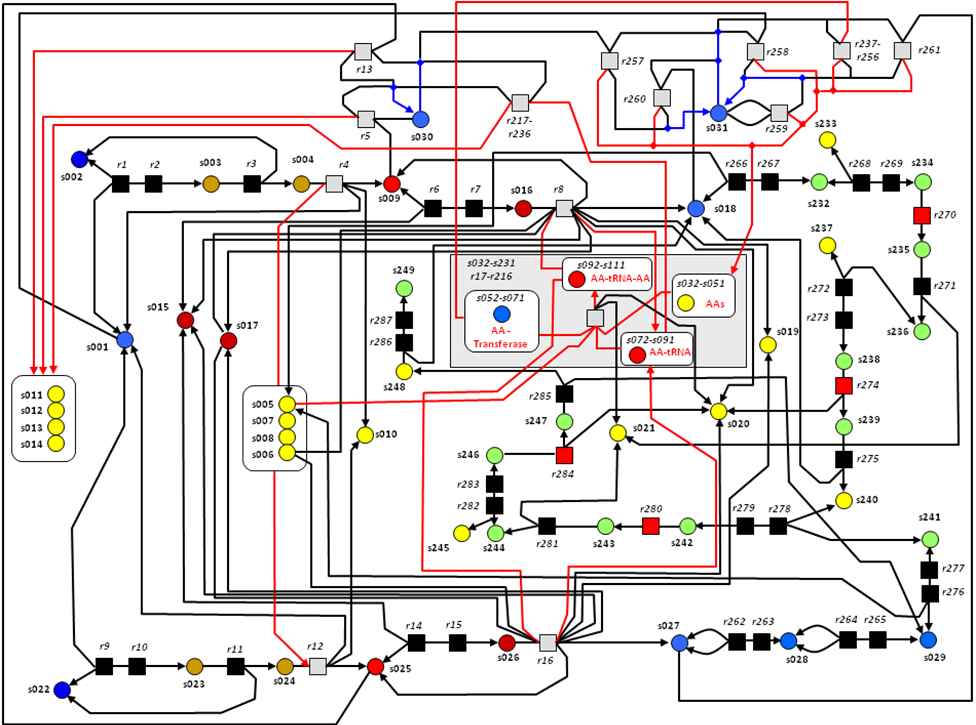

Supplement: Additional File 1 — Model Description. The information provided describes the conceptual structure of the exemplar model and the mathematical description of the model reactions. [file 1752-0509-3-64-S1.doc]
