# Supplementary material for: Stochastic simulation and analysis of biomolecular reaction networks
Source: BMC Syst Biol. 2009 Jun 17;3:64. doi: 10.1186/1752-0509-3-64 (PMC2708125; doi:10.1186/1752-0509-3-64)
Supplement: Additional File 2 — SBML Model Definition. This file contains the SBML model definition code for the exemplar model. [file 1752-0509-3-64-S2.doc]

stochastic simulation and Analysis of

BiomoleculAr Reaction Networks

Additional File 2 - SBML Model Definition

John Frazier

Air Force Research Laboratory

WPAFB, OH 45433-5707

Yaroslav Chushak

Biotechnology HPC Software Applications Institute

US Army Medical Research and Materiel Command

ARLF/RHPB WPAFB, OH 45433-5707

Brent Foy

Department of Physics

Wright State University

Dayton, OH 45435

**Code for the SBML model description of the geneA_geneB_CFTT_1p1 model**

<?xml version="1.0" encoding="UTF-8"?>

<sbml xmlns="http://www.sbml.org/sbml/level1" level="1" version="1">

<model name="geneA">

<listOfCompartments>

<compartment name="" volume="1"/>

</listOfCompartments>

<listOfSpecies>

<specie name="s001" compartment="C1" initialAmount="301"/>

<specie name="s002" compartment="C1" initialAmount="1"/>

<specie name="s003" compartment="C1" initialAmount="0"/>

<specie name="s004" compartment="C1" initialAmount="0"/>

<specie name="s005" compartment="C1" initialAmount="208329"/>

<specie name="s006" compartment="C1" initialAmount="234178"/>

<specie name="s007" compartment="C1" initialAmount="150500"/>

<specie name="s008" compartment="C1" initialAmount="166754"/>

<specie name="s009" compartment="C1" initialAmount="0"/>

<specie name="s010" compartment="C1" initialAmount="0"/>

<specie name="s011" compartment="C1" initialAmount="0"/>

<specie name="s012" compartment="C1" initialAmount="0"/>

<specie name="s013" compartment="C1" initialAmount="6622"/>

<specie name="s014" compartment="C1" initialAmount="0"/>

<specie name="s015" compartment="C1" initialAmount="301"/>

<specie name="s016" compartment="C1" initialAmount="0"/>

<specie name="s017" compartment="C1" initialAmount="301"/>

<specie name="s018" compartment="C1" initialAmount="0"/>

<specie name="s019" compartment="C1" initialAmount="0"/>

<specie name="s020" compartment="C1" initialAmount="0"/>

<specie name="s021" compartment="C1" initialAmount="29197"/>

<specie name="s022" compartment="C1" initialAmount="1"/>

<specie name="s023" compartment="C1" initialAmount="0"/>

<specie name="s024" compartment="C1" initialAmount="0"/>

<specie name="s025" compartment="C1" initialAmount="0"/>

<specie name="s026" compartment="C1" initialAmount="0"/>

<specie name="s027" compartment="C1" initialAmount="0"/>

<specie name="s028" compartment="C1" initialAmount="0"/>

<specie name="s029" compartment="C1" initialAmount="0"/>

<specie name="s030" compartment="C1" initialAmount="301"/>

<specie name="s031" compartment="C1" initialAmount="301"/>

<specie name="s032" compartment="C1" initialAmount="43645"/>

<specie name="s033" compartment="C1" initialAmount="94213"/>

<specie name="s034" compartment="C1" initialAmount="61705"/>

<specie name="s035" compartment="C1" initialAmount="79163"/>

<specie name="s036" compartment="C1" initialAmount="45752"/>

<specie name="s037" compartment="C1" initialAmount="76454"/>

<specie name="s038" compartment="C1" initialAmount="33411"/>

<specie name="s039" compartment="C1" initialAmount="52976"/>

<specie name="s040" compartment="C1" initialAmount="21973"/>

<specie name="s041" compartment="C1" initialAmount="44247"/>

<specie name="s042" compartment="C1" initialAmount="37324"/>

<specie name="s043" compartment="C1" initialAmount="12943"/>

<specie name="s044" compartment="C1" initialAmount="205884"/>

<specie name="s045" compartment="C1" initialAmount="2408"/>

<specie name="s046" compartment="C1" initialAmount="49665"/>

<specie name="s047" compartment="C1" initialAmount="35217"/>

<specie name="s048" compartment="C1" initialAmount="8428"/>

<specie name="s049" compartment="C1" initialAmount="37926"/>

<specie name="s050" compartment="C1" initialAmount="29498"/>

<specie name="s051" compartment="C1" initialAmount="56588"/>

<specie name="s052" compartment="C1" initialAmount="602"/>

<specie name="s053" compartment="C1" initialAmount="602"/>

<specie name="s054" compartment="C1" initialAmount="602"/>

<specie name="s055" compartment="C1" initialAmount="602"/>

<specie name="s056" compartment="C1" initialAmount="602"/>

<specie name="s057" compartment="C1" initialAmount="602"/>

<specie name="s058" compartment="C1" initialAmount="602"/>

<specie name="s059" compartment="C1" initialAmount="602"/>

<specie name="s060" compartment="C1" initialAmount="602"/>

<specie name="s061" compartment="C1" initialAmount="602"/>

<specie name="s062" compartment="C1" initialAmount="602"/>

<specie name="s063" compartment="C1" initialAmount="602"/>

<specie name="s064" compartment="C1" initialAmount="602"/>

<specie name="s065" compartment="C1" initialAmount="602"/>

<specie name="s066" compartment="C1" initialAmount="602"/>

<specie name="s067" compartment="C1" initialAmount="602"/>

<specie name="s068" compartment="C1" initialAmount="602"/>

<specie name="s069" compartment="C1" initialAmount="602"/>

<specie name="s070" compartment="C1" initialAmount="602"/>

<specie name="s071" compartment="C1" initialAmount="602"/>

<specie name="s072" compartment="C1" initialAmount="602"/>

<specie name="s073" compartment="C1" initialAmount="602"/>

<specie name="s074" compartment="C1" initialAmount="602"/>

<specie name="s075" compartment="C1" initialAmount="602"/>

<specie name="s076" compartment="C1" initialAmount="602"/>

<specie name="s077" compartment="C1" initialAmount="602"/>

<specie name="s078" compartment="C1" initialAmount="602"/>

<specie name="s079" compartment="C1" initialAmount="602"/>

<specie name="s080" compartment="C1" initialAmount="602"/>

<specie name="s081" compartment="C1" initialAmount="602"/>

<specie name="s082" compartment="C1" initialAmount="602"/>

<specie name="s083" compartment="C1" initialAmount="602"/>

<specie name="s084" compartment="C1" initialAmount="602"/>

<specie name="s085" compartment="C1" initialAmount="602"/>

<specie name="s086" compartment="C1" initialAmount="602"/>

<specie name="s087" compartment="C1" initialAmount="602"/>

<specie name="s088" compartment="C1" initialAmount="602"/>

<specie name="s089" compartment="C1" initialAmount="602"/>

<specie name="s090" compartment="C1" initialAmount="602"/>

<specie name="s091" compartment="C1" initialAmount="602"/>

<specie name="s092" compartment="C1" initialAmount="0"/>

<specie name="s093" compartment="C1" initialAmount="0"/>

<specie name="s094" compartment="C1" initialAmount="0"/>

<specie name="s095" compartment="C1" initialAmount="0"/>

<specie name="s096" compartment="C1" initialAmount="0"/>

<specie name="s097" compartment="C1" initialAmount="0"/>

<specie name="s098" compartment="C1" initialAmount="0"/>

<specie name="s099" compartment="C1" initialAmount="0"/>

<specie name="s100" compartment="C1" initialAmount="0"/>

<specie name="s101" compartment="C1" initialAmount="0"/>

<specie name="s102" compartment="C1" initialAmount="0"/>

<specie name="s103" compartment="C1" initialAmount="0"/>

<specie name="s104" compartment="C1" initialAmount="0"/>

<specie name="s105" compartment="C1" initialAmount="0"/>

<specie name="s106" compartment="C1" initialAmount="0"/>

<specie name="s107" compartment="C1" initialAmount="0"/>

<specie name="s108" compartment="C1" initialAmount="0"/>

<specie name="s109" compartment="C1" initialAmount="0"/>

<specie name="s110" compartment="C1" initialAmount="0"/>

<specie name="s111" compartment="C1" initialAmount="0"/>

<specie name="s112" compartment="C1" initialAmount="0"/>

<specie name="s113" compartment="C1" initialAmount="0"/>

<specie name="s114" compartment="C1" initialAmount="0"/>

<specie name="s115" compartment="C1" initialAmount="0"/>

<specie name="s116" compartment="C1" initialAmount="0"/>

<specie name="s117" compartment="C1" initialAmount="0"/>

<specie name="s118" compartment="C1" initialAmount="0"/>

<specie name="s119" compartment="C1" initialAmount="0"/>

<specie name="s120" compartment="C1" initialAmount="0"/>

<specie name="s121" compartment="C1" initialAmount="0"/>

<specie name="s122" compartment="C1" initialAmount="0"/>

<specie name="s123" compartment="C1" initialAmount="0"/>

<specie name="s124" compartment="C1" initialAmount="0"/>

<specie name="s125" compartment="C1" initialAmount="0"/>

<specie name="s126" compartment="C1" initialAmount="0"/>

<specie name="s127" compartment="C1" initialAmount="0"/>

<specie name="s128" compartment="C1" initialAmount="0"/>

<specie name="s129" compartment="C1" initialAmount="0"/>

<specie name="s130" compartment="C1" initialAmount="0"/>

<specie name="s131" compartment="C1" initialAmount="0"/>

<specie name="s132" compartment="C1" initialAmount="0"/>

<specie name="s133" compartment="C1" initialAmount="0"/>

<specie name="s134" compartment="C1" initialAmount="0"/>

<specie name="s135" compartment="C1" initialAmount="0"/>

<specie name="s136" compartment="C1" initialAmount="0"/>

<specie name="s137" compartment="C1" initialAmount="0"/>

<specie name="s138" compartment="C1" initialAmount="0"/>

<specie name="s139" compartment="C1" initialAmount="0"/>

<specie name="s140" compartment="C1" initialAmount="0"/>

<specie name="s141" compartment="C1" initialAmount="0"/>

<specie name="s142" compartment="C1" initialAmount="0"/>

<specie name="s143" compartment="C1" initialAmount="0"/>

<specie name="s144" compartment="C1" initialAmount="0"/>

<specie name="s145" compartment="C1" initialAmount="0"/>

<specie name="s146" compartment="C1" initialAmount="0"/>

<specie name="s147" compartment="C1" initialAmount="0"/>

<specie name="s148" compartment="C1" initialAmount="0"/>

<specie name="s149" compartment="C1" initialAmount="0"/>

<specie name="s150" compartment="C1" initialAmount="0"/>

<specie name="s151" compartment="C1" initialAmount="0"/>

<specie name="s152" compartment="C1" initialAmount="0"/>

<specie name="s153" compartment="C1" initialAmount="0"/>

<specie name="s154" compartment="C1" initialAmount="0"/>

<specie name="s155" compartment="C1" initialAmount="0"/>

<specie name="s156" compartment="C1" initialAmount="0"/>

<specie name="s157" compartment="C1" initialAmount="0"/>

<specie name="s158" compartment="C1" initialAmount="0"/>

<specie name="s159" compartment="C1" initialAmount="0"/>

<specie name="s160" compartment="C1" initialAmount="0"/>

<specie name="s161" compartment="C1" initialAmount="0"/>

<specie name="s162" compartment="C1" initialAmount="0"/>

<specie name="s163" compartment="C1" initialAmount="0"/>

<specie name="s164" compartment="C1" initialAmount="0"/>

<specie name="s165" compartment="C1" initialAmount="0"/>

<specie name="s166" compartment="C1" initialAmount="0"/>

<specie name="s167" compartment="C1" initialAmount="0"/>

<specie name="s168" compartment="C1" initialAmount="0"/>

<specie name="s169" compartment="C1" initialAmount="0"/>

<specie name="s170" compartment="C1" initialAmount="0"/>

<specie name="s171" compartment="C1" initialAmount="0"/>

<specie name="s172" compartment="C1" initialAmount="0"/>

<specie name="s173" compartment="C1" initialAmount="0"/>

<specie name="s174" compartment="C1" initialAmount="0"/>

<specie name="s175" compartment="C1" initialAmount="0"/>

<specie name="s176" compartment="C1" initialAmount="0"/>

<specie name="s177" compartment="C1" initialAmount="0"/>

<specie name="s178" compartment="C1" initialAmount="0"/>

<specie name="s179" compartment="C1" initialAmount="0"/>

<specie name="s180" compartment="C1" initialAmount="0"/>

<specie name="s181" compartment="C1" initialAmount="0"/>

<specie name="s182" compartment="C1" initialAmount="0"/>

<specie name="s183" compartment="C1" initialAmount="0"/>

<specie name="s184" compartment="C1" initialAmount="0"/>

<specie name="s185" compartment="C1" initialAmount="0"/>

<specie name="s186" compartment="C1" initialAmount="0"/>

<specie name="s187" compartment="C1" initialAmount="0"/>

<specie name="s188" compartment="C1" initialAmount="0"/>

<specie name="s189" compartment="C1" initialAmount="0"/>

<specie name="s190" compartment="C1" initialAmount="0"/>

<specie name="s191" compartment="C1" initialAmount="0"/>

<specie name="s192" compartment="C1" initialAmount="0"/>

<specie name="s193" compartment="C1" initialAmount="0"/>

<specie name="s194" compartment="C1" initialAmount="0"/>

<specie name="s195" compartment="C1" initialAmount="0"/>

<specie name="s196" compartment="C1" initialAmount="0"/>

<specie name="s197" compartment="C1" initialAmount="0"/>

<specie name="s198" compartment="C1" initialAmount="0"/>

<specie name="s199" compartment="C1" initialAmount="0"/>

<specie name="s200" compartment="C1" initialAmount="0"/>

<specie name="s201" compartment="C1" initialAmount="0"/>

<specie name="s202" compartment="C1" initialAmount="0"/>

<specie name="s203" compartment="C1" initialAmount="0"/>

<specie name="s204" compartment="C1" initialAmount="0"/>

<specie name="s205" compartment="C1" initialAmount="0"/>

<specie name="s206" compartment="C1" initialAmount="0"/>

<specie name="s207" compartment="C1" initialAmount="0"/>

<specie name="s208" compartment="C1" initialAmount="0"/>

<specie name="s209" compartment="C1" initialAmount="0"/>

<specie name="s210" compartment="C1" initialAmount="0"/>

<specie name="s211" compartment="C1" initialAmount="0"/>

<specie name="s212" compartment="C1" initialAmount="0"/>

<specie name="s213" compartment="C1" initialAmount="0"/>

<specie name="s214" compartment="C1" initialAmount="0"/>

<specie name="s215" compartment="C1" initialAmount="0"/>

<specie name="s216" compartment="C1" initialAmount="0"/>

<specie name="s217" compartment="C1" initialAmount="0"/>

<specie name="s218" compartment="C1" initialAmount="0"/>

<specie name="s219" compartment="C1" initialAmount="0"/>

<specie name="s220" compartment="C1" initialAmount="0"/>

<specie name="s221" compartment="C1" initialAmount="0"/>

<specie name="s222" compartment="C1" initialAmount="0"/>

<specie name="s223" compartment="C1" initialAmount="0"/>

<specie name="s224" compartment="C1" initialAmount="0"/>

<specie name="s225" compartment="C1" initialAmount="0"/>

<specie name="s226" compartment="C1" initialAmount="0"/>

<specie name="s227" compartment="C1" initialAmount="0"/>

<specie name="s228" compartment="C1" initialAmount="0"/>

<specie name="s229" compartment="C1" initialAmount="0"/>

<specie name="s230" compartment="C1" initialAmount="0"/>

<specie name="s231" compartment="C1" initialAmount="0"/>

<specie name="s232" compartment="C1" initialAmount="0"/>

<specie name="s233" compartment="C1" initialAmount="40000"/>

<specie name="s234" compartment="C1" initialAmount="0"/>

<specie name="s235" compartment="C1" initialAmount="0"/>

<specie name="s236" compartment="C1" initialAmount="0"/>

<specie name="s237" compartment="C1" initialAmount="45002"/>

<specie name="s238" compartment="C1" initialAmount="0"/>

<specie name="s239" compartment="C1" initialAmount="0"/>

<specie name="s240" compartment="C1" initialAmount="0"/>

<specie name="s241" compartment="C1" initialAmount="0"/>

<specie name="s242" compartment="C1" initialAmount="0"/>

<specie name="s243" compartment="C1" initialAmount="0"/>

<specie name="s244" compartment="C1" initialAmount="0"/>

<specie name="s245" compartment="C1" initialAmount="45002"/>

<specie name="s246" compartment="C1" initialAmount="0"/>

<specie name="s247" compartment="C1" initialAmount="0"/>

<specie name="s248" compartment="C1" initialAmount="0"/>

<specie name="s249" compartment="C1" initialAmount="0"/>

<specie name="t250" compartment="C1" initialAmount="0"/>

<specie name="t251" compartment="C1" initialAmount="0"/>

</listOfSpecies>

<listOfParameters>

<parameter name="vol" value="5e-016"/>

</listOfParameters>

<listOfReactions>

<reaction name="r1" reversible="false">

<listOfReactants>

<specieReference specie="s001"/>

<specieReference specie="s002"/>

</listOfReactants>

<listOfProducts>

<specieReference specie="s003"/>

</listOfProducts>

<kineticLaw formula="c1*s001*s002/vol">

<listOfParameters>

<parameter name="c1" value="1e-018"/>

<parameter name="vol" value="5e-016"/>

</listOfParameters>

</kineticLaw>

</reaction>

<reaction name="r2" reversible="false">

<listOfReactants>

<specieReference specie="s003"/>

</listOfReactants>

<listOfProducts>

<specieReference specie="s001"/>

<specieReference specie="s002"/>

</listOfProducts>

<kineticLaw formula="c2*s003">

<listOfParameters>

<parameter name="c2" value="0.1"/>

</listOfParameters>

</kineticLaw>

</reaction>

<reaction name="r3" reversible="false">

<listOfReactants>

<specieReference specie="s003"/>

</listOfReactants>

<listOfProducts>

<specieReference specie="s002"/>

<specieReference specie="s004"/>

</listOfProducts>

<kineticLaw formula="c3*(1-s004)*s003">

<listOfParameters>

<parameter name="c3" value="0.1"/>

</listOfParameters>

</kineticLaw>

</reaction>

<reaction name="r4" reversible="false">

<listOfReactants>

<specieReference specie="s004"/>

<specieReference specie="s005" stoichiometry="381"/>

<specieReference specie="s006" stoichiometry="429"/>

<specieReference specie="s008" stoichiometry="369"/>

<specieReference specie="s007" stoichiometry="377"/>

</listOfReactants>

<listOfProducts>

<specieReference specie="s001"/>

<specieReference specie="s009"/>

<specieReference specie="s010" stoichiometry="1556"/>

</listOfProducts>

<kineticLaw formula="c4*(s004/vol)*(s005/(s005+km4_s005))*(s006/(s006+km4_s006))*(s007/(s007+km4_s007))*(s008/(s008+km4_s008))">

<listOfParameters>

<parameter name="c4" value="5e-017"/>

<parameter name="vol" value="5e-016"/>

<parameter name="km4_s005" value="301"/>

<parameter name="km4_s006" value="301"/>

<parameter name="km4_s007" value="301"/>

<parameter name="km4_s008" value="301"/>

</listOfParameters>

</kineticLaw>

</reaction>

<reaction name="r5" reversible="false">

<listOfReactants>

<specieReference specie="s009"/>

</listOfReactants>

<listOfProducts>

<specieReference specie="s011" stoichiometry="381"/>

<specieReference specie="s012" stoichiometry="429"/>

<specieReference specie="s013" stoichiometry="369"/>

<specieReference specie="s014" stoichiometry="377"/>

</listOfProducts>

<kineticLaw formula="c5*a_s030*(s030/vol)*s009/(s009+km5_s009)">

<listOfParameters>

<parameter name="c5" value="5e-025"/>

<parameter name="a_s030" value="1"/>

<parameter name="km5_s009" value="301"/>

<parameter name="vol" value="5e-016"/>

</listOfParameters>

</kineticLaw>

</reaction>

<reaction name="r6" reversible="false">

<listOfReactants>

<specieReference specie="s009"/>

<specieReference specie="s015"/>

</listOfReactants>

<listOfProducts>

<specieReference specie="s016"/>

</listOfProducts>

<kineticLaw formula="c6*s015*s009/vol">

<listOfParameters>

<parameter name="c6" value="1e-018"/>

<parameter name="vol" value="5e-016"/>

</listOfParameters>

</kineticLaw>

</reaction>

<reaction name="r7" reversible="false">

<listOfReactants>

<specieReference specie="s016"/>

</listOfReactants>

<listOfProducts>

<specieReference specie="s009"/>

<specieReference specie="s015"/>

</listOfProducts>

<kineticLaw formula="c7*s016">

<listOfParameters>

<parameter name="c7" value="0.1"/>

</listOfParameters>

</kineticLaw>

</reaction>

<reaction name="r8" reversible="false">

<listOfReactants>

<specieReference specie="s016"/>

<specieReference specie="s017"/>

<specieReference specie="s006" stoichiometry="1552"/>

<specieReference specie="s092" stoichiometry="44"/>

<specieReference specie="s093" stoichiometry="9"/>

<specieReference specie="s094" stoichiometry="27"/>

<specieReference specie="s095" stoichiometry="43"/>

<specieReference specie="s096" stoichiometry="22"/>

<specieReference specie="s097" stoichiometry="40"/>

<specieReference specie="s098" stoichiometry="7"/>

<specieReference specie="s099" stoichiometry="31"/>

<specieReference specie="s100" stoichiometry="23"/>

<specieReference specie="s101" stoichiometry="53"/>

<specieReference specie="s102" stoichiometry="13"/>

<specieReference specie="s103" stoichiometry="18"/>

<specieReference specie="s104" stoichiometry="25"/>

<specieReference specie="s105" stoichiometry="20"/>

<specieReference specie="s106" stoichiometry="34"/>

<specieReference specie="s107" stoichiometry="29"/>

<specieReference specie="s108" stoichiometry="29"/>

<specieReference specie="s109" stoichiometry="8"/>

<specieReference specie="s110" stoichiometry="21"/>

<specieReference specie="s111" stoichiometry="21"/>

</listOfReactants>

<listOfProducts>

<specieReference specie="s009"/>

<specieReference specie="s015"/>

<specieReference specie="s017"/>

<specieReference specie="s018"/>

<specieReference specie="s020" stoichiometry="1552"/>

<specieReference specie="s019" stoichiometry="1552"/>

<specieReference specie="s072" stoichiometry="44"/>

<specieReference specie="s073" stoichiometry="9"/>

<specieReference specie="s074" stoichiometry="27"/>

<specieReference specie="s075" stoichiometry="43"/>

<specieReference specie="s076" stoichiometry="22"/>

<specieReference specie="s077" stoichiometry="40"/>

<specieReference specie="s078" stoichiometry="7"/>

<specieReference specie="s079" stoichiometry="31"/>

<specieReference specie="s080" stoichiometry="23"/>

<specieReference specie="s081" stoichiometry="53"/>

<specieReference specie="s082" stoichiometry="13"/>

<specieReference specie="s083" stoichiometry="18"/>

<specieReference specie="s084" stoichiometry="25"/>

<specieReference specie="s085" stoichiometry="20"/>

<specieReference specie="s086" stoichiometry="34"/>

<specieReference specie="s087" stoichiometry="29"/>

<specieReference specie="s088" stoichiometry="29"/>

<specieReference specie="s089" stoichiometry="8"/>

<specieReference specie="s090" stoichiometry="21"/>

<specieReference specie="s091" stoichiometry="21"/>

<specieReference specie="t250"/>

</listOfProducts>

<kineticLaw formula="c8*(s017/vol)*(s016/(s016+km8_s016))*(s006/(s006+km8_s006))*(s092/(s092+km8_s092))*(s093/(s093+km8_s093))*(s094/(s094+km8_s094))*(s095/(s095+km8_s095))*(s096/(s096+km8_s096))*(s097/(s097+km8_s097))*(s098/(s098+km8_s098))*(s099/(s099+km8_s099))*(s100/(s100+km8_s100))*(s101/(s101+km8_s101))*(s102/(s102+km8_s102))*(s103/(s103+km8_s103))*(s104/(s104+km8_s104))*(s105/(s105+km8_s105))*(s106/(s106+km8_s106))*(s107/(s107+km8_s107))*(s108/(s108+km8_s108))*(s109/(s109+km8_s109))*(s110/(s110+km8_s110))*(s111/(s111+km8_s111))">

<listOfParameters>

<parameter name="c8" value="7.85e-020"/>

<parameter name="vol" value="5e-016"/>

<parameter name="km8_s016" value="3"/>

<parameter name="km8_s006" value="301"/>

<parameter name="km8_s092" value="3"/>

<parameter name="km8_s093" value="3"/>

<parameter name="km8_s094" value="3"/>

<parameter name="km8_s095" value="3"/>

<parameter name="km8_s096" value="3"/>

<parameter name="km8_s097" value="3"/>

<parameter name="km8_s098" value="3"/>

<parameter name="km8_s099" value="3"/>

<parameter name="km8_s100" value="3"/>

<parameter name="km8_s101" value="3"/>

<parameter name="km8_s102" value="3"/>

<parameter name="km8_s103" value="3"/>

<parameter name="km8_s104" value="3"/>

<parameter name="km8_s105" value="3"/>

<parameter name="km8_s106" value="3"/>

<parameter name="km8_s107" value="3"/>

<parameter name="km8_s108" value="3"/>

<parameter name="km8_s109" value="3"/>

<parameter name="km8_s110" value="3"/>

<parameter name="km8_s111" value="3"/>

</listOfParameters>

</kineticLaw>

</reaction>

<reaction name="r9" reversible="false">

<listOfReactants>

<specieReference specie="s001"/>

<specieReference specie="s022"/>

</listOfReactants>

<listOfProducts>

<specieReference specie="s023"/>

</listOfProducts>

<kineticLaw formula="c9*s001*s022/vol">

<listOfParameters>

<parameter name="c9" value="1e-018"/>

<parameter name="vol" value="5e-016"/>

</listOfParameters>

</kineticLaw>

</reaction>

<reaction name="r10" reversible="false">

<listOfReactants>

<specieReference specie="s023"/>

</listOfReactants>

<listOfProducts>

<specieReference specie="s001"/>

<specieReference specie="s022"/>

</listOfProducts>

<kineticLaw formula="c10*s023">

<listOfParameters>

<parameter name="c10" value="0.1"/>

</listOfParameters>

</kineticLaw>

</reaction>

<reaction name="r11" reversible="false">

<listOfReactants>

<specieReference specie="s023"/>

</listOfReactants>

<listOfProducts>

<specieReference specie="s022"/>

<specieReference specie="s024"/>

</listOfProducts>

<kineticLaw formula="c11*(1-s024)*s023">

<listOfParameters>

<parameter name="c11" value="0.1"/>

</listOfParameters>

</kineticLaw>

</reaction>

<reaction name="r12" reversible="false">

<listOfReactants>

<specieReference specie="s024"/>

<specieReference specie="s005" stoichiometry="381"/>

<specieReference specie="s006" stoichiometry="429"/>

<specieReference specie="s008" stoichiometry="369"/>

<specieReference specie="s007" stoichiometry="377"/>

</listOfReactants>

<listOfProducts>

<specieReference specie="s001"/>

<specieReference specie="s025"/>

<specieReference specie="s010" stoichiometry="1556"/>

</listOfProducts>

<kineticLaw formula="c12*(s024/vol)*(s005/(s005+km12_s005))*(s006/(s006+km12_s006))*(s007/(s007+km12_s007))*(s008/(s008+km12_s008))">

<listOfParameters>

<parameter name="c12" value="5e-017"/>

<parameter name="vol" value="5e-016"/>

<parameter name="km12_s005" value="301"/>

<parameter name="km12_s006" value="301"/>

<parameter name="km12_s007" value="301"/>

<parameter name="km12_s008" value="301"/>

</listOfParameters>

</kineticLaw>

</reaction>

<reaction name="r13" reversible="false">

<listOfReactants>

<specieReference specie="s025"/>

</listOfReactants>

<listOfProducts>

<specieReference specie="s011" stoichiometry="381"/>

<specieReference specie="s012" stoichiometry="429"/>

<specieReference specie="s013" stoichiometry="369"/>

<specieReference specie="s014" stoichiometry="377"/>

</listOfProducts>

<kineticLaw formula="c13*a_s030*(s030/vol)*s025/(s025+km13_s025)">

<listOfParameters>

<parameter name="c13" value="5e-025"/>

<parameter name="a_s030" value="1"/>

<parameter name="km13_s025" value="301"/>

<parameter name="vol" value="5e-016"/>

</listOfParameters>

</kineticLaw>

</reaction>

<reaction name="r14" reversible="false">

<listOfReactants>

<specieReference specie="s025"/>

<specieReference specie="s015"/>

</listOfReactants>

<listOfProducts>

<specieReference specie="s026"/>

</listOfProducts>

<kineticLaw formula="c14*s015*s025/vol">

<listOfParameters>

<parameter name="c14" value="1e-018"/>

<parameter name="vol" value="5e-016"/>

</listOfParameters>

</kineticLaw>

</reaction>

<reaction name="r15" reversible="false">

<listOfReactants>

<specieReference specie="s026"/>

</listOfReactants>

<listOfProducts>

<specieReference specie="s025"/>

<specieReference specie="s015"/>

</listOfProducts>

<kineticLaw formula="c15*s026">

<listOfParameters>

<parameter name="c15" value="0.1"/>

</listOfParameters>

</kineticLaw>

</reaction>

<reaction name="r16" reversible="false">

<listOfReactants>

<specieReference specie="s026"/>

<specieReference specie="s006" stoichiometry="1552"/>

<specieReference specie="s017"/>

<specieReference specie="s092" stoichiometry="44"/>

<specieReference specie="s093" stoichiometry="9"/>

<specieReference specie="s094" stoichiometry="27"/>

<specieReference specie="s095" stoichiometry="43"/>

<specieReference specie="s096" stoichiometry="22"/>

<specieReference specie="s097" stoichiometry="40"/>

<specieReference specie="s098" stoichiometry="7"/>

<specieReference specie="s099" stoichiometry="31"/>

<specieReference specie="s100" stoichiometry="23"/>

<specieReference specie="s101" stoichiometry="53"/>

<specieReference specie="s102" stoichiometry="13"/>

<specieReference specie="s103" stoichiometry="18"/>

<specieReference specie="s104" stoichiometry="25"/>

<specieReference specie="s105" stoichiometry="20"/>

<specieReference specie="s106" stoichiometry="34"/>

<specieReference specie="s107" stoichiometry="29"/>

<specieReference specie="s108" stoichiometry="29"/>

<specieReference specie="s109" stoichiometry="8"/>

<specieReference specie="s110" stoichiometry="21"/>

<specieReference specie="s111" stoichiometry="21"/>

</listOfReactants>

<listOfProducts>

<specieReference specie="s025"/>

<specieReference specie="s015"/>

<specieReference specie="s017"/>

<specieReference specie="s027"/>

<specieReference specie="s020" stoichiometry="1552"/>

<specieReference specie="s019" stoichiometry="1552"/>

<specieReference specie="s072" stoichiometry="44"/>

<specieReference specie="s073" stoichiometry="9"/>

<specieReference specie="s074" stoichiometry="27"/>

<specieReference specie="s075" stoichiometry="43"/>

<specieReference specie="s076" stoichiometry="22"/>

<specieReference specie="s077" stoichiometry="40"/>

<specieReference specie="s078" stoichiometry="7"/>

<specieReference specie="s079" stoichiometry="31"/>

<specieReference specie="s080" stoichiometry="23"/>

<specieReference specie="s081" stoichiometry="53"/>

<specieReference specie="s082" stoichiometry="13"/>

<specieReference specie="s083" stoichiometry="18"/>

<specieReference specie="s084" stoichiometry="25"/>

<specieReference specie="s085" stoichiometry="20"/>

<specieReference specie="s086" stoichiometry="34"/>

<specieReference specie="s087" stoichiometry="29"/>

<specieReference specie="s088" stoichiometry="29"/>

<specieReference specie="s089" stoichiometry="8"/>

<specieReference specie="s090" stoichiometry="21"/>

<specieReference specie="s091" stoichiometry="21"/>

<specieReference specie="t251"/>

</listOfProducts>

<kineticLaw formula="c16*(s017/vol)*(s026/(s026+km16_s026))*(s006/(s006+km16_s006))*(s092/(s092+km16_s092))*(s093/(s093+km16_s093))*(s094/(s094+km16_s094))*(s095/(s095+km16_s095))*(s096/(s096+km16_s096))*(s097/(s097+km16_s097))*(s098/(s098+km16_s098))*(s099/(s099+km16_s099))*(s100/(s100+km16_s100))*(s101/(s101+km16_s101))*(s102/(s102+km16_s102))*(s103/(s103+km16_s103))*(s104/(s104+km16_s104))*(s105/(s105+km16_s105))*(s106/(s106+km16_s106))*(s107/(s107+km16_s107))*(s108/(s108+km16_s108))*(s109/(s109+km16_s109))*(s110/(s110+km16_s110))*(s111/(s111+km16_s111))">

<listOfParameters>

<parameter name="c16" value="7.85e-020"/>

<parameter name="vol" value="5e-016"/>

<parameter name="km16_s026" value="3"/>

<parameter name="km16_s006" value="301"/>

<parameter name="km16_s092" value="3"/>

<parameter name="km16_s093" value="3"/>

<parameter name="km16_s094" value="3"/>

<parameter name="km16_s095" value="3"/>

<parameter name="km16_s096" value="3"/>

<parameter name="km16_s097" value="3"/>

<parameter name="km16_s098" value="3"/>

<parameter name="km16_s099" value="3"/>

<parameter name="km16_s100" value="3"/>

<parameter name="km16_s101" value="3"/>

<parameter name="km16_s102" value="3"/>

<parameter name="km16_s103" value="3"/>

<parameter name="km16_s104" value="3"/>

<parameter name="km16_s105" value="3"/>

<parameter name="km16_s106" value="3"/>

<parameter name="km16_s107" value="3"/>

<parameter name="km16_s108" value="3"/>

<parameter name="km16_s109" value="3"/>

<parameter name="km16_s110" value="3"/>

<parameter name="km16_s111" value="3"/>

</listOfParameters>

</kineticLaw>

</reaction>

<reaction name="r17" reversible="false">

<listOfReactants>

<specieReference specie="s052"/>

<specieReference specie="s005"/>

</listOfReactants>

<listOfProducts>

<specieReference specie="s112"/>

</listOfProducts>

<kineticLaw formula="c17*s052*s005/vol">

<listOfParameters>

<parameter name="c17" value="1e-018"/>

<parameter name="vol" value="5e-016"/>

</listOfParameters>

</kineticLaw>

</reaction>

<reaction name="r18" reversible="false">

<listOfReactants>

<specieReference specie="s112"/>

</listOfReactants>

<listOfProducts>

<specieReference specie="s052"/>

<specieReference specie="s005"/>

</listOfProducts>

<kineticLaw formula="c18*s112">

<listOfParameters>

<parameter name="c18" value="0.1"/>

</listOfParameters>

</kineticLaw>

</reaction>

<reaction name="r19" reversible="false">

<listOfReactants>

<specieReference specie="s112"/>

<specieReference specie="s032"/>

</listOfReactants>

<listOfProducts>

<specieReference specie="s113"/>

</listOfProducts>

<kineticLaw formula="c19*s112*s032/vol">

<listOfParameters>

<parameter name="c19" value="1e-018"/>

<parameter name="vol" value="5e-016"/>

</listOfParameters>

</kineticLaw>

</reaction>

<reaction name="r20" reversible="false">

<listOfReactants>

<specieReference specie="s113"/>

</listOfReactants>

<listOfProducts>

<specieReference specie="s112"/>

<specieReference specie="s032"/>

</listOfProducts>

<kineticLaw formula="c20*s113">

<listOfParameters>

<parameter name="c20" value="0.1"/>

</listOfParameters>

</kineticLaw>

</reaction>

<reaction name="r21" reversible="false">

<listOfReactants>

<specieReference specie="s113"/>

</listOfReactants>

<listOfProducts>

<specieReference specie="s114"/>

</listOfProducts>

<kineticLaw formula="c21*s113">

<listOfParameters>

<parameter name="c21" value="0.1"/>

</listOfParameters>

</kineticLaw>

</reaction>

<reaction name="r22" reversible="false">

<listOfReactants>

<specieReference specie="s114"/>

</listOfReactants>

<listOfProducts>

<specieReference specie="s115"/>

<specieReference specie="s021"/>

</listOfProducts>

<kineticLaw formula="c22*s114">

<listOfParameters>

<parameter name="c22" value="0.1"/>

</listOfParameters>

</kineticLaw>

</reaction>

<reaction name="r23" reversible="false">

<listOfReactants>

<specieReference specie="s115"/>

<specieReference specie="s072"/>

</listOfReactants>

<listOfProducts>

<specieReference specie="s116"/>

</listOfProducts>

<kineticLaw formula="c23*s115*s072/vol">

<listOfParameters>

<parameter name="c23" value="1e-018"/>

<parameter name="vol" value="5e-016"/>

</listOfParameters>

</kineticLaw>

</reaction>

<reaction name="r24" reversible="false">

<listOfReactants>

<specieReference specie="s116"/>

</listOfReactants>

<listOfProducts>

<specieReference specie="s115"/>

<specieReference specie="s072"/>

</listOfProducts>

<kineticLaw formula="c24*s116">

<listOfParameters>

<parameter name="c24" value="0.1"/>

</listOfParameters>

</kineticLaw>

</reaction>

<reaction name="r25" reversible="false">

<listOfReactants>

<specieReference specie="s116"/>

</listOfReactants>

<listOfProducts>

<specieReference specie="s117"/>

<specieReference specie="s020"/>

</listOfProducts>

<kineticLaw formula="c25*s116">

<listOfParameters>

<parameter name="c25" value="0.1"/>

</listOfParameters>

</kineticLaw>

</reaction>

<reaction name="r26" reversible="false">

<listOfReactants>

<specieReference specie="s117"/>

</listOfReactants>

<listOfProducts>

<specieReference specie="s092"/>

<specieReference specie="s052"/>

</listOfProducts>

<kineticLaw formula="c26*s117">

<listOfParameters>

<parameter name="c26" value="0.1"/>

</listOfParameters>

</kineticLaw>

</reaction>

<reaction name="r27" reversible="false">

<listOfReactants>

<specieReference specie="s053"/>

<specieReference specie="s005"/>

</listOfReactants>

<listOfProducts>

<specieReference specie="s118"/>

</listOfProducts>

<kineticLaw formula="c27*s053*s005/vol">

<listOfParameters>

<parameter name="c27" value="1e-018"/>

<parameter name="vol" value="5e-016"/>

</listOfParameters>

</kineticLaw>

</reaction>

<reaction name="r28" reversible="false">

<listOfReactants>

<specieReference specie="s118"/>

</listOfReactants>

<listOfProducts>

<specieReference specie="s053"/>

<specieReference specie="s005"/>

</listOfProducts>

<kineticLaw formula="c28*s118">

<listOfParameters>

<parameter name="c28" value="0.1"/>

</listOfParameters>

</kineticLaw>

</reaction>

<reaction name="r29" reversible="false">

<listOfReactants>

<specieReference specie="s118"/>

<specieReference specie="s033"/>

</listOfReactants>

<listOfProducts>

<specieReference specie="s119"/>

</listOfProducts>

<kineticLaw formula="c29*s118*s033/vol">

<listOfParameters>

<parameter name="c29" value="1e-018"/>

<parameter name="vol" value="5e-016"/>

</listOfParameters>

</kineticLaw>

</reaction>

<reaction name="r30" reversible="false">

<listOfReactants>

<specieReference specie="s119"/>

</listOfReactants>

<listOfProducts>

<specieReference specie="s118"/>

<specieReference specie="s033"/>

</listOfProducts>

<kineticLaw formula="c30*s119">

<listOfParameters>

<parameter name="c30" value="0.1"/>

</listOfParameters>

</kineticLaw>

</reaction>

<reaction name="r31" reversible="false">

<listOfReactants>

<specieReference specie="s119"/>

</listOfReactants>

<listOfProducts>

<specieReference specie="s120"/>

</listOfProducts>

<kineticLaw formula="c31*s119">

<listOfParameters>

<parameter name="c31" value="0.1"/>

</listOfParameters>

</kineticLaw>

</reaction>

<reaction name="r32" reversible="false">

<listOfReactants>

<specieReference specie="s120"/>

</listOfReactants>

<listOfProducts>

<specieReference specie="s121"/>

<specieReference specie="s021"/>

</listOfProducts>

<kineticLaw formula="c32*s120">

<listOfParameters>

<parameter name="c32" value="0.1"/>

</listOfParameters>

</kineticLaw>

</reaction>

<reaction name="r33" reversible="false">

<listOfReactants>

<specieReference specie="s121"/>

<specieReference specie="s073"/>

</listOfReactants>

<listOfProducts>

<specieReference specie="s122"/>

</listOfProducts>

<kineticLaw formula="c33*s121*s073/vol">

<listOfParameters>

<parameter name="c33" value="1e-018"/>

<parameter name="vol" value="5e-016"/>

</listOfParameters>

</kineticLaw>

</reaction>

<reaction name="r34" reversible="false">

<listOfReactants>

<specieReference specie="s122"/>

</listOfReactants>

<listOfProducts>

<specieReference specie="s121"/>

<specieReference specie="s073"/>

</listOfProducts>

<kineticLaw formula="c34*s122">

<listOfParameters>

<parameter name="c34" value="0.1"/>

</listOfParameters>

</kineticLaw>

</reaction>

<reaction name="r35" reversible="false">

<listOfReactants>

<specieReference specie="s122"/>

</listOfReactants>

<listOfProducts>

<specieReference specie="s123"/>

<specieReference specie="s020"/>

</listOfProducts>

<kineticLaw formula="c35*s122">

<listOfParameters>

<parameter name="c35" value="0.1"/>

</listOfParameters>

</kineticLaw>

</reaction>

<reaction name="r36" reversible="false">

<listOfReactants>

<specieReference specie="s123"/>

</listOfReactants>

<listOfProducts>

<specieReference specie="s093"/>

<specieReference specie="s053"/>

</listOfProducts>

<kineticLaw formula="c36*s123">

<listOfParameters>

<parameter name="c36" value="0.1"/>

</listOfParameters>

</kineticLaw>

</reaction>

<reaction name="r37" reversible="false">

<listOfReactants>

<specieReference specie="s054"/>

<specieReference specie="s005"/>

</listOfReactants>

<listOfProducts>

<specieReference specie="s124"/>

</listOfProducts>

<kineticLaw formula="c37*s054*s005/vol">

<listOfParameters>

<parameter name="c37" value="1e-018"/>

<parameter name="vol" value="5e-016"/>

</listOfParameters>

</kineticLaw>

</reaction>

<reaction name="r38" reversible="false">

<listOfReactants>

<specieReference specie="s124"/>

</listOfReactants>

<listOfProducts>

<specieReference specie="s054"/>

<specieReference specie="s005"/>

</listOfProducts>

<kineticLaw formula="c38*s124">

<listOfParameters>

<parameter name="c38" value="0.1"/>

</listOfParameters>

</kineticLaw>

</reaction>

<reaction name="r39" reversible="false">

<listOfReactants>

<specieReference specie="s124"/>

<specieReference specie="s034"/>

</listOfReactants>

<listOfProducts>

<specieReference specie="s125"/>

</listOfProducts>

<kineticLaw formula="c39*s124*s034/vol">

<listOfParameters>

<parameter name="c39" value="1e-018"/>

<parameter name="vol" value="5e-016"/>

</listOfParameters>

</kineticLaw>

</reaction>

<reaction name="r40" reversible="false">

<listOfReactants>

<specieReference specie="s125"/>

</listOfReactants>

<listOfProducts>

<specieReference specie="s124"/>

<specieReference specie="s034"/>

</listOfProducts>

<kineticLaw formula="c40*s125">

<listOfParameters>

<parameter name="c40" value="0.1"/>

</listOfParameters>

</kineticLaw>

</reaction>

<reaction name="r41" reversible="false">

<listOfReactants>

<specieReference specie="s125"/>

</listOfReactants>

<listOfProducts>

<specieReference specie="s126"/>

</listOfProducts>

<kineticLaw formula="c41*s125">

<listOfParameters>

<parameter name="c41" value="0.1"/>

</listOfParameters>

</kineticLaw>

</reaction>

<reaction name="r42" reversible="false">

<listOfReactants>

<specieReference specie="s126"/>

</listOfReactants>

<listOfProducts>

<specieReference specie="s127"/>

<specieReference specie="s021"/>

</listOfProducts>

<kineticLaw formula="c42*s126">

<listOfParameters>

<parameter name="c42" value="0.1"/>

</listOfParameters>

</kineticLaw>

</reaction>

<reaction name="r43" reversible="false">

<listOfReactants>

<specieReference specie="s127"/>

<specieReference specie="s074"/>

</listOfReactants>

<listOfProducts>

<specieReference specie="s128"/>

</listOfProducts>

<kineticLaw formula="c43*s127*s074/vol">

<listOfParameters>

<parameter name="c43" value="1e-018"/>

<parameter name="vol" value="5e-016"/>

</listOfParameters>

</kineticLaw>

</reaction>

<reaction name="r44" reversible="false">

<listOfReactants>

<specieReference specie="s128"/>

</listOfReactants>

<listOfProducts>

<specieReference specie="s127"/>

<specieReference specie="s074"/>

</listOfProducts>

<kineticLaw formula="c44*s128">

<listOfParameters>

<parameter name="c44" value="0.1"/>

</listOfParameters>

</kineticLaw>

</reaction>

<reaction name="r45" reversible="false">

<listOfReactants>

<specieReference specie="s128"/>

</listOfReactants>

<listOfProducts>

<specieReference specie="s129"/>

<specieReference specie="s020"/>

</listOfProducts>

<kineticLaw formula="c45*s128">

<listOfParameters>

<parameter name="c45" value="0.1"/>

</listOfParameters>

</kineticLaw>

</reaction>

<reaction name="r46" reversible="false">

<listOfReactants>

<specieReference specie="s129"/>

</listOfReactants>

<listOfProducts>

<specieReference specie="s094"/>

<specieReference specie="s054"/>

</listOfProducts>

<kineticLaw formula="c46*s129">

<listOfParameters>

<parameter name="c46" value="0.1"/>

</listOfParameters>

</kineticLaw>

</reaction>

<reaction name="r47" reversible="false">

<listOfReactants>

<specieReference specie="s055"/>

<specieReference specie="s005"/>

</listOfReactants>

<listOfProducts>

<specieReference specie="s130"/>

</listOfProducts>

<kineticLaw formula="c47*s055*s005/vol">

<listOfParameters>

<parameter name="c47" value="1e-018"/>

<parameter name="vol" value="5e-016"/>

</listOfParameters>

</kineticLaw>

</reaction>

<reaction name="r48" reversible="false">

<listOfReactants>

<specieReference specie="s130"/>

</listOfReactants>

<listOfProducts>

<specieReference specie="s055"/>

<specieReference specie="s005"/>

</listOfProducts>

<kineticLaw formula="c48*s130">

<listOfParameters>

<parameter name="c48" value="0.1"/>

</listOfParameters>

</kineticLaw>

</reaction>

<reaction name="r49" reversible="false">

<listOfReactants>

<specieReference specie="s130"/>

<specieReference specie="s035"/>

</listOfReactants>

<listOfProducts>

<specieReference specie="s131"/>

</listOfProducts>

<kineticLaw formula="c49*s130*s035/vol">

<listOfParameters>

<parameter name="c49" value="1e-018"/>

<parameter name="vol" value="5e-016"/>

</listOfParameters>

</kineticLaw>

</reaction>

<reaction name="r50" reversible="false">

<listOfReactants>

<specieReference specie="s131"/>

</listOfReactants>

<listOfProducts>

<specieReference specie="s130"/>

<specieReference specie="s035"/>

</listOfProducts>

<kineticLaw formula="c50*s131">

<listOfParameters>

<parameter name="c50" value="0.1"/>

</listOfParameters>

</kineticLaw>

</reaction>

<reaction name="r51" reversible="false">

<listOfReactants>

<specieReference specie="s131"/>

</listOfReactants>

<listOfProducts>

<specieReference specie="s132"/>

</listOfProducts>

<kineticLaw formula="c51*s131">

<listOfParameters>

<parameter name="c51" value="0.1"/>

</listOfParameters>

</kineticLaw>

</reaction>

<reaction name="r52" reversible="false">

<listOfReactants>

<specieReference specie="s132"/>

</listOfReactants>

<listOfProducts>

<specieReference specie="s133"/>

<specieReference specie="s021"/>

</listOfProducts>

<kineticLaw formula="c52*s132">

<listOfParameters>

<parameter name="c52" value="0.1"/>

</listOfParameters>

</kineticLaw>

</reaction>

<reaction name="r53" reversible="false">

<listOfReactants>

<specieReference specie="s133"/>

<specieReference specie="s075"/>

</listOfReactants>

<listOfProducts>

<specieReference specie="s134"/>

</listOfProducts>

<kineticLaw formula="c53*s133*s075/vol">

<listOfParameters>

<parameter name="c53" value="1e-018"/>

<parameter name="vol" value="5e-016"/>

</listOfParameters>

</kineticLaw>

</reaction>

<reaction name="r54" reversible="false">

<listOfReactants>

<specieReference specie="s134"/>

</listOfReactants>

<listOfProducts>

<specieReference specie="s133"/>

<specieReference specie="s075"/>

</listOfProducts>

<kineticLaw formula="c54*s134">

<listOfParameters>

<parameter name="c54" value="0.1"/>

</listOfParameters>

</kineticLaw>

</reaction>

<reaction name="r55" reversible="false">

<listOfReactants>

<specieReference specie="s134"/>

</listOfReactants>

<listOfProducts>

<specieReference specie="s135"/>

<specieReference specie="s020"/>

</listOfProducts>

<kineticLaw formula="c55*s134">

<listOfParameters>

<parameter name="c55" value="0.1"/>

</listOfParameters>

</kineticLaw>

</reaction>

<reaction name="r56" reversible="false">

<listOfReactants>

<specieReference specie="s135"/>

</listOfReactants>

<listOfProducts>

<specieReference specie="s095"/>

<specieReference specie="s055"/>

</listOfProducts>

<kineticLaw formula="c56*s135">

<listOfParameters>

<parameter name="c56" value="0.1"/>

</listOfParameters>

</kineticLaw>

</reaction>

<reaction name="r57" reversible="false">

<listOfReactants>

<specieReference specie="s056"/>

<specieReference specie="s005"/>

</listOfReactants>

<listOfProducts>

<specieReference specie="s136"/>

</listOfProducts>

<kineticLaw formula="c57*s056*s005/vol">

<listOfParameters>

<parameter name="c57" value="1e-018"/>

<parameter name="vol" value="5e-016"/>

</listOfParameters>

</kineticLaw>

</reaction>

<reaction name="r58" reversible="false">

<listOfReactants>

<specieReference specie="s136"/>

</listOfReactants>

<listOfProducts>

<specieReference specie="s056"/>

<specieReference specie="s005"/>

</listOfProducts>

<kineticLaw formula="c58*s136">

<listOfParameters>

<parameter name="c58" value="0.1"/>

</listOfParameters>

</kineticLaw>

</reaction>

<reaction name="r59" reversible="false">

<listOfReactants>

<specieReference specie="s136"/>

<specieReference specie="s036"/>

</listOfReactants>

<listOfProducts>

<specieReference specie="s137"/>

</listOfProducts>

<kineticLaw formula="c59*s136*s036/vol">

<listOfParameters>

<parameter name="c59" value="1e-018"/>

<parameter name="vol" value="5e-016"/>

</listOfParameters>

</kineticLaw>

</reaction>

<reaction name="r60" reversible="false">

<listOfReactants>

<specieReference specie="s137"/>

</listOfReactants>

<listOfProducts>

<specieReference specie="s136"/>

<specieReference specie="s036"/>

</listOfProducts>

<kineticLaw formula="c60*s137">

<listOfParameters>

<parameter name="c60" value="0.1"/>

</listOfParameters>

</kineticLaw>

</reaction>

<reaction name="r61" reversible="false">

<listOfReactants>

<specieReference specie="s137"/>

</listOfReactants>

<listOfProducts>

<specieReference specie="s138"/>

</listOfProducts>

<kineticLaw formula="c61*s137">

<listOfParameters>

<parameter name="c61" value="0.1"/>

</listOfParameters>

</kineticLaw>

</reaction>

<reaction name="r62" reversible="false">

<listOfReactants>

<specieReference specie="s138"/>

</listOfReactants>

<listOfProducts>

<specieReference specie="s139"/>

<specieReference specie="s021"/>

</listOfProducts>

<kineticLaw formula="c62*s138">

<listOfParameters>

<parameter name="c62" value="0.1"/>

</listOfParameters>

</kineticLaw>

</reaction>

<reaction name="r63" reversible="false">

<listOfReactants>

<specieReference specie="s139"/>

<specieReference specie="s076"/>

</listOfReactants>

<listOfProducts>

<specieReference specie="s140"/>

</listOfProducts>

<kineticLaw formula="c63*s139*s076/vol">

<listOfParameters>

<parameter name="c63" value="1e-018"/>

<parameter name="vol" value="5e-016"/>

</listOfParameters>

</kineticLaw>

</reaction>

<reaction name="r64" reversible="false">

<listOfReactants>

<specieReference specie="s140"/>

</listOfReactants>

<listOfProducts>

<specieReference specie="s139"/>

<specieReference specie="s076"/>

</listOfProducts>

<kineticLaw formula="c64*s140">

<listOfParameters>

<parameter name="c64" value="0.1"/>

</listOfParameters>

</kineticLaw>

</reaction>

<reaction name="r65" reversible="false">

<listOfReactants>

<specieReference specie="s140"/>

</listOfReactants>

<listOfProducts>

<specieReference specie="s141"/>

<specieReference specie="s020"/>

</listOfProducts>

<kineticLaw formula="c65*s140">

<listOfParameters>

<parameter name="c65" value="0.1"/>

</listOfParameters>

</kineticLaw>

</reaction>

<reaction name="r66" reversible="false">

<listOfReactants>

<specieReference specie="s141"/>

</listOfReactants>

<listOfProducts>

<specieReference specie="s096"/>

<specieReference specie="s056"/>

</listOfProducts>

<kineticLaw formula="c66*s141">

<listOfParameters>

<parameter name="c66" value="0.1"/>

</listOfParameters>

</kineticLaw>

</reaction>

<reaction name="r67" reversible="false">

<listOfReactants>

<specieReference specie="s057"/>

<specieReference specie="s005"/>

</listOfReactants>

<listOfProducts>

<specieReference specie="s142"/>

</listOfProducts>

<kineticLaw formula="c67*s057*s005/vol">

<listOfParameters>

<parameter name="c67" value="1e-018"/>

<parameter name="vol" value="5e-016"/>

</listOfParameters>

</kineticLaw>

</reaction>

<reaction name="r68" reversible="false">

<listOfReactants>

<specieReference specie="s142"/>

</listOfReactants>

<listOfProducts>

<specieReference specie="s057"/>

<specieReference specie="s005"/>

</listOfProducts>

<kineticLaw formula="c68*s142">

<listOfParameters>

<parameter name="c68" value="0.1"/>

</listOfParameters>

</kineticLaw>

</reaction>

<reaction name="r69" reversible="false">

<listOfReactants>

<specieReference specie="s142"/>

<specieReference specie="s037"/>

</listOfReactants>

<listOfProducts>

<specieReference specie="s143"/>

</listOfProducts>

<kineticLaw formula="c69*s142*s037/vol">

<listOfParameters>

<parameter name="c69" value="1e-018"/>

<parameter name="vol" value="5e-016"/>

</listOfParameters>

</kineticLaw>

</reaction>

<reaction name="r70" reversible="false">

<listOfReactants>

<specieReference specie="s143"/>

</listOfReactants>

<listOfProducts>

<specieReference specie="s142"/>

<specieReference specie="s037"/>

</listOfProducts>

<kineticLaw formula="c70*s143">

<listOfParameters>

<parameter name="c70" value="0.1"/>

</listOfParameters>

</kineticLaw>

</reaction>

<reaction name="r71" reversible="false">

<listOfReactants>

<specieReference specie="s143"/>

</listOfReactants>

<listOfProducts>

<specieReference specie="s144"/>

</listOfProducts>

<kineticLaw formula="c71*s143">

<listOfParameters>

<parameter name="c71" value="0.1"/>

</listOfParameters>

</kineticLaw>

</reaction>

<reaction name="r72" reversible="false">

<listOfReactants>

<specieReference specie="s144"/>

</listOfReactants>

<listOfProducts>

<specieReference specie="s145"/>

<specieReference specie="s021"/>

</listOfProducts>

<kineticLaw formula="c72*s144">

<listOfParameters>

<parameter name="c72" value="0.1"/>

</listOfParameters>

</kineticLaw>

</reaction>

<reaction name="r73" reversible="false">

<listOfReactants>

<specieReference specie="s145"/>

<specieReference specie="s077"/>

</listOfReactants>

<listOfProducts>

<specieReference specie="s146"/>

</listOfProducts>

<kineticLaw formula="c73*s145*s077/vol">

<listOfParameters>

<parameter name="c73" value="1e-018"/>

<parameter name="vol" value="5e-016"/>

</listOfParameters>

</kineticLaw>

</reaction>

<reaction name="r74" reversible="false">

<listOfReactants>

<specieReference specie="s146"/>

</listOfReactants>

<listOfProducts>

<specieReference specie="s145"/>

<specieReference specie="s077"/>

</listOfProducts>

<kineticLaw formula="c74*s146">

<listOfParameters>

<parameter name="c74" value="0.1"/>

</listOfParameters>

</kineticLaw>

</reaction>

<reaction name="r75" reversible="false">

<listOfReactants>

<specieReference specie="s146"/>

</listOfReactants>

<listOfProducts>

<specieReference specie="s147"/>

<specieReference specie="s020"/>

</listOfProducts>

<kineticLaw formula="c75*s146">

<listOfParameters>

<parameter name="c75" value="0.1"/>

</listOfParameters>

</kineticLaw>

</reaction>

<reaction name="r76" reversible="false">

<listOfReactants>

<specieReference specie="s147"/>

</listOfReactants>

<listOfProducts>

<specieReference specie="s097"/>

<specieReference specie="s057"/>

</listOfProducts>

<kineticLaw formula="c76*s147">

<listOfParameters>

<parameter name="c76" value="0.1"/>

</listOfParameters>

</kineticLaw>

</reaction>

<reaction name="r77" reversible="false">

<listOfReactants>

<specieReference specie="s058"/>

<specieReference specie="s005"/>

</listOfReactants>

<listOfProducts>

<specieReference specie="s148"/>

</listOfProducts>

<kineticLaw formula="c77*s058*s005/vol">

<listOfParameters>

<parameter name="c77" value="1e-018"/>

<parameter name="vol" value="5e-016"/>

</listOfParameters>

</kineticLaw>

</reaction>

<reaction name="r78" reversible="false">

<listOfReactants>

<specieReference specie="s148"/>

</listOfReactants>

<listOfProducts>

<specieReference specie="s058"/>

<specieReference specie="s005"/>

</listOfProducts>

<kineticLaw formula="c78*s148">

<listOfParameters>

<parameter name="c78" value="0.1"/>

</listOfParameters>

</kineticLaw>

</reaction>

<reaction name="r79" reversible="false">

<listOfReactants>

<specieReference specie="s148"/>

<specieReference specie="s038"/>

</listOfReactants>

<listOfProducts>

<specieReference specie="s149"/>

</listOfProducts>

<kineticLaw formula="c79*s148*s038/vol">

<listOfParameters>

<parameter name="c79" value="1e-018"/>

<parameter name="vol" value="5e-016"/>

</listOfParameters>

</kineticLaw>

</reaction>

<reaction name="r80" reversible="false">

<listOfReactants>

<specieReference specie="s149"/>

</listOfReactants>

<listOfProducts>

<specieReference specie="s148"/>

<specieReference specie="s038"/>

</listOfProducts>

<kineticLaw formula="c70*s149">

<listOfParameters>

<parameter name="c70" value="0.1"/>

</listOfParameters>

</kineticLaw>

</reaction>

<reaction name="r81" reversible="false">

<listOfReactants>

<specieReference specie="s149"/>

</listOfReactants>

<listOfProducts>

<specieReference specie="s150"/>

</listOfProducts>

<kineticLaw formula="c81*s149">

<listOfParameters>

<parameter name="c81" value="0.1"/>

</listOfParameters>

</kineticLaw>

</reaction>

<reaction name="r82" reversible="false">

<listOfReactants>

<specieReference specie="s150"/>

</listOfReactants>

<listOfProducts>

<specieReference specie="s151"/>

<specieReference specie="s021"/>

</listOfProducts>

<kineticLaw formula="c82*s150">

<listOfParameters>

<parameter name="c82" value="0.1"/>

</listOfParameters>

</kineticLaw>

</reaction>

<reaction name="r83" reversible="false">

<listOfReactants>

<specieReference specie="s151"/>

<specieReference specie="s078"/>

</listOfReactants>

<listOfProducts>

<specieReference specie="s152"/>

</listOfProducts>

<kineticLaw formula="c83*s151*s078/vol">

<listOfParameters>

<parameter name="c83" value="1e-018"/>

<parameter name="vol" value="5e-016"/>

</listOfParameters>

</kineticLaw>

</reaction>

<reaction name="r84" reversible="false">

<listOfReactants>

<specieReference specie="s152"/>

</listOfReactants>

<listOfProducts>

<specieReference specie="s151"/>

<specieReference specie="s078"/>

</listOfProducts>

<kineticLaw formula="c84*s152">

<listOfParameters>

<parameter name="c84" value="0.1"/>

</listOfParameters>

</kineticLaw>

</reaction>

<reaction name="r85" reversible="false">

<listOfReactants>

<specieReference specie="s152"/>

</listOfReactants>

<listOfProducts>

<specieReference specie="s153"/>

<specieReference specie="s020"/>

</listOfProducts>

<kineticLaw formula="c85*s152">

<listOfParameters>

<parameter name="c85" value="0.1"/>

</listOfParameters>

</kineticLaw>

</reaction>

<reaction name="r86" reversible="false">

<listOfReactants>

<specieReference specie="s153"/>

</listOfReactants>

<listOfProducts>

<specieReference specie="s098"/>

<specieReference specie="s058"/>

</listOfProducts>

<kineticLaw formula="c86*s153">

<listOfParameters>

<parameter name="c86" value="0.1"/>

</listOfParameters>

</kineticLaw>

</reaction>

<reaction name="r87" reversible="false">

<listOfReactants>

<specieReference specie="s059"/>

<specieReference specie="s005"/>

</listOfReactants>

<listOfProducts>

<specieReference specie="s154"/>

</listOfProducts>

<kineticLaw formula="c87*s059*s005/vol">

<listOfParameters>

<parameter name="c87" value="1e-018"/>

<parameter name="vol" value="5e-016"/>

</listOfParameters>

</kineticLaw>

</reaction>

<reaction name="r88" reversible="false">

<listOfReactants>

<specieReference specie="s154"/>

</listOfReactants>

<listOfProducts>

<specieReference specie="s059"/>

<specieReference specie="s005"/>

</listOfProducts>

<kineticLaw formula="c88*s154">

<listOfParameters>

<parameter name="c88" value="0.1"/>

</listOfParameters>

</kineticLaw>

</reaction>

<reaction name="r89" reversible="false">

<listOfReactants>

<specieReference specie="s154"/>

<specieReference specie="s039"/>

</listOfReactants>

<listOfProducts>

<specieReference specie="s155"/>

</listOfProducts>

<kineticLaw formula="c89*s154*s039/vol">

<listOfParameters>

<parameter name="c89" value="1e-018"/>

<parameter name="vol" value="5e-016"/>

</listOfParameters>

</kineticLaw>

</reaction>

<reaction name="r90" reversible="false">

<listOfReactants>

<specieReference specie="s155"/>

</listOfReactants>

<listOfProducts>

<specieReference specie="s154"/>

<specieReference specie="s039"/>

</listOfProducts>

<kineticLaw formula="c90*s155">

<listOfParameters>

<parameter name="c90" value="0.1"/>

</listOfParameters>

</kineticLaw>

</reaction>

<reaction name="r91" reversible="false">

<listOfReactants>

<specieReference specie="s155"/>

</listOfReactants>

<listOfProducts>

<specieReference specie="s156"/>

</listOfProducts>

<kineticLaw formula="c91*s155">

<listOfParameters>

<parameter name="c91" value="0.1"/>

</listOfParameters>

</kineticLaw>

</reaction>

<reaction name="r92" reversible="false">

<listOfReactants>

<specieReference specie="s156"/>

</listOfReactants>

<listOfProducts>

<specieReference specie="s157"/>

<specieReference specie="s021"/>

</listOfProducts>

<kineticLaw formula="c92*s156">

<listOfParameters>

<parameter name="c92" value="0.1"/>

</listOfParameters>

</kineticLaw>

</reaction>

<reaction name="r93" reversible="false">

<listOfReactants>

<specieReference specie="s157"/>

<specieReference specie="s079"/>

</listOfReactants>

<listOfProducts>

<specieReference specie="s158"/>

</listOfProducts>

<kineticLaw formula="c93*s157*s079/vol">

<listOfParameters>

<parameter name="c93" value="1e-018"/>

<parameter name="vol" value="5e-016"/>

</listOfParameters>

</kineticLaw>

</reaction>

<reaction name="r94" reversible="false">

<listOfReactants>

<specieReference specie="s158"/>

</listOfReactants>

<listOfProducts>

<specieReference specie="s157"/>

<specieReference specie="s079"/>

</listOfProducts>

<kineticLaw formula="c94*s158">

<listOfParameters>

<parameter name="c94" value="0.1"/>

</listOfParameters>

</kineticLaw>

</reaction>

<reaction name="r95" reversible="false">

<listOfReactants>

<specieReference specie="s158"/>

</listOfReactants>

<listOfProducts>

<specieReference specie="s159"/>

<specieReference specie="s020"/>

</listOfProducts>

<kineticLaw formula="c95*s158">

<listOfParameters>

<parameter name="c95" value="0.1"/>

</listOfParameters>

</kineticLaw>

</reaction>

<reaction name="r96" reversible="false">

<listOfReactants>

<specieReference specie="s159"/>

</listOfReactants>

<listOfProducts>

<specieReference specie="s099"/>

<specieReference specie="s059"/>

</listOfProducts>

<kineticLaw formula="c96*s159">

<listOfParameters>

<parameter name="c96" value="0.1"/>

</listOfParameters>

</kineticLaw>

</reaction>

<reaction name="r97" reversible="false">

<listOfReactants>

<specieReference specie="s060"/>

<specieReference specie="s005"/>

</listOfReactants>

<listOfProducts>

<specieReference specie="s160"/>

</listOfProducts>

<kineticLaw formula="c97*s060*s005/vol">

<listOfParameters>

<parameter name="c97" value="1e-018"/>

<parameter name="vol" value="5e-016"/>

</listOfParameters>

</kineticLaw>

</reaction>

<reaction name="r98" reversible="false">

<listOfReactants>

<specieReference specie="s160"/>

</listOfReactants>

<listOfProducts>

<specieReference specie="s060"/>

<specieReference specie="s005"/>

</listOfProducts>

<kineticLaw formula="c98*s160">

<listOfParameters>

<parameter name="c98" value="0.1"/>

</listOfParameters>

</kineticLaw>

</reaction>

<reaction name="r99" reversible="false">

<listOfReactants>

<specieReference specie="s160"/>

<specieReference specie="s040"/>

</listOfReactants>

<listOfProducts>

<specieReference specie="s161"/>

</listOfProducts>

<kineticLaw formula="c99*s160*s040/vol">

<listOfParameters>

<parameter name="c99" value="1e-018"/>

<parameter name="vol" value="5e-016"/>

</listOfParameters>

</kineticLaw>

</reaction>

<reaction name="r100" reversible="false">

<listOfReactants>

<specieReference specie="s161"/>

</listOfReactants>

<listOfProducts>

<specieReference specie="s160"/>

<specieReference specie="s040"/>

</listOfProducts>

<kineticLaw formula="c100*s161">

<listOfParameters>

<parameter name="c100" value="0.1"/>

</listOfParameters>

</kineticLaw>

</reaction>

<reaction name="r101" reversible="false">

<listOfReactants>

<specieReference specie="s161"/>

</listOfReactants>

<listOfProducts>

<specieReference specie="s162"/>

</listOfProducts>

<kineticLaw formula="c101*s161">

<listOfParameters>

<parameter name="c101" value="0.1"/>

</listOfParameters>

</kineticLaw>

</reaction>

<reaction name="r102" reversible="false">

<listOfReactants>

<specieReference specie="s162"/>

</listOfReactants>

<listOfProducts>

<specieReference specie="s163"/>

<specieReference specie="s021"/>

</listOfProducts>

<kineticLaw formula="c102*s162">

<listOfParameters>

<parameter name="c102" value="0.1"/>

</listOfParameters>

</kineticLaw>

</reaction>

<reaction name="r103" reversible="false">

<listOfReactants>

<specieReference specie="s163"/>

<specieReference specie="s080"/>

</listOfReactants>

<listOfProducts>

<specieReference specie="s164"/>

</listOfProducts>

<kineticLaw formula="c103*s163*s080/vol">

<listOfParameters>

<parameter name="c103" value="1e-018"/>

<parameter name="vol" value="5e-016"/>

</listOfParameters>

</kineticLaw>

</reaction>

<reaction name="r104" reversible="false">

<listOfReactants>

<specieReference specie="s164"/>

</listOfReactants>

<listOfProducts>

<specieReference specie="s163"/>

<specieReference specie="s080"/>

</listOfProducts>

<kineticLaw formula="c104*s164">

<listOfParameters>

<parameter name="c104" value="0.1"/>

</listOfParameters>

</kineticLaw>

</reaction>

<reaction name="r105" reversible="false">

<listOfReactants>

<specieReference specie="s164"/>

</listOfReactants>

<listOfProducts>

<specieReference specie="s165"/>

<specieReference specie="s020"/>

</listOfProducts>

<kineticLaw formula="c105*s164">

<listOfParameters>

<parameter name="c105" value="0.1"/>

</listOfParameters>

</kineticLaw>

</reaction>

<reaction name="r106" reversible="false">

<listOfReactants>

<specieReference specie="s165"/>

</listOfReactants>

<listOfProducts>

<specieReference specie="s100"/>

<specieReference specie="s060"/>

</listOfProducts>

<kineticLaw formula="c106*s165">

<listOfParameters>

<parameter name="c106" value="0.1"/>

</listOfParameters>

</kineticLaw>

</reaction>

<reaction name="r107" reversible="false">

<listOfReactants>

<specieReference specie="s061"/>

<specieReference specie="s005"/>

</listOfReactants>

<listOfProducts>

<specieReference specie="s166"/>

</listOfProducts>

<kineticLaw formula="c107*s061*s005/vol">

<listOfParameters>

<parameter name="c107" value="1e-018"/>

<parameter name="vol" value="5e-016"/>

</listOfParameters>

</kineticLaw>

</reaction>

<reaction name="r108" reversible="false">

<listOfReactants>

<specieReference specie="s166"/>

</listOfReactants>

<listOfProducts>

<specieReference specie="s061"/>

<specieReference specie="s005"/>

</listOfProducts>

<kineticLaw formula="c108*s166">

<listOfParameters>

<parameter name="c108" value="0.1"/>

</listOfParameters>

</kineticLaw>

</reaction>

<reaction name="r109" reversible="false">

<listOfReactants>

<specieReference specie="s166"/>

<specieReference specie="s041"/>

</listOfReactants>

<listOfProducts>

<specieReference specie="s167"/>

</listOfProducts>

<kineticLaw formula="c109*s166*s041/vol">

<listOfParameters>

<parameter name="c109" value="1e-018"/>

<parameter name="vol" value="5e-016"/>

</listOfParameters>

</kineticLaw>

</reaction>

<reaction name="r110" reversible="false">

<listOfReactants>

<specieReference specie="s167"/>

</listOfReactants>

<listOfProducts>

<specieReference specie="s166"/>

<specieReference specie="s041"/>

</listOfProducts>

<kineticLaw formula="c110*s167">

<listOfParameters>

<parameter name="c110" value="0.1"/>

</listOfParameters>

</kineticLaw>

</reaction>

<reaction name="r111" reversible="false">

<listOfReactants>

<specieReference specie="s167"/>

</listOfReactants>

<listOfProducts>

<specieReference specie="s168"/>

</listOfProducts>

<kineticLaw formula="c111*s167">

<listOfParameters>

<parameter name="c111" value="0.1"/>

</listOfParameters>

</kineticLaw>

</reaction>

<reaction name="r112" reversible="false">

<listOfReactants>

<specieReference specie="s168"/>

</listOfReactants>

<listOfProducts>

<specieReference specie="s169"/>

<specieReference specie="s021"/>

</listOfProducts>

<kineticLaw formula="c112*s168">

<listOfParameters>

<parameter name="c112" value="0.1"/>

</listOfParameters>

</kineticLaw>

</reaction>

<reaction name="r113" reversible="false">

<listOfReactants>

<specieReference specie="s169"/>

<specieReference specie="s081"/>

</listOfReactants>

<listOfProducts>

<specieReference specie="s170"/>

</listOfProducts>

<kineticLaw formula="c113*s169*s081/vol">

<listOfParameters>

<parameter name="c113" value="1e-018"/>

<parameter name="vol" value="5e-016"/>

</listOfParameters>

</kineticLaw>

</reaction>

<reaction name="r114" reversible="false">

<listOfReactants>

<specieReference specie="s170"/>

</listOfReactants>

<listOfProducts>

<specieReference specie="s169"/>

<specieReference specie="s081"/>

</listOfProducts>

<kineticLaw formula="c114*s170">

<listOfParameters>

<parameter name="c114" value="0.1"/>

</listOfParameters>

</kineticLaw>

</reaction>

<reaction name="r115" reversible="false">

<listOfReactants>

<specieReference specie="s170"/>

</listOfReactants>

<listOfProducts>

<specieReference specie="s171"/>

<specieReference specie="s020"/>

</listOfProducts>

<kineticLaw formula="c115*s170">

<listOfParameters>

<parameter name="c115" value="0.1"/>

</listOfParameters>

</kineticLaw>

</reaction>

<reaction name="r116" reversible="false">

<listOfReactants>

<specieReference specie="s171"/>

</listOfReactants>

<listOfProducts>

<specieReference specie="s101"/>

<specieReference specie="s061"/>

</listOfProducts>

<kineticLaw formula="c116*s171">

<listOfParameters>

<parameter name="c116" value="0.1"/>

</listOfParameters>

</kineticLaw>

</reaction>

<reaction name="r117" reversible="false">

<listOfReactants>

<specieReference specie="s062"/>

<specieReference specie="s005"/>

</listOfReactants>

<listOfProducts>

<specieReference specie="s172"/>

</listOfProducts>

<kineticLaw formula="c117*s062*s005/vol">

<listOfParameters>

<parameter name="c117" value="1e-018"/>

<parameter name="vol" value="5e-016"/>

</listOfParameters>

</kineticLaw>

</reaction>

<reaction name="r118" reversible="false">

<listOfReactants>

<specieReference specie="s172"/>

</listOfReactants>

<listOfProducts>

<specieReference specie="s062"/>

<specieReference specie="s005"/>

</listOfProducts>

<kineticLaw formula="c118*s172">

<listOfParameters>

<parameter name="c118" value="0.1"/>

</listOfParameters>

</kineticLaw>

</reaction>

<reaction name="r119" reversible="false">

<listOfReactants>

<specieReference specie="s172"/>

<specieReference specie="s042"/>

</listOfReactants>

<listOfProducts>

<specieReference specie="s173"/>

</listOfProducts>

<kineticLaw formula="c119*s172*s042/vol">

<listOfParameters>

<parameter name="c119" value="1e-018"/>

<parameter name="vol" value="5e-016"/>

</listOfParameters>

</kineticLaw>

</reaction>

<reaction name="r120" reversible="false">

<listOfReactants>

<specieReference specie="s173"/>

</listOfReactants>

<listOfProducts>

<specieReference specie="s172"/>

<specieReference specie="s042"/>

</listOfProducts>

<kineticLaw formula="c120*s173">

<listOfParameters>

<parameter name="c120" value="0.1"/>

</listOfParameters>

</kineticLaw>

</reaction>

<reaction name="r121" reversible="false">

<listOfReactants>

<specieReference specie="s173"/>

</listOfReactants>

<listOfProducts>

<specieReference specie="s174"/>

</listOfProducts>

<kineticLaw formula="c121*s173">

<listOfParameters>

<parameter name="c121" value="0.1"/>

</listOfParameters>

</kineticLaw>

</reaction>

<reaction name="r122" reversible="false">

<listOfReactants>

<specieReference specie="s174"/>

</listOfReactants>

<listOfProducts>

<specieReference specie="s175"/>

<specieReference specie="s021"/>

</listOfProducts>

<kineticLaw formula="c122*s174">

<listOfParameters>

<parameter name="c122" value="0.1"/>

</listOfParameters>

</kineticLaw>

</reaction>

<reaction name="r123" reversible="false">

<listOfReactants>

<specieReference specie="s175"/>

<specieReference specie="s082"/>

</listOfReactants>

<listOfProducts>

<specieReference specie="s176"/>

</listOfProducts>

<kineticLaw formula="c123*s175*s082/vol">

<listOfParameters>

<parameter name="c123" value="1e-018"/>

<parameter name="vol" value="5e-016"/>

</listOfParameters>

</kineticLaw>

</reaction>

<reaction name="r124" reversible="false">

<listOfReactants>

<specieReference specie="s176"/>

</listOfReactants>

<listOfProducts>

<specieReference specie="s175"/>

<specieReference specie="s082"/>

</listOfProducts>

<kineticLaw formula="c124*s176">

<listOfParameters>

<parameter name="c124" value="0.1"/>

</listOfParameters>

</kineticLaw>

</reaction>

<reaction name="r125" reversible="false">

<listOfReactants>

<specieReference specie="s176"/>

</listOfReactants>

<listOfProducts>

<specieReference specie="s177"/>

<specieReference specie="s020"/>

</listOfProducts>

<kineticLaw formula="c125*s176">

<listOfParameters>

<parameter name="c125" value="0.1"/>

</listOfParameters>

</kineticLaw>

</reaction>

<reaction name="r126" reversible="false">

<listOfReactants>

<specieReference specie="s177"/>

</listOfReactants>

<listOfProducts>

<specieReference specie="s102"/>

<specieReference specie="s062"/>

</listOfProducts>

<kineticLaw formula="c126*s177">

<listOfParameters>

<parameter name="c126" value="0.1"/>

</listOfParameters>

</kineticLaw>

</reaction>

<reaction name="r127" reversible="false">

<listOfReactants>

<specieReference specie="s063"/>

<specieReference specie="s005"/>

</listOfReactants>

<listOfProducts>

<specieReference specie="s178"/>

</listOfProducts>

<kineticLaw formula="c127*s063*s005/vol">

<listOfParameters>

<parameter name="c127" value="1e-018"/>

<parameter name="vol" value="5e-016"/>

</listOfParameters>

</kineticLaw>

</reaction>

<reaction name="r128" reversible="false">

<listOfReactants>

<specieReference specie="s178"/>

</listOfReactants>

<listOfProducts>

<specieReference specie="s063"/>

<specieReference specie="s005"/>

</listOfProducts>

<kineticLaw formula="c128*s178">

<listOfParameters>

<parameter name="c128" value="0.1"/>

</listOfParameters>

</kineticLaw>

</reaction>

<reaction name="r129" reversible="false">

<listOfReactants>

<specieReference specie="s178"/>

<specieReference specie="s043"/>

</listOfReactants>

<listOfProducts>

<specieReference specie="s179"/>

</listOfProducts>

<kineticLaw formula="c129*s178*s043/vol">

<listOfParameters>

<parameter name="c129" value="1e-018"/>

<parameter name="vol" value="5e-016"/>

</listOfParameters>

</kineticLaw>

</reaction>

<reaction name="r130" reversible="false">

<listOfReactants>

<specieReference specie="s179"/>

</listOfReactants>

<listOfProducts>

<specieReference specie="s178"/>

<specieReference specie="s043"/>

</listOfProducts>

<kineticLaw formula="c130*s179">

<listOfParameters>

<parameter name="c130" value="0.1"/>

</listOfParameters>

</kineticLaw>

</reaction>

<reaction name="r131" reversible="false">

<listOfReactants>

<specieReference specie="s179"/>

</listOfReactants>

<listOfProducts>

<specieReference specie="s180"/>

</listOfProducts>

<kineticLaw formula="c131*s179">

<listOfParameters>

<parameter name="c131" value="0.1"/>

</listOfParameters>

</kineticLaw>

</reaction>

<reaction name="r132" reversible="false">

<listOfReactants>

<specieReference specie="s180"/>

</listOfReactants>

<listOfProducts>

<specieReference specie="s181"/>

<specieReference specie="s021"/>

</listOfProducts>

<kineticLaw formula="c132*s180">

<listOfParameters>

<parameter name="c132" value="0.1"/>

</listOfParameters>

</kineticLaw>

</reaction>

<reaction name="r133" reversible="false">

<listOfReactants>

<specieReference specie="s181"/>

<specieReference specie="s083"/>

</listOfReactants>

<listOfProducts>

<specieReference specie="s182"/>

</listOfProducts>

<kineticLaw formula="c133*s181*s083/vol">

<listOfParameters>

<parameter name="c133" value="1e-018"/>

<parameter name="vol" value="5e-016"/>

</listOfParameters>

</kineticLaw>

</reaction>

<reaction name="r134" reversible="false">

<listOfReactants>

<specieReference specie="s182"/>

</listOfReactants>

<listOfProducts>

<specieReference specie="s181"/>

<specieReference specie="s083"/>

</listOfProducts>

<kineticLaw formula="c134*s182">

<listOfParameters>

<parameter name="c134" value="0.1"/>

</listOfParameters>

</kineticLaw>

</reaction>

<reaction name="r135" reversible="false">

<listOfReactants>

<specieReference specie="s182"/>

</listOfReactants>

<listOfProducts>

<specieReference specie="s183"/>

<specieReference specie="s020"/>

</listOfProducts>

<kineticLaw formula="c135*s182">

<listOfParameters>

<parameter name="c135" value="0.1"/>

</listOfParameters>

</kineticLaw>

</reaction>

<reaction name="r136" reversible="false">

<listOfReactants>

<specieReference specie="s183"/>

</listOfReactants>

<listOfProducts>

<specieReference specie="s103"/>

<specieReference specie="s063"/>

</listOfProducts>

<kineticLaw formula="c136*s183">

<listOfParameters>

<parameter name="c136" value="0.1"/>

</listOfParameters>

</kineticLaw>

</reaction>

<reaction name="r137" reversible="false">

<listOfReactants>

<specieReference specie="s064"/>

<specieReference specie="s005"/>

</listOfReactants>

<listOfProducts>

<specieReference specie="s184"/>

</listOfProducts>

<kineticLaw formula="c137*s064*s005/vol">

<listOfParameters>

<parameter name="c137" value="1e-018"/>

<parameter name="vol" value="5e-016"/>

</listOfParameters>

</kineticLaw>

</reaction>

<reaction name="r138" reversible="false">

<listOfReactants>

<specieReference specie="s184"/>

</listOfReactants>

<listOfProducts>

<specieReference specie="s064"/>

<specieReference specie="s005"/>

</listOfProducts>

<kineticLaw formula="c138*s184">

<listOfParameters>

<parameter name="c138" value="0.1"/>

</listOfParameters>

</kineticLaw>

</reaction>

<reaction name="r139" reversible="false">

<listOfReactants>

<specieReference specie="s184"/>

<specieReference specie="s044"/>

</listOfReactants>

<listOfProducts>

<specieReference specie="s185"/>

</listOfProducts>

<kineticLaw formula="c139*s184*s044/vol">

<listOfParameters>

<parameter name="c139" value="1e-018"/>

<parameter name="vol" value="5e-016"/>

</listOfParameters>

</kineticLaw>

</reaction>

<reaction name="r140" reversible="false">

<listOfReactants>

<specieReference specie="s185"/>

</listOfReactants>

<listOfProducts>

<specieReference specie="s184"/>

<specieReference specie="s044"/>

</listOfProducts>

<kineticLaw formula="c140*s185">

<listOfParameters>

<parameter name="c140" value="0.1"/>

</listOfParameters>

</kineticLaw>

</reaction>

<reaction name="r141" reversible="false">

<listOfReactants>

<specieReference specie="s185"/>

</listOfReactants>

<listOfProducts>

<specieReference specie="s186"/>

</listOfProducts>

<kineticLaw formula="c141*s185">

<listOfParameters>

<parameter name="c141" value="0.1"/>

</listOfParameters>

</kineticLaw>

</reaction>

<reaction name="r142" reversible="false">

<listOfReactants>

<specieReference specie="s186"/>

</listOfReactants>

<listOfProducts>

<specieReference specie="s187"/>

<specieReference specie="s021"/>

</listOfProducts>

<kineticLaw formula="c142*s186">

<listOfParameters>

<parameter name="c142" value="0.1"/>

</listOfParameters>

</kineticLaw>

</reaction>

<reaction name="r143" reversible="false">

<listOfReactants>

<specieReference specie="s187"/>

<specieReference specie="s084"/>

</listOfReactants>

<listOfProducts>

<specieReference specie="s188"/>

</listOfProducts>

<kineticLaw formula="c143*s187*s084/vol">

<listOfParameters>

<parameter name="c143" value="1e-018"/>

<parameter name="vol" value="5e-016"/>

</listOfParameters>

</kineticLaw>

</reaction>

<reaction name="r144" reversible="false">

<listOfReactants>

<specieReference specie="s188"/>

</listOfReactants>

<listOfProducts>

<specieReference specie="s187"/>

<specieReference specie="s084"/>

</listOfProducts>

<kineticLaw formula="c144*s188">

<listOfParameters>

<parameter name="c144" value="0.1"/>

</listOfParameters>

</kineticLaw>

</reaction>

<reaction name="r145" reversible="false">

<listOfReactants>

<specieReference specie="s188"/>

</listOfReactants>

<listOfProducts>

<specieReference specie="s189"/>

<specieReference specie="s020"/>

</listOfProducts>

<kineticLaw formula="c145*s188">

<listOfParameters>

<parameter name="c145" value="0.1"/>

</listOfParameters>

</kineticLaw>

</reaction>

<reaction name="r146" reversible="false">

<listOfReactants>

<specieReference specie="s189"/>

</listOfReactants>

<listOfProducts>

<specieReference specie="s104"/>

<specieReference specie="s064"/>

</listOfProducts>

<kineticLaw formula="c146*s189">

<listOfParameters>

<parameter name="c146" value="0.1"/>

</listOfParameters>

</kineticLaw>

</reaction>

<reaction name="r147" reversible="false">

<listOfReactants>

<specieReference specie="s065"/>

<specieReference specie="s005"/>

</listOfReactants>

<listOfProducts>

<specieReference specie="s190"/>

</listOfProducts>

<kineticLaw formula="c147*s065*s005/vol">

<listOfParameters>

<parameter name="c147" value="1e-018"/>

<parameter name="vol" value="5e-016"/>

</listOfParameters>

</kineticLaw>

</reaction>

<reaction name="r148" reversible="false">

<listOfReactants>

<specieReference specie="s190"/>

</listOfReactants>

<listOfProducts>

<specieReference specie="s065"/>

<specieReference specie="s005"/>

</listOfProducts>

<kineticLaw formula="c148*s190">

<listOfParameters>

<parameter name="c148" value="0.1"/>

</listOfParameters>

</kineticLaw>

</reaction>

<reaction name="r149" reversible="false">

<listOfReactants>

<specieReference specie="s190"/>

<specieReference specie="s045"/>

</listOfReactants>

<listOfProducts>

<specieReference specie="s191"/>

</listOfProducts>

<kineticLaw formula="c149*s190*s045/vol">

<listOfParameters>

<parameter name="c149" value="1e-018"/>

<parameter name="vol" value="5e-016"/>

</listOfParameters>

</kineticLaw>

</reaction>

<reaction name="r150" reversible="false">

<listOfReactants>

<specieReference specie="s191"/>

</listOfReactants>

<listOfProducts>

<specieReference specie="s190"/>

<specieReference specie="s045"/>

</listOfProducts>

<kineticLaw formula="c150*s191">

<listOfParameters>

<parameter name="c150" value="0.1"/>

</listOfParameters>

</kineticLaw>

</reaction>

<reaction name="r151" reversible="false">

<listOfReactants>

<specieReference specie="s191"/>

</listOfReactants>

<listOfProducts>

<specieReference specie="s192"/>

</listOfProducts>

<kineticLaw formula="c151*s191">

<listOfParameters>

<parameter name="c151" value="0.1"/>

</listOfParameters>

</kineticLaw>

</reaction>

<reaction name="r152" reversible="false">

<listOfReactants>

<specieReference specie="s192"/>

</listOfReactants>

<listOfProducts>

<specieReference specie="s193"/>

<specieReference specie="s021"/>

</listOfProducts>

<kineticLaw formula="c152*s192">

<listOfParameters>

<parameter name="c152" value="0.1"/>

</listOfParameters>

</kineticLaw>

</reaction>

<reaction name="r153" reversible="false">

<listOfReactants>

<specieReference specie="s193"/>

<specieReference specie="s085"/>

</listOfReactants>

<listOfProducts>

<specieReference specie="s194"/>

</listOfProducts>

<kineticLaw formula="c153*s193*s085/vol">

<listOfParameters>

<parameter name="c153" value="1e-018"/>

<parameter name="vol" value="5e-016"/>

</listOfParameters>

</kineticLaw>

</reaction>

<reaction name="r154" reversible="false">

<listOfReactants>

<specieReference specie="s194"/>

</listOfReactants>

<listOfProducts>

<specieReference specie="s193"/>

<specieReference specie="s085"/>

</listOfProducts>

<kineticLaw formula="c154*s194">

<listOfParameters>

<parameter name="c154" value="0.1"/>

</listOfParameters>

</kineticLaw>

</reaction>

<reaction name="r155" reversible="false">

<listOfReactants>

<specieReference specie="s194"/>

</listOfReactants>

<listOfProducts>

<specieReference specie="s195"/>

<specieReference specie="s020"/>

</listOfProducts>

<kineticLaw formula="c155*s194">

<listOfParameters>

<parameter name="c155" value="0.1"/>

</listOfParameters>

</kineticLaw>

</reaction>

<reaction name="r156" reversible="false">

<listOfReactants>

<specieReference specie="s195"/>

</listOfReactants>

<listOfProducts>

<specieReference specie="s105"/>

<specieReference specie="s065"/>

</listOfProducts>

<kineticLaw formula="c156*s195">

<listOfParameters>

<parameter name="c156" value="0.1"/>

</listOfParameters>

</kineticLaw>

</reaction>

<reaction name="r157" reversible="false">

<listOfReactants>

<specieReference specie="s066"/>

<specieReference specie="s005"/>

</listOfReactants>

<listOfProducts>

<specieReference specie="s196"/>

</listOfProducts>

<kineticLaw formula="c157*s066*s005/vol">

<listOfParameters>

<parameter name="c157" value="1e-018"/>

<parameter name="vol" value="5e-016"/>

</listOfParameters>

</kineticLaw>

</reaction>

<reaction name="r158" reversible="false">

<listOfReactants>

<specieReference specie="s196"/>

</listOfReactants>

<listOfProducts>

<specieReference specie="s066"/>

<specieReference specie="s005"/>

</listOfProducts>

<kineticLaw formula="c158*s196">

<listOfParameters>

<parameter name="c158" value="0.1"/>

</listOfParameters>

</kineticLaw>

</reaction>

<reaction name="r159" reversible="false">

<listOfReactants>

<specieReference specie="s196"/>

<specieReference specie="s046"/>

</listOfReactants>

<listOfProducts>

<specieReference specie="s197"/>

</listOfProducts>

<kineticLaw formula="c159*s196*s046/vol">

<listOfParameters>

<parameter name="c159" value="1e-018"/>

<parameter name="vol" value="5e-016"/>

</listOfParameters>

</kineticLaw>

</reaction>

<reaction name="r160" reversible="false">

<listOfReactants>

<specieReference specie="s197"/>

</listOfReactants>

<listOfProducts>

<specieReference specie="s196"/>

<specieReference specie="s046"/>

</listOfProducts>

<kineticLaw formula="c160*s197">

<listOfParameters>

<parameter name="c160" value="0.1"/>

</listOfParameters>

</kineticLaw>

</reaction>

<reaction name="r161" reversible="false">

<listOfReactants>

<specieReference specie="s197"/>

</listOfReactants>

<listOfProducts>

<specieReference specie="s198"/>

</listOfProducts>

<kineticLaw formula="c161*s197">

<listOfParameters>

<parameter name="c161" value="0.1"/>

</listOfParameters>

</kineticLaw>

</reaction>

<reaction name="r162" reversible="false">

<listOfReactants>

<specieReference specie="s198"/>

</listOfReactants>

<listOfProducts>

<specieReference specie="s199"/>

<specieReference specie="s021"/>

</listOfProducts>

<kineticLaw formula="c162*s198">

<listOfParameters>

<parameter name="c162" value="0.1"/>

</listOfParameters>

</kineticLaw>

</reaction>

<reaction name="r163" reversible="false">

<listOfReactants>

<specieReference specie="s199"/>

<specieReference specie="s086"/>

</listOfReactants>

<listOfProducts>

<specieReference specie="s200"/>

</listOfProducts>

<kineticLaw formula="c163*s199*s086/vol">

<listOfParameters>

<parameter name="c163" value="1e-018"/>

<parameter name="vol" value="5e-016"/>

</listOfParameters>

</kineticLaw>

</reaction>

<reaction name="r164" reversible="false">

<listOfReactants>

<specieReference specie="s200"/>

</listOfReactants>

<listOfProducts>

<specieReference specie="s199"/>

<specieReference specie="s086"/>

</listOfProducts>

<kineticLaw formula="c164*s200">

<listOfParameters>

<parameter name="c164" value="0.1"/>

</listOfParameters>

</kineticLaw>

</reaction>

<reaction name="r165" reversible="false">

<listOfReactants>

<specieReference specie="s200"/>

</listOfReactants>

<listOfProducts>

<specieReference specie="s201"/>

<specieReference specie="s020"/>

</listOfProducts>

<kineticLaw formula="c165*s200">

<listOfParameters>

<parameter name="c165" value="0.1"/>

</listOfParameters>

</kineticLaw>

</reaction>

<reaction name="r166" reversible="false">

<listOfReactants>

<specieReference specie="s201"/>

</listOfReactants>

<listOfProducts>

<specieReference specie="s106"/>

<specieReference specie="s066"/>

</listOfProducts>

<kineticLaw formula="c166*s201">

<listOfParameters>

<parameter name="c166" value="0.1"/>

</listOfParameters>

</kineticLaw>

</reaction>

<reaction name="r167" reversible="false">

<listOfReactants>

<specieReference specie="s067"/>

<specieReference specie="s005"/>

</listOfReactants>

<listOfProducts>

<specieReference specie="s202"/>

</listOfProducts>

<kineticLaw formula="c167*s067*s005/vol">

<listOfParameters>

<parameter name="c167" value="1e-018"/>

<parameter name="vol" value="5e-016"/>

</listOfParameters>

</kineticLaw>

</reaction>

<reaction name="r168" reversible="false">

<listOfReactants>

<specieReference specie="s202"/>

</listOfReactants>

<listOfProducts>

<specieReference specie="s067"/>

<specieReference specie="s005"/>

</listOfProducts>

<kineticLaw formula="c168*s202">

<listOfParameters>

<parameter name="c168" value="0.1"/>

</listOfParameters>

</kineticLaw>

</reaction>

<reaction name="r169" reversible="false">

<listOfReactants>

<specieReference specie="s202"/>

<specieReference specie="s047"/>

</listOfReactants>

<listOfProducts>

<specieReference specie="s203"/>

</listOfProducts>

<kineticLaw formula="c169*s202*s047/vol">

<listOfParameters>

<parameter name="c169" value="1e-018"/>

<parameter name="vol" value="5e-016"/>

</listOfParameters>

</kineticLaw>

</reaction>

<reaction name="r170" reversible="false">

<listOfReactants>

<specieReference specie="s203"/>

</listOfReactants>

<listOfProducts>

<specieReference specie="s202"/>

<specieReference specie="s047"/>

</listOfProducts>

<kineticLaw formula="c170*s203">

<listOfParameters>

<parameter name="c170" value="0.1"/>

</listOfParameters>

</kineticLaw>

</reaction>

<reaction name="r171" reversible="false">

<listOfReactants>

<specieReference specie="s203"/>

</listOfReactants>

<listOfProducts>

<specieReference specie="s204"/>

</listOfProducts>

<kineticLaw formula="c171*s203">

<listOfParameters>

<parameter name="c171" value="0.1"/>

</listOfParameters>

</kineticLaw>

</reaction>

<reaction name="r172" reversible="false">

<listOfReactants>

<specieReference specie="s204"/>

</listOfReactants>

<listOfProducts>

<specieReference specie="s205"/>

<specieReference specie="s021"/>

</listOfProducts>

<kineticLaw formula="c172*s204">

<listOfParameters>

<parameter name="c172" value="0.1"/>

</listOfParameters>

</kineticLaw>

</reaction>

<reaction name="r173" reversible="false">

<listOfReactants>

<specieReference specie="s205"/>

<specieReference specie="s087"/>

</listOfReactants>

<listOfProducts>

<specieReference specie="s206"/>

</listOfProducts>

<kineticLaw formula="c173*s205*s087/vol">

<listOfParameters>

<parameter name="c173" value="1e-018"/>

<parameter name="vol" value="5e-016"/>

</listOfParameters>

</kineticLaw>

</reaction>

<reaction name="r174" reversible="false">

<listOfReactants>

<specieReference specie="s206"/>

</listOfReactants>

<listOfProducts>

<specieReference specie="s205"/>

<specieReference specie="s087"/>

</listOfProducts>

<kineticLaw formula="c174*s206">

<listOfParameters>

<parameter name="c174" value="0.1"/>

</listOfParameters>

</kineticLaw>

</reaction>

<reaction name="r175" reversible="false">

<listOfReactants>

<specieReference specie="s206"/>

</listOfReactants>

<listOfProducts>

<specieReference specie="s207"/>

<specieReference specie="s020"/>

</listOfProducts>

<kineticLaw formula="c175*s206">

<listOfParameters>

<parameter name="c175" value="0.1"/>

</listOfParameters>

</kineticLaw>

</reaction>

<reaction name="r176" reversible="false">

<listOfReactants>

<specieReference specie="s207"/>

</listOfReactants>

<listOfProducts>

<specieReference specie="s107"/>

<specieReference specie="s067"/>

</listOfProducts>

<kineticLaw formula="c176*s207">

<listOfParameters>

<parameter name="c176" value="0.1"/>

</listOfParameters>

</kineticLaw>

</reaction>

<reaction name="r177" reversible="false">

<listOfReactants>

<specieReference specie="s068"/>

<specieReference specie="s005"/>

</listOfReactants>

<listOfProducts>

<specieReference specie="s208"/>

</listOfProducts>

<kineticLaw formula="c177*s068*s005/vol">

<listOfParameters>

<parameter name="c177" value="1e-018"/>

<parameter name="vol" value="5e-016"/>

</listOfParameters>

</kineticLaw>

</reaction>

<reaction name="r178" reversible="false">

<listOfReactants>

<specieReference specie="s208"/>

</listOfReactants>

<listOfProducts>

<specieReference specie="s068"/>

<specieReference specie="s005"/>

</listOfProducts>

<kineticLaw formula="c178*s208">

<listOfParameters>

<parameter name="c178" value="0.1"/>

</listOfParameters>

</kineticLaw>

</reaction>

<reaction name="r179" reversible="false">

<listOfReactants>

<specieReference specie="s208"/>

<specieReference specie="s048"/>

</listOfReactants>

<listOfProducts>

<specieReference specie="s209"/>

</listOfProducts>

<kineticLaw formula="c179*s208*s048/vol">

<listOfParameters>

<parameter name="c179" value="1e-018"/>

<parameter name="vol" value="5e-016"/>

</listOfParameters>

</kineticLaw>

</reaction>

<reaction name="r180" reversible="false">

<listOfReactants>

<specieReference specie="s209"/>

</listOfReactants>

<listOfProducts>

<specieReference specie="s208"/>

<specieReference specie="s048"/>

</listOfProducts>

<kineticLaw formula="c180*s209">

<listOfParameters>

<parameter name="c180" value="0.1"/>

</listOfParameters>

</kineticLaw>

</reaction>

<reaction name="r181" reversible="false">

<listOfReactants>

<specieReference specie="s209"/>

</listOfReactants>

<listOfProducts>

<specieReference specie="s210"/>

</listOfProducts>

<kineticLaw formula="c181*s209">

<listOfParameters>

<parameter name="c181" value="0.1"/>

</listOfParameters>

</kineticLaw>

</reaction>

<reaction name="r182" reversible="false">

<listOfReactants>

<specieReference specie="s210"/>

</listOfReactants>

<listOfProducts>

<specieReference specie="s211"/>

<specieReference specie="s021"/>

</listOfProducts>

<kineticLaw formula="c182*s210">

<listOfParameters>

<parameter name="c182" value="0.1"/>

</listOfParameters>

</kineticLaw>

</reaction>

<reaction name="r183" reversible="false">

<listOfReactants>

<specieReference specie="s211"/>

<specieReference specie="s088"/>

</listOfReactants>

<listOfProducts>

<specieReference specie="s212"/>

</listOfProducts>

<kineticLaw formula="c183*s211*s088/vol">

<listOfParameters>

<parameter name="c183" value="1e-018"/>

<parameter name="vol" value="5e-016"/>

</listOfParameters>

</kineticLaw>

</reaction>

<reaction name="r184" reversible="false">

<listOfReactants>

<specieReference specie="s212"/>

</listOfReactants>

<listOfProducts>

<specieReference specie="s211"/>

<specieReference specie="s088"/>

</listOfProducts>

<kineticLaw formula="c184*s212">

<listOfParameters>

<parameter name="c184" value="0.1"/>

</listOfParameters>

</kineticLaw>

</reaction>

<reaction name="r185" reversible="false">

<listOfReactants>

<specieReference specie="s212"/>

</listOfReactants>

<listOfProducts>

<specieReference specie="s213"/>

<specieReference specie="s020"/>

</listOfProducts>

<kineticLaw formula="c185*s212">

<listOfParameters>

<parameter name="c185" value="0.1"/>

</listOfParameters>

</kineticLaw>

</reaction>

<reaction name="r186" reversible="false">

<listOfReactants>

<specieReference specie="s213"/>

</listOfReactants>

<listOfProducts>

<specieReference specie="s108"/>

<specieReference specie="s068"/>

</listOfProducts>

<kineticLaw formula="c186*s213">

<listOfParameters>

<parameter name="c186" value="0.1"/>

</listOfParameters>

</kineticLaw>

</reaction>

<reaction name="r187" reversible="false">

<listOfReactants>

<specieReference specie="s069"/>

<specieReference specie="s005"/>

</listOfReactants>

<listOfProducts>

<specieReference specie="s214"/>

</listOfProducts>

<kineticLaw formula="c187*s069*s005/vol">

<listOfParameters>

<parameter name="c187" value="1e-018"/>

<parameter name="vol" value="5e-016"/>

</listOfParameters>

</kineticLaw>

</reaction>

<reaction name="r188" reversible="false">

<listOfReactants>

<specieReference specie="s214"/>

</listOfReactants>

<listOfProducts>

<specieReference specie="s069"/>

<specieReference specie="s005"/>

</listOfProducts>

<kineticLaw formula="c188*s214">

<listOfParameters>

<parameter name="c188" value="0.1"/>

</listOfParameters>

</kineticLaw>

</reaction>

<reaction name="r189" reversible="false">

<listOfReactants>

<specieReference specie="s214"/>

<specieReference specie="s049"/>

</listOfReactants>

<listOfProducts>

<specieReference specie="s215"/>

</listOfProducts>

<kineticLaw formula="c189*s214*s049/vol">

<listOfParameters>

<parameter name="c189" value="1e-018"/>

<parameter name="vol" value="5e-016"/>

</listOfParameters>

</kineticLaw>

</reaction>

<reaction name="r190" reversible="false">

<listOfReactants>

<specieReference specie="s215"/>

</listOfReactants>

<listOfProducts>

<specieReference specie="s214"/>

<specieReference specie="s049"/>

</listOfProducts>

<kineticLaw formula="c190*s215">

<listOfParameters>

<parameter name="c190" value="0.1"/>

</listOfParameters>

</kineticLaw>

</reaction>

<reaction name="r191" reversible="false">

<listOfReactants>

<specieReference specie="s215"/>

</listOfReactants>

<listOfProducts>

<specieReference specie="s216"/>

</listOfProducts>

<kineticLaw formula="c191*s215">

<listOfParameters>

<parameter name="c191" value="0.1"/>

</listOfParameters>

</kineticLaw>

</reaction>

<reaction name="r192" reversible="false">

<listOfReactants>

<specieReference specie="s216"/>

</listOfReactants>

<listOfProducts>

<specieReference specie="s217"/>

<specieReference specie="s021"/>

</listOfProducts>

<kineticLaw formula="c192*s216">

<listOfParameters>

<parameter name="c192" value="0.1"/>

</listOfParameters>

</kineticLaw>

</reaction>

<reaction name="r193" reversible="false">

<listOfReactants>

<specieReference specie="s217"/>

<specieReference specie="s089"/>

</listOfReactants>

<listOfProducts>

<specieReference specie="s218"/>

</listOfProducts>

<kineticLaw formula="c193*s217*s089/vol">

<listOfParameters>

<parameter name="c193" value="1e-018"/>

<parameter name="vol" value="5e-016"/>

</listOfParameters>

</kineticLaw>

</reaction>

<reaction name="r194" reversible="false">

<listOfReactants>

<specieReference specie="s218"/>

</listOfReactants>

<listOfProducts>

<specieReference specie="s217"/>

<specieReference specie="s089"/>

</listOfProducts>

<kineticLaw formula="c194*s218">

<listOfParameters>

<parameter name="c194" value="0.1"/>

</listOfParameters>

</kineticLaw>

</reaction>

<reaction name="r195" reversible="false">

<listOfReactants>

<specieReference specie="s218"/>

</listOfReactants>

<listOfProducts>

<specieReference specie="s219"/>

<specieReference specie="s020"/>

</listOfProducts>

<kineticLaw formula="c195*s218">

<listOfParameters>

<parameter name="c195" value="0.1"/>

</listOfParameters>

</kineticLaw>

</reaction>

<reaction name="r196" reversible="false">

<listOfReactants>

<specieReference specie="s219"/>

</listOfReactants>

<listOfProducts>

<specieReference specie="s109"/>

<specieReference specie="s069"/>

</listOfProducts>

<kineticLaw formula="c196*s219">

<listOfParameters>

<parameter name="c196" value="0.1"/>

</listOfParameters>

</kineticLaw>

</reaction>

<reaction name="r197" reversible="false">

<listOfReactants>

<specieReference specie="s070"/>

<specieReference specie="s005"/>

</listOfReactants>

<listOfProducts>

<specieReference specie="s220"/>

</listOfProducts>

<kineticLaw formula="c197*s070*s005/vol">

<listOfParameters>

<parameter name="c197" value="1e-018"/>

<parameter name="vol" value="5e-016"/>

</listOfParameters>

</kineticLaw>

</reaction>

<reaction name="r198" reversible="false">

<listOfReactants>

<specieReference specie="s220"/>

</listOfReactants>

<listOfProducts>

<specieReference specie="s070"/>

<specieReference specie="s005"/>

</listOfProducts>

<kineticLaw formula="c198*s220">

<listOfParameters>

<parameter name="c198" value="0.1"/>

</listOfParameters>

</kineticLaw>

</reaction>

<reaction name="r199" reversible="false">

<listOfReactants>

<specieReference specie="s220"/>

<specieReference specie="s050"/>

</listOfReactants>

<listOfProducts>

<specieReference specie="s221"/>

</listOfProducts>

<kineticLaw formula="c199*s220*s050/vol">

<listOfParameters>

<parameter name="c199" value="1e-018"/>

<parameter name="vol" value="5e-016"/>

</listOfParameters>

</kineticLaw>

</reaction>

<reaction name="r200" reversible="false">

<listOfReactants>

<specieReference specie="s221"/>

</listOfReactants>

<listOfProducts>

<specieReference specie="s220"/>

<specieReference specie="s050"/>

</listOfProducts>

<kineticLaw formula="c200*s221">

<listOfParameters>

<parameter name="c200" value="0.1"/>

</listOfParameters>

</kineticLaw>

</reaction>

<reaction name="r201" reversible="false">

<listOfReactants>

<specieReference specie="s221"/>

</listOfReactants>

<listOfProducts>

<specieReference specie="s222"/>

</listOfProducts>

<kineticLaw formula="c201*s221">

<listOfParameters>

<parameter name="c201" value="0.1"/>

</listOfParameters>

</kineticLaw>

</reaction>

<reaction name="r202" reversible="false">

<listOfReactants>

<specieReference specie="s222"/>

</listOfReactants>

<listOfProducts>

<specieReference specie="s223"/>

<specieReference specie="s021"/>

</listOfProducts>

<kineticLaw formula="c202*s222">

<listOfParameters>

<parameter name="c202" value="0.1"/>

</listOfParameters>

</kineticLaw>

</reaction>

<reaction name="r203" reversible="false">

<listOfReactants>

<specieReference specie="s223"/>

<specieReference specie="s090"/>

</listOfReactants>

<listOfProducts>

<specieReference specie="s224"/>

</listOfProducts>

<kineticLaw formula="c203*s223*s090/vol">

<listOfParameters>

<parameter name="c203" value="1e-018"/>

<parameter name="vol" value="5e-016"/>

</listOfParameters>

</kineticLaw>

</reaction>

<reaction name="r204" reversible="false">

<listOfReactants>

<specieReference specie="s224"/>

</listOfReactants>

<listOfProducts>

<specieReference specie="s223"/>

<specieReference specie="s090"/>

</listOfProducts>

<kineticLaw formula="c204*s224">

<listOfParameters>

<parameter name="c204" value="0.1"/>

</listOfParameters>

</kineticLaw>

</reaction>

<reaction name="r205" reversible="false">

<listOfReactants>

<specieReference specie="s224"/>

</listOfReactants>

<listOfProducts>

<specieReference specie="s225"/>

<specieReference specie="s020"/>

</listOfProducts>

<kineticLaw formula="c205*s224">

<listOfParameters>

<parameter name="c205" value="0.1"/>

</listOfParameters>

</kineticLaw>

</reaction>

<reaction name="r206" reversible="false">

<listOfReactants>

<specieReference specie="s225"/>

</listOfReactants>

<listOfProducts>

<specieReference specie="s110"/>

<specieReference specie="s070"/>

</listOfProducts>

<kineticLaw formula="c206*s225">

<listOfParameters>

<parameter name="c206" value="0.1"/>

</listOfParameters>

</kineticLaw>

</reaction>

<reaction name="r207" reversible="false">

<listOfReactants>

<specieReference specie="s071"/>

<specieReference specie="s005"/>

</listOfReactants>

<listOfProducts>

<specieReference specie="s226"/>

</listOfProducts>

<kineticLaw formula="c207*s071*s005/vol">

<listOfParameters>

<parameter name="c207" value="1e-018"/>

<parameter name="vol" value="5e-016"/>

</listOfParameters>

</kineticLaw>

</reaction>

<reaction name="r208" reversible="false">

<listOfReactants>

<specieReference specie="s226"/>

</listOfReactants>

<listOfProducts>

<specieReference specie="s071"/>

<specieReference specie="s005"/>

</listOfProducts>

<kineticLaw formula="c208*s226">

<listOfParameters>

<parameter name="c208" value="0.1"/>

</listOfParameters>

</kineticLaw>

</reaction>

<reaction name="r209" reversible="false">

<listOfReactants>

<specieReference specie="s226"/>

<specieReference specie="s051"/>

</listOfReactants>

<listOfProducts>

<specieReference specie="s227"/>

</listOfProducts>

<kineticLaw formula="c209*s226*s051/vol">

<listOfParameters>

<parameter name="c209" value="1e-018"/>

<parameter name="vol" value="5e-016"/>

</listOfParameters>

</kineticLaw>

</reaction>

<reaction name="r210" reversible="false">

<listOfReactants>

<specieReference specie="s227"/>

</listOfReactants>

<listOfProducts>

<specieReference specie="s226"/>

<specieReference specie="s051"/>

</listOfProducts>

<kineticLaw formula="c210*s227">

<listOfParameters>

<parameter name="c210" value="0.1"/>

</listOfParameters>

</kineticLaw>

</reaction>

<reaction name="r211" reversible="false">

<listOfReactants>

<specieReference specie="s227"/>

</listOfReactants>

<listOfProducts>

<specieReference specie="s228"/>

</listOfProducts>

<kineticLaw formula="c211*s227">

<listOfParameters>

<parameter name="c211" value="0.1"/>

</listOfParameters>

</kineticLaw>

</reaction>

<reaction name="r212" reversible="false">

<listOfReactants>

<specieReference specie="s228"/>

</listOfReactants>

<listOfProducts>

<specieReference specie="s229"/>

<specieReference specie="s021"/>

</listOfProducts>

<kineticLaw formula="c212*s228">

<listOfParameters>

<parameter name="c212" value="0.1"/>

</listOfParameters>

</kineticLaw>

</reaction>

<reaction name="r213" reversible="false">

<listOfReactants>

<specieReference specie="s229"/>

<specieReference specie="s091"/>

</listOfReactants>

<listOfProducts>

<specieReference specie="s230"/>

</listOfProducts>

<kineticLaw formula="c213*s229*s091/vol">

<listOfParameters>

<parameter name="c213" value="1e-018"/>

<parameter name="vol" value="5e-016"/>

</listOfParameters>

</kineticLaw>

</reaction>

<reaction name="r214" reversible="false">

<listOfReactants>

<specieReference specie="s230"/>

</listOfReactants>

<listOfProducts>

<specieReference specie="s229"/>

<specieReference specie="s091"/>

</listOfProducts>

<kineticLaw formula="c214*s230">

<listOfParameters>

<parameter name="c214" value="0.1"/>

</listOfParameters>

</kineticLaw>

</reaction>

<reaction name="r215" reversible="false">

<listOfReactants>

<specieReference specie="s230"/>

</listOfReactants>

<listOfProducts>

<specieReference specie="s231"/>

<specieReference specie="s020"/>

</listOfProducts>

<kineticLaw formula="c215*s230">

<listOfParameters>

<parameter name="c215" value="0.1"/>

</listOfParameters>

</kineticLaw>

</reaction>

<reaction name="r216" reversible="false">

<listOfReactants>

<specieReference specie="s231"/>

</listOfReactants>

<listOfProducts>

<specieReference specie="s111"/>

<specieReference specie="s071"/>

</listOfProducts>

<kineticLaw formula="c216*s231">

<listOfParameters>

<parameter name="c216" value="0.1"/>

</listOfParameters>

</kineticLaw>

</reaction>

<reaction name="r217" reversible="false">

<listOfReactants>

<specieReference specie="s030"/>

<specieReference specie="s072"/>

</listOfReactants>

<listOfProducts>

<specieReference specie="s030"/>

<specieReference specie="s011" stoichiometry="25"/>

<specieReference specie="s012" stoichiometry="25"/>

<specieReference specie="s013" stoichiometry="25"/>

<specieReference specie="s014" stoichiometry="25"/>

</listOfProducts>

<kineticLaw formula="c217*a_s030*(s030/vol)*s072/(s072+km217_s072)">

<listOfParameters>

<parameter name="c217" value="5e-025"/>

<parameter name="a_s030" value="1"/>

<parameter name="km217_s072" value="301"/>

<parameter name="vol" value="5e-016"/>

</listOfParameters>

</kineticLaw>

</reaction>

<reaction name="r218" reversible="false">

<listOfReactants>

<specieReference specie="s030"/>

<specieReference specie="s073"/>

</listOfReactants>

<listOfProducts>

<specieReference specie="s030"/>

<specieReference specie="s011" stoichiometry="25"/>

<specieReference specie="s012" stoichiometry="25"/>

<specieReference specie="s013" stoichiometry="25"/>

<specieReference specie="s014" stoichiometry="25"/>

</listOfProducts>

<kineticLaw formula="c218*a_s030*(s030/vol)*s073/(s073+km218_s073)">

<listOfParameters>

<parameter name="c218" value="5e-025"/>

<parameter name="a_s030" value="1"/>

<parameter name="km218_s073" value="301"/>

<parameter name="vol" value="5e-016"/>

</listOfParameters>

</kineticLaw>

</reaction>

<reaction name="r219" reversible="false">

<listOfReactants>

<specieReference specie="s030"/>

<specieReference specie="s074"/>

</listOfReactants>

<listOfProducts>

<specieReference specie="s030"/>

<specieReference specie="s011" stoichiometry="25"/>

<specieReference specie="s012" stoichiometry="25"/>

<specieReference specie="s013" stoichiometry="25"/>

<specieReference specie="s014" stoichiometry="25"/>

</listOfProducts>

<kineticLaw formula="c219*a_s030*(s030/vol)*s074/(s074+km219_s074)">

<listOfParameters>

<parameter name="c219" value="5e-025"/>

<parameter name="a_s030" value="1"/>

<parameter name="km219_s074" value="301"/>

<parameter name="vol" value="5e-016"/>

</listOfParameters>

</kineticLaw>

</reaction>

<reaction name="r220" reversible="false">

<listOfReactants>

<specieReference specie="s030"/>

<specieReference specie="s075"/>

</listOfReactants>

<listOfProducts>

<specieReference specie="s030"/>

<specieReference specie="s011" stoichiometry="25"/>

<specieReference specie="s012" stoichiometry="25"/>

<specieReference specie="s013" stoichiometry="25"/>

<specieReference specie="s014" stoichiometry="25"/>

</listOfProducts>

<kineticLaw formula="c220*a_s030*(s030/vol)*s075/(s075+km220_s075)">

<listOfParameters>

<parameter name="c220" value="5e-025"/>

<parameter name="a_s030" value="1"/>

<parameter name="km220_s075" value="301"/>

<parameter name="vol" value="5e-016"/>

</listOfParameters>

</kineticLaw>

</reaction>

<reaction name="r221" reversible="false">

<listOfReactants>

<specieReference specie="s030"/>

<specieReference specie="s076"/>

</listOfReactants>

<listOfProducts>

<specieReference specie="s030"/>

<specieReference specie="s011" stoichiometry="25"/>

<specieReference specie="s012" stoichiometry="25"/>

<specieReference specie="s013" stoichiometry="25"/>

<specieReference specie="s014" stoichiometry="25"/>

</listOfProducts>

<kineticLaw formula="c221*a_s030*(s030/vol)*s076/(s076+km221_s076)">

<listOfParameters>

<parameter name="c221" value="5e-025"/>

<parameter name="a_s030" value="1"/>

<parameter name="km221_s076" value="301"/>

<parameter name="vol" value="5e-016"/>

</listOfParameters>

</kineticLaw>

</reaction>

<reaction name="r222" reversible="false">

<listOfReactants>

<specieReference specie="s030"/>

<specieReference specie="s077"/>

</listOfReactants>

<listOfProducts>

<specieReference specie="s030"/>

<specieReference specie="s011" stoichiometry="25"/>

<specieReference specie="s012" stoichiometry="25"/>

<specieReference specie="s013" stoichiometry="25"/>

<specieReference specie="s014" stoichiometry="25"/>

</listOfProducts>

<kineticLaw formula="c222*a_s030*(s030/vol)*s077/(s077+km222_s077)">

<listOfParameters>

<parameter name="c222" value="5e-025"/>

<parameter name="a_s030" value="1"/>

<parameter name="km222_s077" value="301"/>

<parameter name="vol" value="5e-016"/>

</listOfParameters>

</kineticLaw>

</reaction>

<reaction name="r223" reversible="false">

<listOfReactants>

<specieReference specie="s030"/>

<specieReference specie="s078"/>

</listOfReactants>

<listOfProducts>

<specieReference specie="s030"/>

<specieReference specie="s011" stoichiometry="25"/>

<specieReference specie="s012" stoichiometry="25"/>

<specieReference specie="s013" stoichiometry="25"/>

<specieReference specie="s014" stoichiometry="25"/>

</listOfProducts>

<kineticLaw formula="c223*a_s030*(s030/vol)*s078/(s078+km223_s078)">

<listOfParameters>

<parameter name="c223" value="5e-025"/>

<parameter name="a_s030" value="1"/>

<parameter name="km223_s078" value="301"/>

<parameter name="vol" value="5e-016"/>

</listOfParameters>

</kineticLaw>

</reaction>

<reaction name="r224" reversible="false">

<listOfReactants>

<specieReference specie="s030"/>

<specieReference specie="s079"/>

</listOfReactants>

<listOfProducts>

<specieReference specie="s030"/>

<specieReference specie="s011" stoichiometry="25"/>

<specieReference specie="s012" stoichiometry="25"/>

<specieReference specie="s013" stoichiometry="25"/>

<specieReference specie="s014" stoichiometry="25"/>

</listOfProducts>

<kineticLaw formula="c224*a_s030*(s030/vol)*s079/(s079+km224_s079)">

<listOfParameters>

<parameter name="c224" value="5e-025"/>

<parameter name="a_s030" value="1"/>

<parameter name="km224_s079" value="301"/>

<parameter name="vol" value="5e-016"/>

</listOfParameters>

</kineticLaw>

</reaction>

<reaction name="r225" reversible="false">

<listOfReactants>

<specieReference specie="s030"/>

<specieReference specie="s080"/>

</listOfReactants>

<listOfProducts>

<specieReference specie="s030"/>

<specieReference specie="s011" stoichiometry="25"/>

<specieReference specie="s012" stoichiometry="25"/>

<specieReference specie="s013" stoichiometry="25"/>

<specieReference specie="s014" stoichiometry="25"/>

</listOfProducts>

<kineticLaw formula="c225*a_s030*(s030/vol)*s080/(s080+km225_s080)">

<listOfParameters>

<parameter name="c225" value="5e-025"/>

<parameter name="a_s030" value="1"/>

<parameter name="km225_s080" value="301"/>

<parameter name="vol" value="5e-016"/>

</listOfParameters>

</kineticLaw>

</reaction>

<reaction name="r226" reversible="false">

<listOfReactants>

<specieReference specie="s030"/>

<specieReference specie="s081"/>

</listOfReactants>

<listOfProducts>

<specieReference specie="s030"/>

<specieReference specie="s011" stoichiometry="25"/>

<specieReference specie="s012" stoichiometry="25"/>

<specieReference specie="s013" stoichiometry="25"/>

<specieReference specie="s014" stoichiometry="25"/>

</listOfProducts>

<kineticLaw formula="c226*a_s030*(s030/vol)*s081/(s081+km226_s081)">

<listOfParameters>

<parameter name="c226" value="5e-025"/>

<parameter name="a_s030" value="1"/>

<parameter name="km226_s081" value="301"/>

<parameter name="vol" value="5e-016"/>

</listOfParameters>

</kineticLaw>

</reaction>

<reaction name="r227" reversible="false">

<listOfReactants>

<specieReference specie="s030"/>

<specieReference specie="s082"/>

</listOfReactants>

<listOfProducts>

<specieReference specie="s030"/>

<specieReference specie="s011" stoichiometry="25"/>

<specieReference specie="s012" stoichiometry="25"/>

<specieReference specie="s013" stoichiometry="25"/>

<specieReference specie="s014" stoichiometry="25"/>

</listOfProducts>

<kineticLaw formula="c227*a_s030*(s030/vol)*s082/(s082+km227_s082)">

<listOfParameters>

<parameter name="c227" value="5e-025"/>

<parameter name="a_s030" value="1"/>

<parameter name="km227_s082" value="301"/>

<parameter name="vol" value="5e-016"/>

</listOfParameters>

</kineticLaw>

</reaction>

<reaction name="r228" reversible="false">

<listOfReactants>

<specieReference specie="s030"/>

<specieReference specie="s083"/>

</listOfReactants>

<listOfProducts>

<specieReference specie="s030"/>

<specieReference specie="s011" stoichiometry="25"/>

<specieReference specie="s012" stoichiometry="25"/>

<specieReference specie="s013" stoichiometry="25"/>

<specieReference specie="s014" stoichiometry="25"/>

</listOfProducts>

<kineticLaw formula="c228*a_s030*(s030/vol)*s083/(s083+km228_s083)">

<listOfParameters>

<parameter name="c228" value="5e-025"/>

<parameter name="a_s030" value="1"/>

<parameter name="km228_s083" value="301"/>

<parameter name="vol" value="5e-016"/>

</listOfParameters>

</kineticLaw>

</reaction>

<reaction name="r229" reversible="false">

<listOfReactants>

<specieReference specie="s030"/>

<specieReference specie="s084"/>

</listOfReactants>

<listOfProducts>

<specieReference specie="s030"/>

<specieReference specie="s011" stoichiometry="25"/>

<specieReference specie="s012" stoichiometry="25"/>

<specieReference specie="s013" stoichiometry="25"/>

<specieReference specie="s014" stoichiometry="25"/>

</listOfProducts>

<kineticLaw formula="c229*a_s030*(s030/vol)*s084/(s084+km229_s084)">

<listOfParameters>

<parameter name="c229" value="5e-025"/>

<parameter name="a_s030" value="1"/>

<parameter name="km229_s084" value="301"/>

<parameter name="vol" value="5e-016"/>

</listOfParameters>

</kineticLaw>

</reaction>

<reaction name="r230" reversible="false">

<listOfReactants>

<specieReference specie="s030"/>

<specieReference specie="s085"/>

</listOfReactants>

<listOfProducts>

<specieReference specie="s030"/>

<specieReference specie="s011" stoichiometry="25"/>

<specieReference specie="s012" stoichiometry="25"/>

<specieReference specie="s013" stoichiometry="25"/>

<specieReference specie="s014" stoichiometry="25"/>

</listOfProducts>

<kineticLaw formula="c230*a_s030*(s030/vol)*s085/(s085+km230_s085)">

<listOfParameters>

<parameter name="c230" value="5e-025"/>

<parameter name="a_s030" value="1"/>

<parameter name="km230_s085" value="301"/>

<parameter name="vol" value="5e-016"/>

</listOfParameters>

</kineticLaw>

</reaction>

<reaction name="r231" reversible="false">

<listOfReactants>

<specieReference specie="s030"/>

<specieReference specie="s086"/>

</listOfReactants>

<listOfProducts>

<specieReference specie="s030"/>

<specieReference specie="s011" stoichiometry="25"/>

<specieReference specie="s012" stoichiometry="25"/>

<specieReference specie="s013" stoichiometry="25"/>

<specieReference specie="s014" stoichiometry="25"/>

</listOfProducts>

<kineticLaw formula="c231*a_s030*(s030/vol)*s086/(s086+km231_s086)">

<listOfParameters>

<parameter name="c231" value="5e-025"/>

<parameter name="a_s030" value="1"/>

<parameter name="km231_s086" value="301"/>

<parameter name="vol" value="5e-016"/>

</listOfParameters>

</kineticLaw>

</reaction>

<reaction name="r232" reversible="false">

<listOfReactants>

<specieReference specie="s030"/>

<specieReference specie="s087"/>

</listOfReactants>

<listOfProducts>

<specieReference specie="s030"/>

<specieReference specie="s011" stoichiometry="25"/>

<specieReference specie="s012" stoichiometry="25"/>

<specieReference specie="s013" stoichiometry="25"/>

<specieReference specie="s014" stoichiometry="25"/>

</listOfProducts>

<kineticLaw formula="c232*a_s030*(s030/vol)*s087/(s087+km232_s087)">

<listOfParameters>

<parameter name="c232" value="5e-025"/>

<parameter name="a_s030" value="1"/>

<parameter name="km232_s087" value="301"/>

<parameter name="vol" value="5e-016"/>

</listOfParameters>

</kineticLaw>

</reaction>

<reaction name="r233" reversible="false">

<listOfReactants>

<specieReference specie="s030"/>

<specieReference specie="s088"/>

</listOfReactants>

<listOfProducts>

<specieReference specie="s030"/>

<specieReference specie="s011" stoichiometry="25"/>

<specieReference specie="s012" stoichiometry="25"/>

<specieReference specie="s013" stoichiometry="25"/>

<specieReference specie="s014" stoichiometry="25"/>

</listOfProducts>

<kineticLaw formula="c233*a_s030*(s030/vol)*s088/(s088+km233_s088)">

<listOfParameters>

<parameter name="c233" value="5e-025"/>

<parameter name="a_s030" value="1"/>

<parameter name="km233_s088" value="301"/>

<parameter name="vol" value="5e-016"/>

</listOfParameters>

</kineticLaw>

</reaction>

<reaction name="r234" reversible="false">

<listOfReactants>

<specieReference specie="s030"/>

<specieReference specie="s089"/>

</listOfReactants>

<listOfProducts>

<specieReference specie="s030"/>

<specieReference specie="s011" stoichiometry="25"/>

<specieReference specie="s012" stoichiometry="25"/>

<specieReference specie="s013" stoichiometry="25"/>

<specieReference specie="s014" stoichiometry="25"/>

</listOfProducts>

<kineticLaw formula="c234*a_s030*(s030/vol)*s089/(s089+km234_s089)">

<listOfParameters>

<parameter name="c234" value="5e-025"/>

<parameter name="a_s030" value="1"/>

<parameter name="km234_s089" value="301"/>

<parameter name="vol" value="5e-016"/>

</listOfParameters>

</kineticLaw>

</reaction>

<reaction name="r235" reversible="false">

<listOfReactants>

<specieReference specie="s030"/>

<specieReference specie="s090"/>

</listOfReactants>

<listOfProducts>

<specieReference specie="s030"/>

<specieReference specie="s011" stoichiometry="25"/>

<specieReference specie="s012" stoichiometry="25"/>

<specieReference specie="s013" stoichiometry="25"/>

<specieReference specie="s014" stoichiometry="25"/>

</listOfProducts>

<kineticLaw formula="c235*a_s030*(s030/vol)*s090/(s090+km235_s090)">

<listOfParameters>

<parameter name="c235" value="5e-025"/>

<parameter name="a_s030" value="1"/>

<parameter name="km235_s090" value="301"/>

<parameter name="vol" value="5e-016"/>

</listOfParameters>

</kineticLaw>

</reaction>

<reaction name="r236" reversible="false">

<listOfReactants>

<specieReference specie="s030"/>

<specieReference specie="s091"/>

</listOfReactants>

<listOfProducts>

<specieReference specie="s030"/>

<specieReference specie="s011" stoichiometry="25"/>

<specieReference specie="s012" stoichiometry="25"/>

<specieReference specie="s013" stoichiometry="25"/>

<specieReference specie="s014" stoichiometry="25"/>

</listOfProducts>

<kineticLaw formula="c236*a_s030*(s030/vol)*s091/(s091+km236_s091)">

<listOfParameters>

<parameter name="c236" value="5e-025"/>

<parameter name="a_s030" value="1"/>

<parameter name="km236_s091" value="301"/>

<parameter name="vol" value="5e-016"/>

</listOfParameters>

</kineticLaw>

</reaction>

<reaction name="r237" reversible="false">

<listOfReactants>

<specieReference specie="s031"/>

<specieReference specie="s052"/>

</listOfReactants>

<listOfProducts>

<specieReference specie="s031"/>

<specieReference specie="s032" stoichiometry="20"/>

<specieReference specie="s033" stoichiometry="20"/>

<specieReference specie="s034" stoichiometry="20"/>

<specieReference specie="s035" stoichiometry="20"/>

<specieReference specie="s036" stoichiometry="20"/>

<specieReference specie="s037" stoichiometry="20"/>

<specieReference specie="s038" stoichiometry="20"/>

<specieReference specie="s039" stoichiometry="20"/>

<specieReference specie="s040" stoichiometry="20"/>

<specieReference specie="s041" stoichiometry="20"/>

<specieReference specie="s042" stoichiometry="20"/>

<specieReference specie="s043" stoichiometry="20"/>

<specieReference specie="s044" stoichiometry="20"/>

<specieReference specie="s045" stoichiometry="20"/>

<specieReference specie="s046" stoichiometry="20"/>

<specieReference specie="s047" stoichiometry="20"/>

<specieReference specie="s048" stoichiometry="20"/>

<specieReference specie="s049" stoichiometry="20"/>

<specieReference specie="s050" stoichiometry="20"/>

<specieReference specie="s051" stoichiometry="20"/>

</listOfProducts>

<kineticLaw formula="c237*a_s031*(s031/vol)*s052/(s052+km237_s052)">

<listOfParameters>

<parameter name="c237" value="5e-025"/>

<parameter name="a_s031" value="1"/>

<parameter name="km237_s052" value="301"/>

<parameter name="vol" value="5e-016"/>

</listOfParameters>

</kineticLaw>

</reaction>

<reaction name="r238" reversible="false">

<listOfReactants>

<specieReference specie="s031"/>

<specieReference specie="s053"/>

</listOfReactants>

<listOfProducts>

<specieReference specie="s031"/>

<specieReference specie="s032" stoichiometry="20"/>

<specieReference specie="s033" stoichiometry="20"/>

<specieReference specie="s034" stoichiometry="20"/>

<specieReference specie="s035" stoichiometry="20"/>

<specieReference specie="s036" stoichiometry="20"/>

<specieReference specie="s037" stoichiometry="20"/>

<specieReference specie="s038" stoichiometry="20"/>

<specieReference specie="s039" stoichiometry="20"/>

<specieReference specie="s040" stoichiometry="20"/>

<specieReference specie="s041" stoichiometry="20"/>

<specieReference specie="s042" stoichiometry="20"/>

<specieReference specie="s043" stoichiometry="20"/>

<specieReference specie="s044" stoichiometry="20"/>

<specieReference specie="s045" stoichiometry="20"/>

<specieReference specie="s046" stoichiometry="20"/>

<specieReference specie="s047" stoichiometry="20"/>

<specieReference specie="s048" stoichiometry="20"/>

<specieReference specie="s049" stoichiometry="20"/>

<specieReference specie="s050" stoichiometry="20"/>

<specieReference specie="s051" stoichiometry="20"/>

</listOfProducts>

<kineticLaw formula="c238*a_s031*(s031/vol)*s053/(s053+km238_s053)">

<listOfParameters>

<parameter name="c238" value="5e-025"/>

<parameter name="a_s031" value="1"/>

<parameter name="km238_s053" value="301"/>

<parameter name="vol" value="5e-016"/>

</listOfParameters>

</kineticLaw>

</reaction>

<reaction name="r239" reversible="false">

<listOfReactants>

<specieReference specie="s031"/>

<specieReference specie="s054"/>

</listOfReactants>

<listOfProducts>

<specieReference specie="s031"/>

<specieReference specie="s032" stoichiometry="20"/>

<specieReference specie="s033" stoichiometry="20"/>

<specieReference specie="s034" stoichiometry="20"/>

<specieReference specie="s035" stoichiometry="20"/>

<specieReference specie="s036" stoichiometry="20"/>

<specieReference specie="s037" stoichiometry="20"/>

<specieReference specie="s038" stoichiometry="20"/>

<specieReference specie="s039" stoichiometry="20"/>

<specieReference specie="s040" stoichiometry="20"/>

<specieReference specie="s041" stoichiometry="20"/>

<specieReference specie="s042" stoichiometry="20"/>

<specieReference specie="s043" stoichiometry="20"/>

<specieReference specie="s044" stoichiometry="20"/>

<specieReference specie="s045" stoichiometry="20"/>

<specieReference specie="s046" stoichiometry="20"/>

<specieReference specie="s047" stoichiometry="20"/>

<specieReference specie="s048" stoichiometry="20"/>

<specieReference specie="s049" stoichiometry="20"/>

<specieReference specie="s050" stoichiometry="20"/>

<specieReference specie="s051" stoichiometry="20"/>

</listOfProducts>

<kineticLaw formula="c239*a_s031*(s031/vol)*s054/(s054+km239_s054)">

<listOfParameters>

<parameter name="c239" value="5e-025"/>

<parameter name="a_s031" value="1"/>

<parameter name="km239_s054" value="301"/>

<parameter name="vol" value="5e-016"/>

</listOfParameters>

</kineticLaw>

</reaction>

<reaction name="r240" reversible="false">

<listOfReactants>

<specieReference specie="s031"/>

<specieReference specie="s055"/>

</listOfReactants>

<listOfProducts>

<specieReference specie="s031"/>

<specieReference specie="s032" stoichiometry="20"/>

<specieReference specie="s033" stoichiometry="20"/>

<specieReference specie="s034" stoichiometry="20"/>

<specieReference specie="s035" stoichiometry="20"/>

<specieReference specie="s036" stoichiometry="20"/>

<specieReference specie="s037" stoichiometry="20"/>

<specieReference specie="s038" stoichiometry="20"/>

<specieReference specie="s039" stoichiometry="20"/>

<specieReference specie="s040" stoichiometry="20"/>

<specieReference specie="s041" stoichiometry="20"/>

<specieReference specie="s042" stoichiometry="20"/>

<specieReference specie="s043" stoichiometry="20"/>

<specieReference specie="s044" stoichiometry="20"/>

<specieReference specie="s045" stoichiometry="20"/>

<specieReference specie="s046" stoichiometry="20"/>

<specieReference specie="s047" stoichiometry="20"/>

<specieReference specie="s048" stoichiometry="20"/>

<specieReference specie="s049" stoichiometry="20"/>

<specieReference specie="s050" stoichiometry="20"/>

<specieReference specie="s051" stoichiometry="20"/>

</listOfProducts>

<kineticLaw formula="c240*a_s031*(s031/vol)*s055/(s055+km240_s055)">

<listOfParameters>

<parameter name="c240" value="5e-025"/>

<parameter name="a_s031" value="1"/>

<parameter name="km240_s055" value="301"/>

<parameter name="vol" value="5e-016"/>

</listOfParameters>

</kineticLaw>

</reaction>

<reaction name="r241" reversible="false">

<listOfReactants>

<specieReference specie="s031"/>

<specieReference specie="s056"/>

</listOfReactants>

<listOfProducts>

<specieReference specie="s031"/>

<specieReference specie="s032" stoichiometry="20"/>

<specieReference specie="s033" stoichiometry="20"/>

<specieReference specie="s034" stoichiometry="20"/>

<specieReference specie="s035" stoichiometry="20"/>

<specieReference specie="s036" stoichiometry="20"/>

<specieReference specie="s037" stoichiometry="20"/>

<specieReference specie="s038" stoichiometry="20"/>

<specieReference specie="s039" stoichiometry="20"/>

<specieReference specie="s040" stoichiometry="20"/>

<specieReference specie="s041" stoichiometry="20"/>

<specieReference specie="s042" stoichiometry="20"/>

<specieReference specie="s043" stoichiometry="20"/>

<specieReference specie="s044" stoichiometry="20"/>

<specieReference specie="s045" stoichiometry="20"/>

<specieReference specie="s046" stoichiometry="20"/>

<specieReference specie="s047" stoichiometry="20"/>

<specieReference specie="s048" stoichiometry="20"/>

<specieReference specie="s049" stoichiometry="20"/>

<specieReference specie="s050" stoichiometry="20"/>

<specieReference specie="s051" stoichiometry="20"/>

</listOfProducts>

<kineticLaw formula="c241*a_s031*(s031/vol)*s056/(s056+km241_s056)">

<listOfParameters>

<parameter name="c241" value="5e-025"/>

<parameter name="a_s031" value="1"/>

<parameter name="km241_s056" value="301"/>

<parameter name="vol" value="5e-016"/>

</listOfParameters>

</kineticLaw>

</reaction>

<reaction name="r242" reversible="false">

<listOfReactants>

<specieReference specie="s031"/>

<specieReference specie="s057"/>

</listOfReactants>

<listOfProducts>

<specieReference specie="s031"/>

<specieReference specie="s032" stoichiometry="20"/>

<specieReference specie="s033" stoichiometry="20"/>

<specieReference specie="s034" stoichiometry="20"/>

<specieReference specie="s035" stoichiometry="20"/>

<specieReference specie="s036" stoichiometry="20"/>

<specieReference specie="s037" stoichiometry="20"/>

<specieReference specie="s038" stoichiometry="20"/>

<specieReference specie="s039" stoichiometry="20"/>

<specieReference specie="s040" stoichiometry="20"/>

<specieReference specie="s041" stoichiometry="20"/>

<specieReference specie="s042" stoichiometry="20"/>

<specieReference specie="s043" stoichiometry="20"/>

<specieReference specie="s044" stoichiometry="20"/>

<specieReference specie="s045" stoichiometry="20"/>

<specieReference specie="s046" stoichiometry="20"/>

<specieReference specie="s047" stoichiometry="20"/>

<specieReference specie="s048" stoichiometry="20"/>

<specieReference specie="s049" stoichiometry="20"/>

<specieReference specie="s050" stoichiometry="20"/>

<specieReference specie="s051" stoichiometry="20"/>

</listOfProducts>

<kineticLaw formula="c242*a_s031*(s031/vol)*s057/(s057+km242_s057)">

<listOfParameters>

<parameter name="c242" value="5e-025"/>

<parameter name="a_s031" value="1"/>

<parameter name="km242_s057" value="301"/>

<parameter name="vol" value="5e-016"/>

</listOfParameters>

</kineticLaw>

</reaction>

<reaction name="r243" reversible="false">

<listOfReactants>

<specieReference specie="s031"/>

<specieReference specie="s058"/>

</listOfReactants>

<listOfProducts>

<specieReference specie="s031"/>

<specieReference specie="s032" stoichiometry="20"/>

<specieReference specie="s033" stoichiometry="20"/>

<specieReference specie="s034" stoichiometry="20"/>

<specieReference specie="s035" stoichiometry="20"/>

<specieReference specie="s036" stoichiometry="20"/>

<specieReference specie="s037" stoichiometry="20"/>

<specieReference specie="s038" stoichiometry="20"/>

<specieReference specie="s039" stoichiometry="20"/>

<specieReference specie="s040" stoichiometry="20"/>

<specieReference specie="s041" stoichiometry="20"/>

<specieReference specie="s042" stoichiometry="20"/>

<specieReference specie="s043" stoichiometry="20"/>

<specieReference specie="s044" stoichiometry="20"/>

<specieReference specie="s045" stoichiometry="20"/>

<specieReference specie="s046" stoichiometry="20"/>

<specieReference specie="s047" stoichiometry="20"/>

<specieReference specie="s048" stoichiometry="20"/>

<specieReference specie="s049" stoichiometry="20"/>

<specieReference specie="s050" stoichiometry="20"/>

<specieReference specie="s051" stoichiometry="20"/>

</listOfProducts>

<kineticLaw formula="c243*a_s031*(s031/vol)*s058/(s058+km243_s058)">

<listOfParameters>

<parameter name="c243" value="5e-025"/>

<parameter name="a_s031" value="1"/>

<parameter name="km243_s058" value="301"/>

<parameter name="vol" value="5e-016"/>

</listOfParameters>

</kineticLaw>

</reaction>

<reaction name="r244" reversible="false">

<listOfReactants>

<specieReference specie="s031"/>

<specieReference specie="s059"/>

</listOfReactants>

<listOfProducts>

<specieReference specie="s031"/>

<specieReference specie="s032" stoichiometry="20"/>

<specieReference specie="s033" stoichiometry="20"/>

<specieReference specie="s034" stoichiometry="20"/>

<specieReference specie="s035" stoichiometry="20"/>

<specieReference specie="s036" stoichiometry="20"/>

<specieReference specie="s037" stoichiometry="20"/>

<specieReference specie="s038" stoichiometry="20"/>

<specieReference specie="s039" stoichiometry="20"/>

<specieReference specie="s040" stoichiometry="20"/>

<specieReference specie="s041" stoichiometry="20"/>

<specieReference specie="s042" stoichiometry="20"/>

<specieReference specie="s043" stoichiometry="20"/>

<specieReference specie="s044" stoichiometry="20"/>

<specieReference specie="s045" stoichiometry="20"/>

<specieReference specie="s046" stoichiometry="20"/>

<specieReference specie="s047" stoichiometry="20"/>

<specieReference specie="s048" stoichiometry="20"/>

<specieReference specie="s049" stoichiometry="20"/>

<specieReference specie="s050" stoichiometry="20"/>

<specieReference specie="s051" stoichiometry="20"/>

</listOfProducts>

<kineticLaw formula="c244*a_s031*(s031/vol)*s059/(s059+km244_s059)">

<listOfParameters>

<parameter name="c244" value="5e-025"/>

<parameter name="a_s031" value="1"/>

<parameter name="km244_s059" value="301"/>

<parameter name="vol" value="5e-016"/>

</listOfParameters>

</kineticLaw>

</reaction>

<reaction name="r245" reversible="false">

<listOfReactants>

<specieReference specie="s031"/>

<specieReference specie="s060"/>

</listOfReactants>

<listOfProducts>

<specieReference specie="s031"/>

<specieReference specie="s032" stoichiometry="20"/>

<specieReference specie="s033" stoichiometry="20"/>

<specieReference specie="s034" stoichiometry="20"/>

<specieReference specie="s035" stoichiometry="20"/>

<specieReference specie="s036" stoichiometry="20"/>

<specieReference specie="s037" stoichiometry="20"/>

<specieReference specie="s038" stoichiometry="20"/>

<specieReference specie="s039" stoichiometry="20"/>

<specieReference specie="s040" stoichiometry="20"/>

<specieReference specie="s041" stoichiometry="20"/>

<specieReference specie="s042" stoichiometry="20"/>

<specieReference specie="s043" stoichiometry="20"/>

<specieReference specie="s044" stoichiometry="20"/>

<specieReference specie="s045" stoichiometry="20"/>

<specieReference specie="s046" stoichiometry="20"/>

<specieReference specie="s047" stoichiometry="20"/>

<specieReference specie="s048" stoichiometry="20"/>

<specieReference specie="s049" stoichiometry="20"/>

<specieReference specie="s050" stoichiometry="20"/>

<specieReference specie="s051" stoichiometry="20"/>

</listOfProducts>

<kineticLaw formula="c245*a_s031*(s031/vol)*s060/(s060+km245_s060)">

<listOfParameters>

<parameter name="c245" value="5e-025"/>

<parameter name="a_s031" value="1"/>

<parameter name="km245_s060" value="301"/>

<parameter name="vol" value="5e-016"/>

</listOfParameters>

</kineticLaw>

</reaction>

<reaction name="r246" reversible="false">

<listOfReactants>

<specieReference specie="s031"/>

<specieReference specie="s061"/>

</listOfReactants>

<listOfProducts>

<specieReference specie="s031"/>

<specieReference specie="s032" stoichiometry="20"/>

<specieReference specie="s033" stoichiometry="20"/>

<specieReference specie="s034" stoichiometry="20"/>

<specieReference specie="s035" stoichiometry="20"/>

<specieReference specie="s036" stoichiometry="20"/>

<specieReference specie="s037" stoichiometry="20"/>

<specieReference specie="s038" stoichiometry="20"/>

<specieReference specie="s039" stoichiometry="20"/>

<specieReference specie="s040" stoichiometry="20"/>

<specieReference specie="s041" stoichiometry="20"/>

<specieReference specie="s042" stoichiometry="20"/>

<specieReference specie="s043" stoichiometry="20"/>

<specieReference specie="s044" stoichiometry="20"/>

<specieReference specie="s045" stoichiometry="20"/>

<specieReference specie="s046" stoichiometry="20"/>

<specieReference specie="s047" stoichiometry="20"/>

<specieReference specie="s048" stoichiometry="20"/>

<specieReference specie="s049" stoichiometry="20"/>

<specieReference specie="s050" stoichiometry="20"/>

<specieReference specie="s051" stoichiometry="20"/>

</listOfProducts>

<kineticLaw formula="c246*a_s031*(s031/vol)*s061/(s061+km246_s061)">

<listOfParameters>

<parameter name="c246" value="5e-025"/>

<parameter name="a_s031" value="1"/>

<parameter name="km246_s061" value="301"/>

<parameter name="vol" value="5e-016"/>

</listOfParameters>

</kineticLaw>

</reaction>

<reaction name="r247" reversible="false">

<listOfReactants>

<specieReference specie="s031"/>

<specieReference specie="s062"/>

</listOfReactants>

<listOfProducts>

<specieReference specie="s031"/>

<specieReference specie="s032" stoichiometry="20"/>

<specieReference specie="s033" stoichiometry="20"/>

<specieReference specie="s034" stoichiometry="20"/>

<specieReference specie="s035" stoichiometry="20"/>

<specieReference specie="s036" stoichiometry="20"/>

<specieReference specie="s037" stoichiometry="20"/>

<specieReference specie="s038" stoichiometry="20"/>

<specieReference specie="s039" stoichiometry="20"/>

<specieReference specie="s040" stoichiometry="20"/>

<specieReference specie="s041" stoichiometry="20"/>

<specieReference specie="s042" stoichiometry="20"/>

<specieReference specie="s043" stoichiometry="20"/>

<specieReference specie="s044" stoichiometry="20"/>

<specieReference specie="s045" stoichiometry="20"/>

<specieReference specie="s046" stoichiometry="20"/>

<specieReference specie="s047" stoichiometry="20"/>

<specieReference specie="s048" stoichiometry="20"/>

<specieReference specie="s049" stoichiometry="20"/>

<specieReference specie="s050" stoichiometry="20"/>

<specieReference specie="s051" stoichiometry="20"/>

</listOfProducts>

<kineticLaw formula="c247*a_s031*(s031/vol)*s062/(s062+km247_s062)">

<listOfParameters>

<parameter name="c247" value="5e-025"/>

<parameter name="a_s031" value="1"/>

<parameter name="km247_s062" value="301"/>

<parameter name="vol" value="5e-016"/>

</listOfParameters>

</kineticLaw>

</reaction>

<reaction name="r248" reversible="false">

<listOfReactants>

<specieReference specie="s031"/>

<specieReference specie="s063"/>

</listOfReactants>

<listOfProducts>

<specieReference specie="s031"/>

<specieReference specie="s032" stoichiometry="20"/>

<specieReference specie="s033" stoichiometry="20"/>

<specieReference specie="s034" stoichiometry="20"/>

<specieReference specie="s035" stoichiometry="20"/>

<specieReference specie="s036" stoichiometry="20"/>

<specieReference specie="s037" stoichiometry="20"/>

<specieReference specie="s038" stoichiometry="20"/>

<specieReference specie="s039" stoichiometry="20"/>

<specieReference specie="s040" stoichiometry="20"/>

<specieReference specie="s041" stoichiometry="20"/>

<specieReference specie="s042" stoichiometry="20"/>

<specieReference specie="s043" stoichiometry="20"/>

<specieReference specie="s044" stoichiometry="20"/>

<specieReference specie="s045" stoichiometry="20"/>

<specieReference specie="s046" stoichiometry="20"/>

<specieReference specie="s047" stoichiometry="20"/>

<specieReference specie="s048" stoichiometry="20"/>

<specieReference specie="s049" stoichiometry="20"/>

<specieReference specie="s050" stoichiometry="20"/>

<specieReference specie="s051" stoichiometry="20"/>

</listOfProducts>

<kineticLaw formula="c248*a_s031*(s031/vol)*s063/(s063+km248_s063)">

<listOfParameters>

<parameter name="c248" value="5e-025"/>

<parameter name="a_s031" value="1"/>

<parameter name="km248_s063" value="301"/>

<parameter name="vol" value="5e-016"/>

</listOfParameters>

</kineticLaw>

</reaction>

<reaction name="r249" reversible="false">

<listOfReactants>

<specieReference specie="s031"/>

<specieReference specie="s064"/>

</listOfReactants>

<listOfProducts>

<specieReference specie="s031"/>

<specieReference specie="s032" stoichiometry="20"/>

<specieReference specie="s033" stoichiometry="20"/>

<specieReference specie="s034" stoichiometry="20"/>

<specieReference specie="s035" stoichiometry="20"/>

<specieReference specie="s036" stoichiometry="20"/>

<specieReference specie="s037" stoichiometry="20"/>

<specieReference specie="s038" stoichiometry="20"/>

<specieReference specie="s039" stoichiometry="20"/>

<specieReference specie="s040" stoichiometry="20"/>

<specieReference specie="s041" stoichiometry="20"/>

<specieReference specie="s042" stoichiometry="20"/>

<specieReference specie="s043" stoichiometry="20"/>

<specieReference specie="s044" stoichiometry="20"/>

<specieReference specie="s045" stoichiometry="20"/>

<specieReference specie="s046" stoichiometry="20"/>

<specieReference specie="s047" stoichiometry="20"/>

<specieReference specie="s048" stoichiometry="20"/>

<specieReference specie="s049" stoichiometry="20"/>

<specieReference specie="s050" stoichiometry="20"/>

<specieReference specie="s051" stoichiometry="20"/>

</listOfProducts>

<kineticLaw formula="c249*a_s031*(s031/vol)*s064/(s064+km249_s064)">

<listOfParameters>

<parameter name="c249" value="5e-025"/>

<parameter name="a_s031" value="1"/>

<parameter name="km249_s064" value="301"/>

<parameter name="vol" value="5e-016"/>

</listOfParameters>

</kineticLaw>

</reaction>

<reaction name="r250" reversible="false">

<listOfReactants>

<specieReference specie="s031"/>

<specieReference specie="s065"/>

</listOfReactants>

<listOfProducts>

<specieReference specie="s031"/>

<specieReference specie="s032" stoichiometry="20"/>

<specieReference specie="s033" stoichiometry="20"/>

<specieReference specie="s034" stoichiometry="20"/>

<specieReference specie="s035" stoichiometry="20"/>

<specieReference specie="s036" stoichiometry="20"/>

<specieReference specie="s037" stoichiometry="20"/>

<specieReference specie="s038" stoichiometry="20"/>

<specieReference specie="s039" stoichiometry="20"/>

<specieReference specie="s040" stoichiometry="20"/>

<specieReference specie="s041" stoichiometry="20"/>

<specieReference specie="s042" stoichiometry="20"/>

<specieReference specie="s043" stoichiometry="20"/>

<specieReference specie="s044" stoichiometry="20"/>

<specieReference specie="s045" stoichiometry="20"/>

<specieReference specie="s046" stoichiometry="20"/>

<specieReference specie="s047" stoichiometry="20"/>

<specieReference specie="s048" stoichiometry="20"/>

<specieReference specie="s049" stoichiometry="20"/>

<specieReference specie="s050" stoichiometry="20"/>

<specieReference specie="s051" stoichiometry="20"/>

</listOfProducts>

<kineticLaw formula="c250*a_s031*(s031/vol)*s065/(s065+km250_s065)">

<listOfParameters>

<parameter name="c250" value="5e-025"/>

<parameter name="a_s031" value="1"/>

<parameter name="km250_s065" value="301"/>

<parameter name="vol" value="5e-016"/>

</listOfParameters>

</kineticLaw>

</reaction>

<reaction name="r251" reversible="false">

<listOfReactants>

<specieReference specie="s031"/>

<specieReference specie="s066"/>

</listOfReactants>

<listOfProducts>

<specieReference specie="s031"/>

<specieReference specie="s032" stoichiometry="20"/>

<specieReference specie="s033" stoichiometry="20"/>

<specieReference specie="s034" stoichiometry="20"/>

<specieReference specie="s035" stoichiometry="20"/>

<specieReference specie="s036" stoichiometry="20"/>

<specieReference specie="s037" stoichiometry="20"/>

<specieReference specie="s038" stoichiometry="20"/>

<specieReference specie="s039" stoichiometry="20"/>

<specieReference specie="s040" stoichiometry="20"/>

<specieReference specie="s041" stoichiometry="20"/>

<specieReference specie="s042" stoichiometry="20"/>

<specieReference specie="s043" stoichiometry="20"/>

<specieReference specie="s044" stoichiometry="20"/>

<specieReference specie="s045" stoichiometry="20"/>

<specieReference specie="s046" stoichiometry="20"/>

<specieReference specie="s047" stoichiometry="20"/>

<specieReference specie="s048" stoichiometry="20"/>

<specieReference specie="s049" stoichiometry="20"/>

<specieReference specie="s050" stoichiometry="20"/>

<specieReference specie="s051" stoichiometry="20"/>

</listOfProducts>

<kineticLaw formula="c251*a_s031*(s031/vol)*s066/(s066+km251_s066)">

<listOfParameters>

<parameter name="c251" value="5e-025"/>

<parameter name="a_s031" value="1"/>

<parameter name="km251_s066" value="301"/>

<parameter name="vol" value="5e-016"/>

</listOfParameters>

</kineticLaw>

</reaction>

<reaction name="r252" reversible="false">

<listOfReactants>

<specieReference specie="s031"/>

<specieReference specie="s067"/>

</listOfReactants>

<listOfProducts>

<specieReference specie="s031"/>

<specieReference specie="s032" stoichiometry="20"/>

<specieReference specie="s033" stoichiometry="20"/>

<specieReference specie="s034" stoichiometry="20"/>

<specieReference specie="s035" stoichiometry="20"/>

<specieReference specie="s036" stoichiometry="20"/>

<specieReference specie="s037" stoichiometry="20"/>

<specieReference specie="s038" stoichiometry="20"/>

<specieReference specie="s039" stoichiometry="20"/>

<specieReference specie="s040" stoichiometry="20"/>

<specieReference specie="s041" stoichiometry="20"/>

<specieReference specie="s042" stoichiometry="20"/>

<specieReference specie="s043" stoichiometry="20"/>

<specieReference specie="s044" stoichiometry="20"/>

<specieReference specie="s045" stoichiometry="20"/>

<specieReference specie="s046" stoichiometry="20"/>

<specieReference specie="s047" stoichiometry="20"/>

<specieReference specie="s048" stoichiometry="20"/>

<specieReference specie="s049" stoichiometry="20"/>

<specieReference specie="s050" stoichiometry="20"/>

<specieReference specie="s051" stoichiometry="20"/>

</listOfProducts>

<kineticLaw formula="c252*a_s031*(s031/vol)*s067/(s067+km252_s067)">

<listOfParameters>

<parameter name="c252" value="5e-025"/>

<parameter name="a_s031" value="1"/>

<parameter name="km252_s067" value="301"/>

<parameter name="vol" value="5e-016"/>

</listOfParameters>

</kineticLaw>

</reaction>

<reaction name="r253" reversible="false">

<listOfReactants>

<specieReference specie="s031"/>

<specieReference specie="s068"/>

</listOfReactants>

<listOfProducts>

<specieReference specie="s031"/>

<specieReference specie="s032" stoichiometry="20"/>

<specieReference specie="s033" stoichiometry="20"/>

<specieReference specie="s034" stoichiometry="20"/>

<specieReference specie="s035" stoichiometry="20"/>

<specieReference specie="s036" stoichiometry="20"/>

<specieReference specie="s037" stoichiometry="20"/>

<specieReference specie="s038" stoichiometry="20"/>

<specieReference specie="s039" stoichiometry="20"/>

<specieReference specie="s040" stoichiometry="20"/>

<specieReference specie="s041" stoichiometry="20"/>

<specieReference specie="s042" stoichiometry="20"/>

<specieReference specie="s043" stoichiometry="20"/>

<specieReference specie="s044" stoichiometry="20"/>

<specieReference specie="s045" stoichiometry="20"/>

<specieReference specie="s046" stoichiometry="20"/>

<specieReference specie="s047" stoichiometry="20"/>

<specieReference specie="s048" stoichiometry="20"/>

<specieReference specie="s049" stoichiometry="20"/>

<specieReference specie="s050" stoichiometry="20"/>

<specieReference specie="s051" stoichiometry="20"/>

</listOfProducts>

<kineticLaw formula="c253*a_s031*(s031/vol)*s068/(s068+km253_s068)">

<listOfParameters>

<parameter name="c253" value="5e-025"/>

<parameter name="a_s031" value="1"/>

<parameter name="km253_s068" value="301"/>

<parameter name="vol" value="5e-016"/>

</listOfParameters>

</kineticLaw>

</reaction>

<reaction name="r254" reversible="false">

<listOfReactants>

<specieReference specie="s031"/>

<specieReference specie="s069"/>

</listOfReactants>

<listOfProducts>

<specieReference specie="s031"/>

<specieReference specie="s032" stoichiometry="20"/>

<specieReference specie="s033" stoichiometry="20"/>

<specieReference specie="s034" stoichiometry="20"/>

<specieReference specie="s035" stoichiometry="20"/>

<specieReference specie="s036" stoichiometry="20"/>

<specieReference specie="s037" stoichiometry="20"/>

<specieReference specie="s038" stoichiometry="20"/>

<specieReference specie="s039" stoichiometry="20"/>

<specieReference specie="s040" stoichiometry="20"/>

<specieReference specie="s041" stoichiometry="20"/>

<specieReference specie="s042" stoichiometry="20"/>

<specieReference specie="s043" stoichiometry="20"/>

<specieReference specie="s044" stoichiometry="20"/>

<specieReference specie="s045" stoichiometry="20"/>

<specieReference specie="s046" stoichiometry="20"/>

<specieReference specie="s047" stoichiometry="20"/>

<specieReference specie="s048" stoichiometry="20"/>

<specieReference specie="s049" stoichiometry="20"/>

<specieReference specie="s050" stoichiometry="20"/>

<specieReference specie="s051" stoichiometry="20"/>

</listOfProducts>

<kineticLaw formula="c254*a_s031*(s031/vol)*s069/(s069+km254_s069)">

<listOfParameters>

<parameter name="c254" value="5e-025"/>

<parameter name="a_s031" value="1"/>

<parameter name="km254_s069" value="301"/>

<parameter name="vol" value="5e-016"/>

</listOfParameters>

</kineticLaw>

</reaction>

<reaction name="r255" reversible="false">

<listOfReactants>

<specieReference specie="s031"/>

<specieReference specie="s070"/>

</listOfReactants>

<listOfProducts>

<specieReference specie="s031"/>

<specieReference specie="s032" stoichiometry="20"/>

<specieReference specie="s033" stoichiometry="20"/>

<specieReference specie="s034" stoichiometry="20"/>

<specieReference specie="s035" stoichiometry="20"/>

<specieReference specie="s036" stoichiometry="20"/>

<specieReference specie="s037" stoichiometry="20"/>

<specieReference specie="s038" stoichiometry="20"/>

<specieReference specie="s039" stoichiometry="20"/>

<specieReference specie="s040" stoichiometry="20"/>

<specieReference specie="s041" stoichiometry="20"/>

<specieReference specie="s042" stoichiometry="20"/>

<specieReference specie="s043" stoichiometry="20"/>

<specieReference specie="s044" stoichiometry="20"/>

<specieReference specie="s045" stoichiometry="20"/>

<specieReference specie="s046" stoichiometry="20"/>

<specieReference specie="s047" stoichiometry="20"/>

<specieReference specie="s048" stoichiometry="20"/>

<specieReference specie="s049" stoichiometry="20"/>

<specieReference specie="s050" stoichiometry="20"/>

<specieReference specie="s051" stoichiometry="20"/>

</listOfProducts>

<kineticLaw formula="c255*a_s031*(s031/vol)*s070/(s070+km255_s070)">

<listOfParameters>

<parameter name="c255" value="5e-025"/>

<parameter name="a_s031" value="1"/>

<parameter name="km255_s070" value="301"/>

<parameter name="vol" value="5e-016"/>

</listOfParameters>

</kineticLaw>

</reaction>

<reaction name="r256" reversible="false">

<listOfReactants>

<specieReference specie="s031"/>

<specieReference specie="s071"/>

</listOfReactants>

<listOfProducts>

<specieReference specie="s031"/>

<specieReference specie="s032" stoichiometry="20"/>

<specieReference specie="s033" stoichiometry="20"/>

<specieReference specie="s034" stoichiometry="20"/>

<specieReference specie="s035" stoichiometry="20"/>

<specieReference specie="s036" stoichiometry="20"/>

<specieReference specie="s037" stoichiometry="20"/>

<specieReference specie="s038" stoichiometry="20"/>

<specieReference specie="s039" stoichiometry="20"/>

<specieReference specie="s040" stoichiometry="20"/>

<specieReference specie="s041" stoichiometry="20"/>

<specieReference specie="s042" stoichiometry="20"/>

<specieReference specie="s043" stoichiometry="20"/>

<specieReference specie="s044" stoichiometry="20"/>

<specieReference specie="s045" stoichiometry="20"/>

<specieReference specie="s046" stoichiometry="20"/>

<specieReference specie="s047" stoichiometry="20"/>

<specieReference specie="s048" stoichiometry="20"/>

<specieReference specie="s049" stoichiometry="20"/>

<specieReference specie="s050" stoichiometry="20"/>

<specieReference specie="s051" stoichiometry="20"/>

</listOfProducts>

<kineticLaw formula="c256*a_s031*(s031/vol)*s071/(s071+km256_s071)">

<listOfParameters>

<parameter name="c256" value="5e-025"/>

<parameter name="a_s031" value="1"/>

<parameter name="km256_s071" value="301"/>

<parameter name="vol" value="5e-016"/>

</listOfParameters>

</kineticLaw>

</reaction>

<reaction name="r257" reversible="false">

<listOfReactants>

<specieReference specie="s031"/>

<specieReference specie="s030"/>

</listOfReactants>

<listOfProducts>

<specieReference specie="s031"/>

<specieReference specie="s032" stoichiometry="20"/>

<specieReference specie="s033" stoichiometry="20"/>

<specieReference specie="s034" stoichiometry="20"/>

<specieReference specie="s035" stoichiometry="20"/>

<specieReference specie="s036" stoichiometry="20"/>

<specieReference specie="s037" stoichiometry="20"/>

<specieReference specie="s038" stoichiometry="20"/>

<specieReference specie="s039" stoichiometry="20"/>

<specieReference specie="s040" stoichiometry="20"/>

<specieReference specie="s041" stoichiometry="20"/>

<specieReference specie="s042" stoichiometry="20"/>

<specieReference specie="s043" stoichiometry="20"/>

<specieReference specie="s044" stoichiometry="20"/>

<specieReference specie="s045" stoichiometry="20"/>

<specieReference specie="s046" stoichiometry="20"/>

<specieReference specie="s047" stoichiometry="20"/>

<specieReference specie="s048" stoichiometry="20"/>

<specieReference specie="s049" stoichiometry="20"/>

<specieReference specie="s050" stoichiometry="20"/>

<specieReference specie="s051" stoichiometry="20"/>

</listOfProducts>

<kineticLaw formula="c257*a_s031*(s031/vol)*s030/(s030+km257_s030)">

<listOfParameters>

<parameter name="c257" value="5e-025"/>

<parameter name="a_s031" value="1"/>

<parameter name="km257_s030" value="301"/>

<parameter name="vol" value="5e-016"/>

</listOfParameters>

</kineticLaw>

</reaction>

<reaction name="r258" reversible="false">

<listOfReactants>

<specieReference specie="s031"/>

<specieReference specie="s001"/>

</listOfReactants>

<listOfProducts>

<specieReference specie="s031"/>

<specieReference specie="s032" stoichiometry="20"/>

<specieReference specie="s033" stoichiometry="20"/>

<specieReference specie="s034" stoichiometry="20"/>

<specieReference specie="s035" stoichiometry="20"/>

<specieReference specie="s036" stoichiometry="20"/>

<specieReference specie="s037" stoichiometry="20"/>

<specieReference specie="s038" stoichiometry="20"/>

<specieReference specie="s039" stoichiometry="20"/>

<specieReference specie="s040" stoichiometry="20"/>

<specieReference specie="s041" stoichiometry="20"/>

<specieReference specie="s042" stoichiometry="20"/>

<specieReference specie="s043" stoichiometry="20"/>

<specieReference specie="s044" stoichiometry="20"/>

<specieReference specie="s045" stoichiometry="20"/>

<specieReference specie="s046" stoichiometry="20"/>

<specieReference specie="s047" stoichiometry="20"/>

<specieReference specie="s048" stoichiometry="20"/>

<specieReference specie="s049" stoichiometry="20"/>

<specieReference specie="s050" stoichiometry="20"/>

<specieReference specie="s051" stoichiometry="20"/>

</listOfProducts>

<kineticLaw formula="c258*a_s031*(s031/vol)*s001/(s001+km258_s001)">

<listOfParameters>

<parameter name="c258" value="5e-025"/>

<parameter name="a_s031" value="1"/>

<parameter name="km258_s001" value="301"/>

<parameter name="vol" value="5e-016"/>

</listOfParameters>

</kineticLaw>

</reaction>

<reaction name="r259" reversible="false">

<listOfReactants>

<specieReference specie="s031"/>

<specieReference specie="s031"/>

</listOfReactants>

<listOfProducts>

<specieReference specie="s031"/>

<specieReference specie="s032" stoichiometry="20"/>

<specieReference specie="s033" stoichiometry="20"/>

<specieReference specie="s034" stoichiometry="20"/>

<specieReference specie="s035" stoichiometry="20"/>

<specieReference specie="s036" stoichiometry="20"/>

<specieReference specie="s037" stoichiometry="20"/>

<specieReference specie="s038" stoichiometry="20"/>

<specieReference specie="s039" stoichiometry="20"/>

<specieReference specie="s040" stoichiometry="20"/>

<specieReference specie="s041" stoichiometry="20"/>

<specieReference specie="s042" stoichiometry="20"/>

<specieReference specie="s043" stoichiometry="20"/>

<specieReference specie="s044" stoichiometry="20"/>

<specieReference specie="s045" stoichiometry="20"/>

<specieReference specie="s046" stoichiometry="20"/>

<specieReference specie="s047" stoichiometry="20"/>

<specieReference specie="s048" stoichiometry="20"/>

<specieReference specie="s049" stoichiometry="20"/>

<specieReference specie="s050" stoichiometry="20"/>

<specieReference specie="s051" stoichiometry="20"/>

</listOfProducts>

<kineticLaw formula="c259*a_s031*(s031/vol)*s031/(s031+km259_s031)">

<listOfParameters>

<parameter name="c259" value="5e-025"/>

<parameter name="a_s031" value="1"/>

<parameter name="km259_s031" value="301"/>

<parameter name="vol" value="5e-016"/>

</listOfParameters>

</kineticLaw>

</reaction>

<reaction name="r260" reversible="false">

<listOfReactants>

<specieReference specie="s031"/>

<specieReference specie="s018"/>

</listOfReactants>

<listOfProducts>

<specieReference specie="s031"/>

<specieReference specie="s047" stoichiometry="44"/>

<specieReference specie="s044" stoichiometry="9"/>

<specieReference specie="s035" stoichiometry="27"/>

<specieReference specie="s048" stoichiometry="43"/>

<specieReference specie="s037" stoichiometry="22"/>

<specieReference specie="s039" stoichiometry="40"/>

<specieReference specie="s045" stoichiometry="7"/>

<specieReference specie="s032" stoichiometry="31"/>

<specieReference specie="s043" stoichiometry="23"/>

<specieReference specie="s040" stoichiometry="53"/>

<specieReference specie="s033" stoichiometry="13"/>

<specieReference specie="s042" stoichiometry="18"/>

<specieReference specie="s036" stoichiometry="25"/>

<specieReference specie="s046" stoichiometry="20"/>

<specieReference specie="s038" stoichiometry="34"/>

<specieReference specie="s049" stoichiometry="29"/>

<specieReference specie="s034" stoichiometry="29"/>

<specieReference specie="s041" stoichiometry="8"/>

<specieReference specie="s051" stoichiometry="21"/>

<specieReference specie="s050" stoichiometry="21"/>

</listOfProducts>

<kineticLaw formula="c260*a_s031*(s031/vol)*s018/(s018+km260_s018)">

<listOfParameters>

<parameter name="c260" value="5e-025"/>

<parameter name="a_s031" value="1"/>

<parameter name="km260_s018" value="301"/>

<parameter name="vol" value="5e-016"/>

</listOfParameters>

</kineticLaw>

</reaction>

<reaction name="r261" reversible="false">

<listOfReactants>

<specieReference specie="s031"/>

<specieReference specie="s027"/>

</listOfReactants>

<listOfProducts>

<specieReference specie="s031"/>

<specieReference specie="s047" stoichiometry="44"/>

<specieReference specie="s044" stoichiometry="9"/>

<specieReference specie="s035" stoichiometry="27"/>

<specieReference specie="s048" stoichiometry="43"/>

<specieReference specie="s037" stoichiometry="22"/>

<specieReference specie="s039" stoichiometry="40"/>

<specieReference specie="s045" stoichiometry="7"/>

<specieReference specie="s032" stoichiometry="31"/>

<specieReference specie="s043" stoichiometry="23"/>

<specieReference specie="s040" stoichiometry="53"/>

<specieReference specie="s033" stoichiometry="13"/>

<specieReference specie="s042" stoichiometry="18"/>

<specieReference specie="s036" stoichiometry="25"/>

<specieReference specie="s046" stoichiometry="20"/>

<specieReference specie="s038" stoichiometry="34"/>

<specieReference specie="s049" stoichiometry="29"/>

<specieReference specie="s034" stoichiometry="29"/>

<specieReference specie="s041" stoichiometry="8"/>

<specieReference specie="s051" stoichiometry="21"/>

<specieReference specie="s050" stoichiometry="21"/>

</listOfProducts>

<kineticLaw formula="c261*a_s031*(s031/vol)*s027/(s027+km261_s027)">

<listOfParameters>

<parameter name="c261" value="5e-025"/>

<parameter name="a_s031" value="1"/>

<parameter name="km261_s027" value="301"/>

<parameter name="vol" value="5e-016"/>

</listOfParameters>

</kineticLaw>

</reaction>

<reaction name="r262" reversible="false">

<listOfReactants>

<specieReference specie="s027" stoichiometry="2"/>

</listOfReactants>

<listOfProducts>

<specieReference specie="s028"/>

</listOfProducts>

<kineticLaw formula="c262*s027*(s027-1)/(2*vol)">

<listOfParameters>

<parameter name="c262" value="5e-017"/>

<parameter name="vol" value="5e-016"/>

</listOfParameters>

</kineticLaw>

</reaction>

<reaction name="r263" reversible="false">

<listOfReactants>

<specieReference specie="s028"/>

</listOfReactants>

<listOfProducts>

<specieReference specie="s027" stoichiometry="2"/>

</listOfProducts>

<kineticLaw formula="c263*s028">

<listOfParameters>

<parameter name="c263" value="0.1"/>

</listOfParameters>

</kineticLaw>

</reaction>

<reaction name="r264" reversible="false">

<listOfReactants>

<specieReference specie="s028" stoichiometry="2"/>

</listOfReactants>

<listOfProducts>

<specieReference specie="s029"/>

</listOfProducts>

<kineticLaw formula="c264*s028*(s028-1)/(2*vol)">

<listOfParameters>

<parameter name="c264" value="5e-017"/>

<parameter name="vol" value="5e-016"/>

</listOfParameters>

</kineticLaw>

</reaction>

<reaction name="r265" reversible="false">

<listOfReactants>

<specieReference specie="s029"/>

</listOfReactants>

<listOfProducts>

<specieReference specie="s028" stoichiometry="2"/>

</listOfProducts>

<kineticLaw formula="c265*s029">

<listOfParameters>

<parameter name="c265" value="0.1"/>

</listOfParameters>

</kineticLaw>

</reaction>

<reaction name="r266" reversible="false">

<listOfReactants>

<specieReference specie="s018"/>

<specieReference specie="s005"/>

</listOfReactants>

<listOfProducts>

<specieReference specie="s232"/>

</listOfProducts>

<kineticLaw formula="c266*s018*s005/vol">

<listOfParameters>

<parameter name="c266" value="1e-016"/>

<parameter name="vol" value="5e-016"/>

</listOfParameters>

</kineticLaw>

</reaction>

<reaction name="r267" reversible="false">

<listOfReactants>

<specieReference specie="s232"/>

</listOfReactants>

<listOfProducts>

<specieReference specie="s018"/>

<specieReference specie="s005"/>

</listOfProducts>

<kineticLaw formula="c267*s232">

<listOfParameters>

<parameter name="c267" value="0.1"/>

</listOfParameters>

</kineticLaw>

</reaction>

<reaction name="r268" reversible="false">

<listOfReactants>

<specieReference specie="s232"/>

<specieReference specie="s233"/>

</listOfReactants>

<listOfProducts>

<specieReference specie="s234"/>

</listOfProducts>

<kineticLaw formula="c268*s232*s233/vol">

<listOfParameters>

<parameter name="c268" value="1e-016"/>

<parameter name="vol" value="5e-016"/>

</listOfParameters>

</kineticLaw>

</reaction>

<reaction name="r269" reversible="false">

<listOfReactants>

<specieReference specie="s234"/>

</listOfReactants>

<listOfProducts>

<specieReference specie="s232"/>

<specieReference specie="s233"/>

</listOfProducts>

<kineticLaw formula="c269*s234">

<listOfParameters>

<parameter name="c269" value="0.1"/>

</listOfParameters>

</kineticLaw>

</reaction>

<reaction name="r270" reversible="false">

<listOfReactants>

<specieReference specie="s234"/>

</listOfReactants>

<listOfProducts>

<specieReference specie="s235"/>

</listOfProducts>

<kineticLaw formula="c270*s234">

<listOfParameters>

<parameter name="c270" value="0.5"/>

</listOfParameters>

</kineticLaw>

</reaction>

<reaction name="r271" reversible="false">

<listOfReactants>

<specieReference specie="s235"/>

</listOfReactants>

<listOfProducts>

<specieReference specie="s236"/>

<specieReference specie="s021"/>

</listOfProducts>

<kineticLaw formula="c271*s235">

<listOfParameters>

<parameter name="c271" value="3"/>

</listOfParameters>

</kineticLaw>

</reaction>

<reaction name="r272" reversible="false">

<listOfReactants>

<specieReference specie="s236"/>

<specieReference specie="s237"/>

</listOfReactants>

<listOfProducts>

<specieReference specie="s238"/>

</listOfProducts>

<kineticLaw formula="c272*s236*s237/vol">

<listOfParameters>

<parameter name="c272" value="1e-016"/>

<parameter name="vol" value="5e-016"/>

</listOfParameters>

</kineticLaw>

</reaction>

<reaction name="r273" reversible="false">

<listOfReactants>

<specieReference specie="s238"/>

</listOfReactants>

<listOfProducts>

<specieReference specie="s236"/>

<specieReference specie="s237"/>

</listOfProducts>

<kineticLaw formula="c273*s238">

<listOfParameters>

<parameter name="c273" value="0.1"/>

</listOfParameters>

</kineticLaw>

</reaction>

<reaction name="r274" reversible="false">

<listOfReactants>

<specieReference specie="s238"/>

</listOfReactants>

<listOfProducts>

<specieReference specie="s239"/>

<specieReference specie="s020"/>

</listOfProducts>

<kineticLaw formula="c274*s238">

<listOfParameters>

<parameter name="c274" value="3"/>

</listOfParameters>

</kineticLaw>

</reaction>

<reaction name="r275" reversible="false">

<listOfReactants>

<specieReference specie="s239"/>

</listOfReactants>

<listOfProducts>

<specieReference specie="s240"/>

<specieReference specie="s018"/>

</listOfProducts>

<kineticLaw formula="c275*s239">

<listOfParameters>

<parameter name="c275" value="3"/>

</listOfParameters>

</kineticLaw>

</reaction>

<reaction name="r276" reversible="false">

<listOfReactants>

<specieReference specie="s029"/>

<specieReference specie="s005"/>

</listOfReactants>

<listOfProducts>

<specieReference specie="s241"/>

</listOfProducts>

<kineticLaw formula="c276*s029*s005/vol">

<listOfParameters>

<parameter name="c276" value="5e-016"/>

<parameter name="vol" value="5e-016"/>

</listOfParameters>

</kineticLaw>

</reaction>

<reaction name="r277" reversible="false">

<listOfReactants>

<specieReference specie="s241"/>

</listOfReactants>

<listOfProducts>

<specieReference specie="s029"/>

<specieReference specie="s005"/>

</listOfProducts>

<kineticLaw formula="c277*s241">

<listOfParameters>

<parameter name="c277" value="0.1"/>

</listOfParameters>

</kineticLaw>

</reaction>

<reaction name="r278" reversible="false">

<listOfReactants>

<specieReference specie="s240"/>

<specieReference specie="s241"/>

</listOfReactants>

<listOfProducts>

<specieReference specie="s242"/>

</listOfProducts>

<kineticLaw formula="c278*s240*s241/vol">

<listOfParameters>

<parameter name="c278" value="1e-016"/>

<parameter name="vol" value="5e-016"/>

</listOfParameters>

</kineticLaw>

</reaction>

<reaction name="r279" reversible="false">

<listOfReactants>

<specieReference specie="s242"/>

</listOfReactants>

<listOfProducts>

<specieReference specie="s240"/>

<specieReference specie="s241"/>

</listOfProducts>

<kineticLaw formula="c279*s242">

<listOfParameters>

<parameter name="c279" value="0.1"/>

</listOfParameters>

</kineticLaw>

</reaction>

<reaction name="r280" reversible="false">

<listOfReactants>

<specieReference specie="s242"/>

</listOfReactants>

<listOfProducts>

<specieReference specie="s243"/>

</listOfProducts>

<kineticLaw formula="c280*s242">

<listOfParameters>

<parameter name="c280" value="5"/>

</listOfParameters>

</kineticLaw>

</reaction>

<reaction name="r281" reversible="false">

<listOfReactants>

<specieReference specie="s243"/>

</listOfReactants>

<listOfProducts>

<specieReference specie="s244"/>

<specieReference specie="s021"/>

</listOfProducts>

<kineticLaw formula="c281*s243">

<listOfParameters>

<parameter name="c281" value="4"/>

</listOfParameters>

</kineticLaw>

</reaction>

<reaction name="r282" reversible="false">

<listOfReactants>

<specieReference specie="s244"/>

<specieReference specie="s245"/>

</listOfReactants>

<listOfProducts>

<specieReference specie="s246"/>

</listOfProducts>

<kineticLaw formula="c282*s244*s245/vol">

<listOfParameters>

<parameter name="c282" value="5e-016"/>

<parameter name="vol" value="5e-016"/>

</listOfParameters>

</kineticLaw>

</reaction>

<reaction name="r283" reversible="false">

<listOfReactants>

<specieReference specie="s246"/>

</listOfReactants>

<listOfProducts>

<specieReference specie="s244"/>

<specieReference specie="s245"/>

</listOfProducts>

<kineticLaw formula="c283*s246">

<listOfParameters>

<parameter name="c283" value="0.1"/>

</listOfParameters>

</kineticLaw>

</reaction>

<reaction name="r284" reversible="false">

<listOfReactants>

<specieReference specie="s246"/>

</listOfReactants>

<listOfProducts>

<specieReference specie="s247"/>

<specieReference specie="s020"/>

</listOfProducts>

<kineticLaw formula="c284*s246">

<listOfParameters>

<parameter name="c284" value="4"/>

</listOfParameters>

</kineticLaw>

</reaction>

<reaction name="r285" reversible="false">

<listOfReactants>

<specieReference specie="s247"/>

</listOfReactants>

<listOfProducts>

<specieReference specie="s248"/>

<specieReference specie="s029"/>

</listOfProducts>

<kineticLaw formula="c285*s247">

<listOfParameters>

<parameter name="c285" value="4"/>

</listOfParameters>

</kineticLaw>

</reaction>

<reaction name="r286" reversible="false">

<listOfReactants>

<specieReference specie="s248"/>

<specieReference specie="s018"/>

</listOfReactants>

<listOfProducts>

<specieReference specie="s249"/>

</listOfProducts>

<kineticLaw formula="c286*s248*s018/vol">

<listOfParameters>

<parameter name="c286" value="1e-018"/>

<parameter name="vol" value="5e-016"/>

</listOfParameters>

</kineticLaw>

</reaction>

<reaction name="r287" reversible="false">

<listOfReactants>

<specieReference specie="s249"/>

</listOfReactants>

<listOfProducts>

<specieReference specie="s248"/>

<specieReference specie="s018"/>

</listOfProducts>

<kineticLaw formula="c287*s249">

<listOfParameters>

<parameter name="c287" value="0.1"/>

</listOfParameters>

</kineticLaw>

</reaction>

</listOfReactions>

</model>

</sbml>

**Model Parameters**

**Table S1**: Composition of *geneA*, *geneB*, *Pro_A*, *Pro_B*, *RNAp*, *RNase*, *Prot*, *AA_i_Trans* and *AA_i_tRNA* molecules in the geneA_geneB_CFTT_1p1 model.

| **Stoichiometry of Molecules** | | |
| --- | --- | --- |
| Nucleotide composition of the *geneA* and *geneB* | ATP | 381 |
|  | GTP | 429 |
|  | CTP | 369 |
|  | UTP | 377 |
| Energy requirements for *Pro_A* and *Pro_B* formation | ATP | 517 |
|  | GTP | 1552 |
| Amino acid composition of *Pro_A* and *Pro_B* protein | isoleucine | 31 |
|  | methionine | 13 |
|  | threonine | 29 |
|  | aspartic acid | 27 |
|  | proline | 25 |
|  | phenylalanine | 22 |
|  | arginine | 34 |
|  | glycine | 40 |
|  | leucine | 53 |
|  | tryptophan | 8 |
|  | asparagine | 18 |
|  | lysine | 23 |
|  | cysteine | 9 |
|  | histidine | 7 |
|  | glutamine | 20 |
|  | alanine | 44 |
|  | glutamic acid | 43 |
|  | serine | 29 |
|  | tyrosine | 21 |
|  | valine | 21 |
| Amino acid composition of *RNAp* | isoleucine | 20 |
|  | methionine | 20 |
|  | threonine | 20 |
|  | aspartic acid | 20 |
|  | proline | 20 |
|  | phenylalanine | 20 |
|  | arginine | 20 |
|  | glycine | 20 |
|  | leucine | 20 |
|  | tryptophan | 20 |
|  | asparagine | 20 |
|  | lysine | 20 |
|  | cysteine | 20 |
|  | histidine | 20 |
|  | glutamine | 20 |
|  | alanine | 20 |
|  | glutamic acid | 20 |
|  | serine | 20 |
|  | tyrosine | 20 |
|  | valine | 20 |
| Amino acid composition of the generic *RNase* | isoleucine | 20 |
|  | methionine | 20 |
|  | threonine | 20 |
|  | aspartic acid | 20 |
|  | proline | 20 |
|  | phenylalanine | 20 |
|  | arginine | 20 |
|  | glycine | 20 |
|  | leucine | 20 |
|  | tryptophan | 20 |
|  | asparagine | 20 |
|  | lysine | 20 |
|  | cysteine | 20 |
|  | histidine | 20 |
|  | glutamine | 20 |
|  | alanine | 20 |
|  | glutamic acid | 20 |
|  | serine | 20 |
|  | tyrosine | 20 |
|  | valine | 20 |
| Amino acid composition of the generic *Prot* | isoleucine | 20 |
|  | methionine | 20 |
|  | threonine | 20 |
|  | aspartic acid | 20 |
|  | proline | 20 |
|  | phenylalanine | 20 |
|  | arginine | 20 |
|  | glycine | 20 |
|  | leucine | 20 |
|  | tryptophan | 20 |
|  | asparagine | 20 |
|  | lysine | 20 |
|  | cysteine | 20 |
|  | histidine | 20 |
|  | glutamine | 20 |
|  | alanine | 20 |
|  | glutamic acid | 20 |
|  | serine | 20 |
|  | tyrosine | 20 |
|  | valine | 20 |

| Amino acid composition of the 20 *AA_i_Trans* | isoleucine | 20 |
| --- | --- | --- |
|  | methionine | 20 |
|  | threonine | 20 |
|  | aspartic acid | 20 |
|  | proline | 20 |
|  | phenylalanine | 20 |
|  | arginine | 20 |
|  | glycine | 20 |
|  | leucine | 20 |
|  | tryptophan | 20 |
|  | asparagine | 20 |
|  | lysine | 20 |
|  | cysteine | 20 |
|  | histidine | 20 |
|  | glutamine | 20 |
|  | alanine | 20 |
|  | glutamic acid | 20 |
|  | serine | 20 |
|  | tyrosine | 20 |
|  | valine | 20 |

| Nucleotide composition of the 20 *AA_i_tRNAs* | ATP | 25 |
| --- | --- | --- |
|  | GTP | 25 |
|  | CTP | 25 |
|  | UTP | 25 |
